# Comparison of the gut microbiota in older people with and without sarcopenia: a systematic review and meta-analysis

**DOI:** 10.3389/fcimb.2025.1480293

**Published:** 2025-04-28

**Authors:** Yanqing Ren, Xiangfeng He, Ling Wang, Nan Chen

**Affiliations:** ^1^ Department of Rehabilitation, Chongming Hospital Affiliated to Shanghai University of Medicine and Health Sciences, Shanghai, China; ^2^ Key Laboratory of Exercise and Health Sciences of Ministry of Education, Shanghai University of Sport, Shanghai, China; ^3^ Department of Rehabilitation, Xinhua Hospital Affiliated to Shanghai Jiaotong University School of Medicine, Shanghai, China

**Keywords:** sarcopenia, older people, gut microbiota, biomarker, systematic review, meta-analysis, nutrition

## Abstract

**Introduction:**

Sarcopenia, an age-related disorder marked by decreased skeletal muscle mass, strength, and function, is associated with negative health impacts in individuals and financial burdens on families and society. Studies have suggested that age-related alterations in gut microbiota may contribute to the development of sarcopenia in older people through the gut-muscle axis, thus modulation of gut microbiota may be a promising approach for sarcopenia treatment. However, the characteristic gut microbiota for sarcopenia has not been consistent across studies. Therefore, the aim of this study was to compare the diversity and compositional differences in the gut microbiota of older people with and without sarcopenia, and to identify gut microbiota biomarkers with therapeutic potential for sarcopenia.

**Methods:**

The PubMed, Embase, Web of Science, Cochrane Library, China National Knowledge Infrastructure, and Wanfang Database were searched studies about the gut microbiota characteristics in older people with sarcopenia. The quality of included articles was assessed by the Newcastle-Ottawa Scale (NOS). Weighted standardized mean differences (SMDs) and 95% confidence intervals (CIs) for α-diversity index were estimated using a random effects model. Qualitative synthesis was conducted for β-diversity and the correlation between gut microbiota and muscle parameters. The relative abundance of the gut microbiota was analyzed quantitatively and qualitatively, respectively.

**Results:**

Pooled estimates showed that α-diversity was significantly lower in older people with sarcopenia (SMD: -0.41, 95% CI: -0.57 to -0.26, I²: 71%, P < 0.00001). The findings of β-diversity varied across included studies. In addition, our study identified gut microbiota showing a potential and negative correlation with sarcopenia, such as Prevotella, Slackia, Agathobacter, Alloprevotella, Prevotella copri, Prevotellaceae sp., Bacteroides coprophilus, Mitsuokella multacida, Bacteroides massiliensis, Bacteroides coprocola Conversely, a potential and positive correlation was observed with opportunistic pathogens like Escherichia-Shigella, Eggerthella, Eggerthella lenta and Collinsella aerofaciens.

**Discussion:**

This study showed that α-diversity is decreased in sarcopenia, probably predominantly due to diminished richness rather than evenness. In addition, although findings of β-diversity varied across included studies, the overall trend toward a decrease in SCFAs-producing bacteria and an increase in conditionally pathogenic bacteria. This study provides new ideas for targeting the gut microbiota for the prevention and treatment of sarcopenia.

**Systematic review registration:**

https://www.crd.york.ac.uk/PROSPERO/view/CRD42024573090, identifier CRD42024573090.

## 1 Introduction

Sarcopenia, a geriatric and generalized disorder, is characterized by loss of skeletal muscle mass with low muscle strength and/or physical performance (Kirk et al., 2024). The global prevalence of age-related sarcopenia ranges from 10% to 27% in individuals over 60 years old (Petermann-Rocha et al., 2022). In China, the prevalence of sarcopenia is 20.7%, with the highest prevalence in people aged 80 years and older (45.4%), followed by people aged 70-79 years (27.2%) and 60-69 years (15.7%) (Meng et al., 2024). Sarcopenia is associated with an increased risk of various adverse outcomes such as falls and fractures (Roh et al., 2017), disability (Nascimento et al., 2019), cognitive impairment (Chang et al., 2016), cardiovascular diseases (Damluji et al., 2023), poor quality of life (Beaudart et al., 2015) and premature death (De Buyser et al., 2016), all of which impose a heavy economic burden on families and societies (Mijnarends et al., 2018). This highlights the urgent need for effective prevention and treatment strategies for sarcopenia. Therefore, it is necessary to seek an effective treatment for sarcopenia, and the modification of the gut microbiota shows significant promise (Zhang et al., 2022b).

The human gut microbiota is composed of 10-100 trillion microorganisms (Bakhtiar et al., 2013), which appears to play an important role in the muscle mass and function through regulating protein synthesis and degradation balance, systemic inflammation, glucose, lipid and energy metabolism, mitochondria and neuromuscular junction function (Liu et al., 2021a). Currently, the hypothesis of the “gut-muscle axis” has been proposed to study the relationship between the gut microbiota and musculoskeletal disorders (Bindels and Delzenne, 2013). Gut microbiota dysbiosis, is characterized by diminished biodiversity, higher pathogenic bacteria, lower beneficial bacteria, as well as the reduced expression of genes which produce short-chain fatty acids (SCFAs) (Buford, 2017; Wellman et al., 2017). This imbalance of gut microbiota is particularly prevalent in older people, attributed to age-related factors such as malnutrition, physical inactivity, chronic disease, and polypharmacy (Vaiserman et al., 2017; Ticinesi et al., 2019; Gemikonakli et al., 2021). Since gut dysbiosis can trigger adverse changes such as inflammation and anabolic resistance, age-related alterations in the gut microbiota have the potential to contribute to sarcopenia in the older people via the gut-muscle axis (Ticinesi et al., 2019). Therefore, identifying gut microbial markers of sarcopenia and targeting improvement of gut dysbiosis is a promising strategy for the treatment of sarcopenia.

So far, studies focusing on the gut microbiota characteristics in older people with sarcopenia have reached inconsistent and sometimes contradictory conclusions (Kang et al., 2021; Lee et al., 2022). Kang et al. reported a significant decrease in the abundance of Roseburia in older people with sarcopenia compared to non-sarcopenic individuals. In contrast, Lee et al. observed a significant increase in Roseburia abundance in sarcopenic individuals. This discrepancy may stem from methodological differences, particularly in study design. Kang et al. employed a case-control design, while Lee et al. utilized a cross-sectional approach. These differences in study design could have resulted in variations in data collection, sample selection, and analytical methods, which may have influenced the outcomes. Furthermore, the participants in the two studies differed in age, with the mean age of the sarcopenia group in Kang et al.’s study being 76.45 years, compared to 66.5 years in Lee et al.’s study. Age-related differences in body function and gut microbiota composition could have contributed to the observed discrepancies. Therefore, the methodological differences, particularly in study design and age, are likely key factors underlying the inconsistent findings between the two studies, which have impacted the comparability of their results. These inconsistent findings revealed the complexity of the relationship between the gut microbiota and sarcopenia, suggesting potential influences of study design, participants’ characteristics (e.g., age, gender, body composition, diet) and assessment methods on the observed discrepancies (Forrest et al., 2007; Beaudart et al., 2016; Martí et al., 2017; Deschasaux et al., 2018; Papadopoulou et al., 2021). Therefore, there is an urgent need to synthesize the existing studies and identify consistent gut microbiota characteristics associated with sarcopenia in older people. Despite the current studies that encompasses two systematic reviews separately investigating the changes in gut microbiota associated with muscle atrophy and frailty (Rashidah et al., 2022; Nikkhah et al., 2023), and a meta-analysis focusing on the characteristic alterations of the gut microbiota in frail older people (Almeida et al., 2022), these studies have not been directly targeted at the older people with sarcopenia.

This meta-analysis aims to bridge this gap by comparing the diversity and composition of the gut microbiota in older people with and without sarcopenia. Our goal is to identify gut microbiota profiles with therapeutic potential, thus providing new scientific insights into the diagnosis and treatment of sarcopenia.

## 2 Methods

### 2.1 Protocol and registration

This systematic review and meta-analysis was pre-registered in the International Prospective Register of Systematic Reviews (PROSPERO, CRD42024573090) and conducted in accordance with the guidelines of Preferred Reporting Items for Systematic Reviews and Meta-Analyses (PRISMA) (Hutton et al., 2015).

### 2.2 Search strategy

We searched and identified relevant studies using six databases in July 2024: PubMed, Embase, Web of Science, Cochrane Library, China National Knowledge Infrastructure, and Wanfang Database. The search strategy combined Medical Subject Headings (MeSH) terms and their synonyms related to sarcopenia (e.g., “sarcopenia” or “sarcopenic” or “muscular atrophy” or “muscle weakness”) and gut microbiota (e.g., “gastrointestinal microbiome” or “gastrointestinal microbiomes” or “fecal microbiota”). There is no restriction on the date of publication. See details in **Appendix S1** in **Supplementary Material**.

### 2.3 Eligibility criteria

#### 2.3.1 Inclusion criteria

1. Participants: Individuals diagnosed with sarcopenia according to any established definitions (by a working group or a clinical research), aged 60 years or older, of both genders;
2. Outcomes: Studies documenting variations in microbiota diversity (α-diversity or/and β-diversity) and composition between sarcopenia and non-sarcopenia groups.

#### 2.3.2 Exclusion criteria

1. Studies did not include a full-text description;
2. Studies were not in English or Chinese languages;
3. Studies were non-original researches such as reviews, conference reports, letters, case reports and commentaries;
4. Studies had unextractable data information; and
5. Participants received interventions affecting the gut microbiota within one month.

### 2.4 Study selection

The records sourced from various databases were consolidated within EndNote 20 (Clarivate Analytics in Philadelphia, PA, USA), where duplicate entries were automatically detected and eliminated. Two reviewers (YR and LW) independently conducted an assessment of the titles and abstracts according to the inclusion and exclusion criteria, followed by a thorough examination of the full texts to ascertain the studies eligible for inclusion. If there were discrepancies between the two reviewers, a third reviewer, XH, mediated the discussion to reach a consensus.

### 2.5 Data extraction

Data extraction was performed independently by two researchers (YR and LW), with cross-verification, and subsequently validated by a third researcher (XH).

Data extraction included the following variables:

1. Study characteristics (such as first author, publication year, the country and region where the data were collected, study design, sample size, diagnostic criteria of sarcopenia, stool sample collection and storage, and assessment method of gut microbiota);
2. Participants’ characteristics (such as age, gender and BMI);
3. Community-level measures of gut microbiota composition: α-diversity (Chao1 index, Observed species/OTUs, Shannon index, Simpson index and ACE index), β-diversity, and taxonomic findings at the phylum, class, order, family, and genus levels (relative abundance).

We consulted the authors of the included studies for raw data about specific α-diversity and relative abundance data that were not showed in the paper. For studies which raw data were not available or data cannot be processed, we employed WebPlot Digitizer 4.7 software to extract numerical data from the figures of the studies. The variables data of α-diversity and relative abundance were presented as means (M) and standard deviation (SD). When included studies presented variables data as median and interquartile range (IQR), we utilized an online tool (https://www.math.hkbu.edu.hk/~tongt/papers/median2mean.html) to convert these data to M and SD.

### 2.6 Quality assessment

The Newcastle-Ottawa Scale (NOS) was performed in case-control and cohort studies, and a modified version of the NOS for cross-sectional studies (Benites-Zapata et al., 2022). The NOS scale consists of three assessment areas: selection, comparability, and exposure/outcome; with a maximum score of 9 for case-control and cohort studies, and 7 for cross-sectional studies. Case-control and cohort studies with a total score of ≥ 7 and cross-sectional studies with a total score of ≥ 4 are considered high-quality studies (Yeung et al., 2019).

### 2.7 Statistical analysis

#### 2.7.1 Quantitative synthesis of α-diversity

Various α-diversity indices were used in the included studies, including the Chao 1 index, Observed species/OTUs, the Shannon index, the Simpson index and ACE index. The pooled effect sizes were estimated using the inverse variance method as the primary statistical approach. In addition, a random effects model was used to account for heterogeneity across studies and the 95% confidence interval (CI) was calculated for the effect measure reported as standardized mean difference (SMD).

Subgroup analyses were performed based on categorical variables: age (<70 or ≥70 years old), gender (both or female), BMI (< 24.5 or ≥ 24.5 kg/m²), nutrition status (at a risk of malnutrition/malnutrition or healthy), diagnostic criteria for sarcopenia [European Working Group on Sarcopenia in Older People (EWGSOP), Asian Working Group for Sarcopenia (AWGS) or other], evaluation method for muscle mass [Bioelectrical Impedance Analysis (BIA), Dual-energy X-ray Absorptiometry (DXA) or other], region (Western countries or Eastern countries).

We used I² statistics to evaluate the heterogeneity of each outcome included in the study (Higgins and Thompson, 2002). I² > 50% indicates significant heterogeneity (Wang et al., 2022b). The presence of publication bias was assessed through a dual approach: a subjective evaluation of the symmetry in the funnel plot and a statistical assessment using Egger’s test. Sensitivity analyses were conducted to assess the stability of the findings by sequentially excluding individual studies from the meta-analysis (Duval and Tweedie, 2000). A result was deemed less robust if the exclusion of a study caused the pooled effect size to lie beyond the 95% confidence interval. Conversely, the results were classified as robust if they remained within this range.

Review Manager software (RevMan 5.4; Cochrane, Linden, UK) was used to perform subgroup analyses and STATA MP 17 software (STATACorp LP, College Station, Texas, USA) was used to test for publication bias and perform sensitivity analyses. P < 0.05 was considered statistically significant in all analyses.

#### 2.7.2 Qualitative synthesis of β-diversity

We summarized the β-diversity indicators, statistical analysis methods, findings regarding significant differences between groups, and the reported p-values across the included studies.

#### 2.7.3 Quantitative/Qualitative synthesis of relative abundance

##### 2.7.3.1 Quantitative synthesis

We recorded quantitative comparisons of the relative abundance of bacterial phyla, class, order, family, genus, and species between the sarcopenia and non-sarcopenia groups, including the p-values of these comparisons.

##### 2.7.3.2 Qualitative synthesis

We identified gut microbes showing significant differences (p < 0.05) in relative abundance between the sarcopenia and non-sarcopenia groups and noted their changes. In addition, we further summarized the microbes recorded in two or more studies.

### 2.8 Correlation between gut microbiota and sarcopenia parameters

We recorded gut microbes with significant correlations (p < 0.05) to muscle parameters (muscle mass, muscle strength and muscle function) from the included studies. Muscle mass was represented by three indices: skeletal muscle index (SMI), appendicular skeletal muscle index (ASMI) and skeletal muscle mass (SMM). Muscle strength was measured by handgrip strength (HGS). Muscle function was assessed by five-time chair stand test (5-STS) and gait speed (GS).

In this study, we defined the “Final relevance” between gut microbiota and muscle parameters based on the following criteria. When a microbe showed a significant correlation with only a single muscle parameter, the correlation was considered “Final relevance”. If a microbe showed significant correlations with multiple muscle parameters and these correlations were consistent in direction, the consistent correlation was recognized as “Final relevance”. However, if the direction of the correlations was inconsistent, we defined the “Final relevance” as “uncertain”.

### 2.9 Definition of gut microbiota with potential relevance to sarcopenia

In our study, we established criteria to identify gut microbiota that exhibit a potential correlation with sarcopenia.

A microbe was classified as potentially positive (or negative) correlation with sarcopenia if it satisfies either of the following conditions:

(1) Consistency in significant changes across studies: The microbe was consistently reported as significantly increased (or decreased) in the sarcopenia groups across two or more included studies;

(2) Consistent correlations with muscle parameters: The microbe was reported as significantly increased (or decreased) in the sarcopenia groups in at least one study, and concurrently shows a negative (or positive) “Final relevance” with muscle parameters.

A microbe was classified as having a potential but unclear correlation with sarcopenia if it satisfies either of the following conditions:

1. Inconsistency in significant changes across studies: The microbe was reported as significantly changed in the sarcopenia groups across two or more included studies, but the direction of these changes was inconsistent;
2. Inconsistent correlations with muscle parameters: The microbe showed “Final relevance” as “uncertain” with muscle parameters.

## 3 Results

### 3.1 Study selection

**Figure 1:**
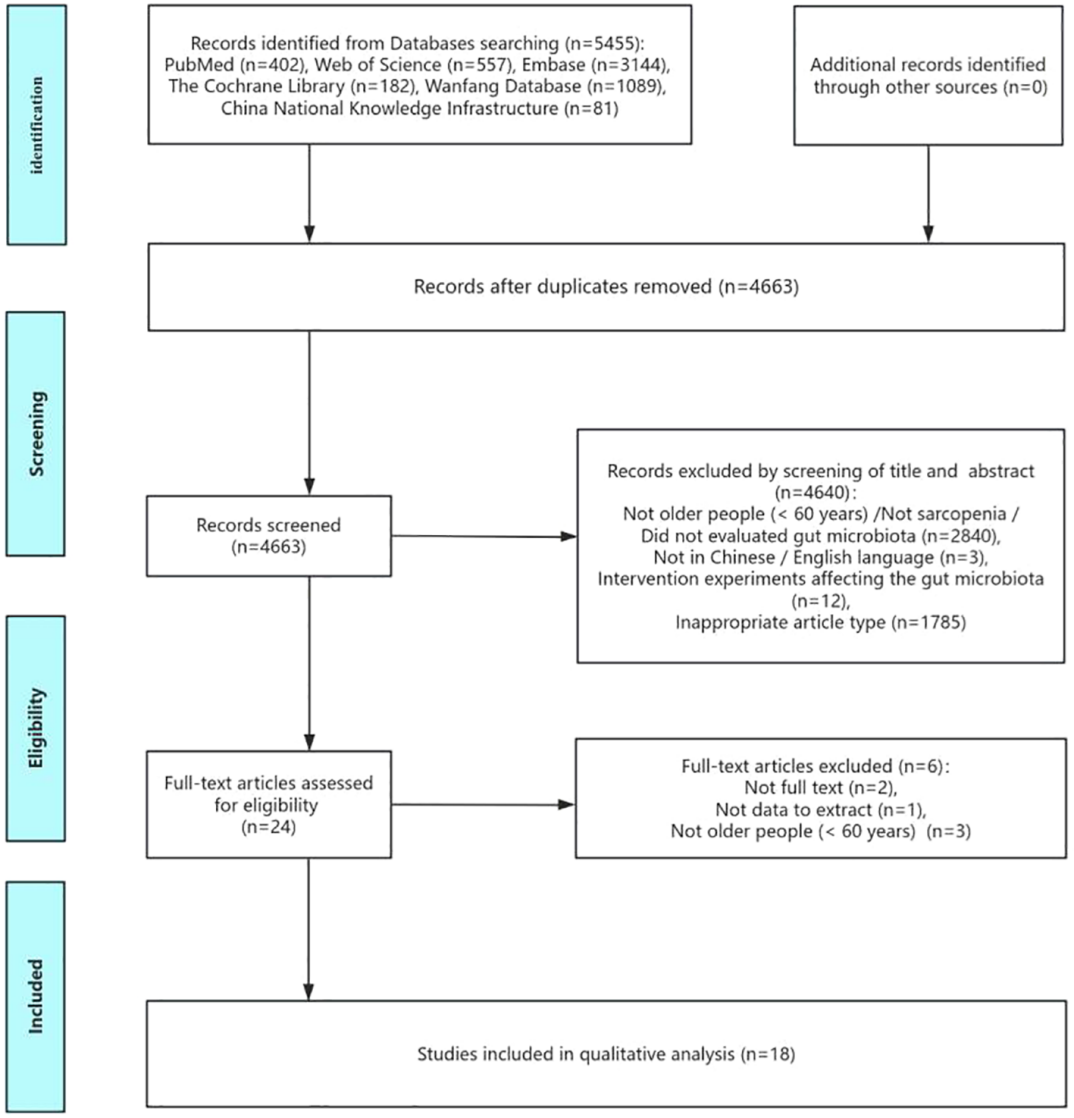
Flow of screening and selecting process according to Preferred Reporting Items for Systematic Reviews and meta-analysis (PRIAMA).

According to comprehensive literature search strategy, 5454 relevant articles were retrieved from six electronic databases. 4663 relevant articles were obtained when duplications were excluded. After reading the titles and abstracts, 23 studies were potentially eligible according to the inclusion and exclusion criteria. After carefully examining the full texts, the remaining 18 articles were included in the final meta-analysis. The literature screening process is shown in **Figure 1**.

### 3.2 Study characteristics

**Table 1** summarized the characteristics of the 18 studies included studies between 2019 and 2024 (Picca et al., 2019; Ticinesi et al., 2020; Kang et al., 2021; Margiotta et al., 2021; Ponziani et al., 2021; Han et al., 2022; Lee et al., 2022; Wang et al., 2022c; Wu et al., 2022; Yamamoto et al., 2022; Aliwa et al., 2023; Lee et al., 2023; Liu et al., 2023; Peng et al., 2023; Wang et al., 2023; Yan et al., 2023; Zhang et al., 2023, 2024), including two cohort studies (Aliwa et al., 2023; Lee et al., 2023), seven case-control studies (Kang et al., 2021; Ponziani et al., 2021; Wu et al., 2022; Yamamoto et al., 2022; Wang et al., 2023; Zhang et al., 2023, 2024) and nine cross-sectional studies (Picca et al., 2019; Ticinesi et al., 2020; Margiotta et al., 2021; Han et al., 2022; Lee et al., 2022; Wang et al., 2022c; Liu et al., 2023; Peng et al., 2023; Yan et al., 2023). The studies were conducted in five countries, which were categorized into Western countries (Austria, Italy) (Picca et al., 2019; Ticinesi et al., 2020; Margiotta et al., 2021; Ponziani et al., 2021; Aliwa et al., 2023) and Eastern countries (China, Korea, Japan) (Kang et al., 2021; Han et al., 2022; Lee et al., 2022; Wang et al., 2022c; Wu et al., 2022; Yamamoto et al., 2022; Lee et al., 2023; Liu et al., 2023; Peng et al., 2023; Wang et al., 2023; Yan et al., 2023; Zhang et al., 2023, 2024). In terms of gender, 16 studies included both males and females (Picca et al., 2019; Ticinesi et al., 2020; Kang et al., 2021; Margiotta et al., 2021; Ponziani et al., 2021; Han et al., 2022; Lee et al., 2022; Wang et al., 2022c; Wu et al., 2022; Yamamoto et al., 2022; Aliwa et al., 2023; Lee et al., 2023; Liu et al., 2023; Peng et al., 2023; Zhang et al., 2023, 2024), while two studies were limited to females (Wang et al., 2023; Yan et al., 2023). The studies comprised a total of 3,132 participants, 886 with sarcopenia and 2,246 without sarcopenia. The mean age of the participants ranged from 49.81 to 83.1 years. In addition, 29.4% of the participants had comorbidities( (Margiotta et al., 2021; Ponziani et al., 2021; Yamamoto et al., 2022; Aliwa et al., 2023; Lee et al., 2023; Peng et al., 2023).

**Table 1:** Characteristics of the studies included.

First Author, Year	Comorbidity	Setting	Age (Mean ± SD)	BMI (Mean ± SD)	Gender	Nutrition assessment	Country	N/M/F	Sarcopenia criteria	Cut-off	Methods of IM assessment
Aliwa et al., 2023	Cirrhosis	Hospital	Cirrhosis with S 64 (61; 68) Cirrhosis without S 62 (60; 67)	Cirrhosis with S 25.7 (24.2; 27.1) Cirrhosis without S 29.5 (27.4; 31.2)	Both	ND	Austria	116/86/30	EWGSOP 2010	1) L3-muscle area of ≤ 52.4 cm²/m² (M) and ≤ 38.5 cm²/m² (F) 2) HGS < 27 kg (M) and < 16 kg (F) 3) GS ≤ 0.8 m/s	16S rDNA sequencing of V1–V2 for α diversity, β diversity and relative abundance
Han et al., 2022	ND	Community	LM 72.3 ± 5.4 NM 70.0 ± 4.2	LM 19.7 ± 1.7 NM 22.5 ± 2.2	Both	MNA↓*	China	88/28/60	IWGS	ASMI < 7.23 kg/m² (M) and < 5.67 kg/m² (F)	16S rRNA sequencing of V3-V4 for α diversity, β diversity and relative abundance
Kang et al., 2021	ND	Hospital	S 76.45 ± 8.58 NS 68.38 ± 5.79	S 20.67 ± 3.27 NS 23.66 ± 2.49	Both	ND	China	87/36/51	AWGS 2019	1) ASMI < 7.0 kg/m² (M) or < 5.7 kg/m² (F) 2) HGS < 28 kg (M) or < 18 kg (F) 3) 5-STS ≥ 12s	16S rRNA sequencing of V3-V4 for α diversity, β diversity and relative abundance
Lee et al., 2023	Cirrhosis	Hospital	Cirrhosis with S 62.7 (54.3–66.5) NS 58.4 (49.6–64.5)	cirrhosis with S 22.4 (21.1–23.6) Healthy controls 23.3 (22.3–24.7)	Both	SGA↑* MNA↓* MUST	China	105/82/23	AWGS 2019	1) ASMI < 7.0 kg/m² (M) and < 5.4 kg/m² (F) 2) HGS < 28 kg (M) and < 18 kg (F)	16S rRNA sequencing of V3-V4 for α diversity, β diversity and relative abundance
Lee et al., 2022	ND	Community	S 66.5 ± 4.6 NS 64.8 ± 3.4	S 23.0 ± 3.4 NS 26.4 ± 3.5	Both	24 h dietary recall	Korea	60/15/45	AWGS 2019	1) ASMI < 7.0 kg/m² (M) and < 5.7 kg/m² (F) 2) HGS < 28 kg (M) and < 18 kg (F) 3) GS < 1 m/s	16S rRNA sequencing of V3-V4 for α diversity, β diversity and relative abundance
Liu et al., 2023	ND	Community	S 69.1 ± 8.0 NS 64.1 ± 9.2	S 22.5 ± 2.7 NS 26.2 ± 3.4	Both	ND	China	283/88/195	AWGS 2014	1) ASMI < 7.0 kg/m² (M) and < 5.7 kg/m² (F) 2) HGS < 26 kg (M) and <18 kg (F) 3) GS < 0.8 m/s	Shotgun metagenomic sequencing for α diversity, β diversity and relative abundance
Margiotta et al., 2021	CKD	Community	S 83.1 ± 5.7 NS 79.7 ± 6.2	S 25.5 ± 2.6 NS 29.3 ± 4.8	Both	MIS↑	Italy	63/44/19	EWGSOP 2	ND	16S rRNA sequencing of V3-V4 for relative abundance
Peng et al., 2023	HF	Hospital	HF 71.76 ± 7.93 SHF 75.14 ± 8.18 HC 67.67 ± 9.76	HF 24.24 ± 2.81 SHF 20.27 ± 3.75 HC 23.52 ± 3.12	Both	ND	China	77/45/32	AWGS 2019	1) ASMI < 7.0 kg/m² (M) or < 5.7 kg/m² (F) 2) HGS < 28 kg (M) or < 18 kg (F) 3) GS < 1 m/s	16S rRNA sequencing of V3-V4 for α diversity, β diversity and relative abundance
Picca et al., 2019	ND	Community	PF&S 75.5 ± 3.9 NonPF&S 73.9 ± 3.2	PF&S 32.14 ± 6.02 NonPF&S 26.27 ± 2.55	Both	ND	Italy	35/20/15	FNIH	1) SPPB score between 3/12 and 9/12 2) (a) ALM/BMI < 0.789 (M) and < 0.512(F) or (b) crude ALM < 19.75 kg (M) and < 15.02 kg (F) 3) Absence of mobility disability	16S rRNA sequencing of V3-V4 for α diversity and relative abundance
Ponziani et al., 2021	Cirrhosis	Community	Cirrhosis with S 70 (63-74) Cirrhosis without S 66 (58.5-76.5) Control with S 75.5 (72-77.25) Control without S 72.5 (58.2575.25)	Cirrhosis with S 29 (25.48-30.91) Cirrhosis without S 27.27 (24.36-29.12) Controls with S 29.99 (29-31.79) Controls without S 26.2 (24.39-28.68)	Both	7day FFQ: red meat(times/wk), non-red meat(times/wk), milk(times/wk)↔, fish(times/wk)↑,eggs(times/wk)↑, cereals and bakery products(times/wk)↔, legumes(times/wk)↔,Veg(times/wk)↔, fruits(times/wk)↔	Italy	100/36/64	FNIH	1) (a) ALM/BMI < 0.789 (M) and < 0.512 (F) or (b) crude ALM < 19.75 kg (M) and < 15.02 kg (F) 2) HGS < 26 kg (M) and <16 kg (F) 3) Absence of mobility disability	16S rRNA sequencing of V3-V4 for α diversity, β diversity and relative abundance
Ticinesi et al., 2020	ND	Community	S 77 (75.5-86) NS 71.5 (70-75)	S 24.3 (20.9–26.7) NS 27.4 (24.5–29.1)	Both	EPIC FFQ: total P(g)↓, animal P(g)↓, vegetal P(g)↑, total F(g)↓, animal F(g)↓, vegetal F(g)↓, total SFA(g)↓, total PUFA(g)↓, sugar(g)↑, fiber(g)↑, E(Kcal)↓, Fe(mg)↓, Ca(mg)↓, Zn(mg)↓, Vit C(mg)↓,Vit B6(mg)↓, β Carotene(μg)↓, Vit E(μg)↓, Vit D(mg)↓	Italy	17/3/14	EWGSOP 1	1) SPPB score between 3/12 and 9/12 2) SMI < 8.87 kg/m² (M) and < 6.42 kg/m² (F)	Shallow-shotgun metagenomics for β diversity and relative abundance
Wang et al., 2022c	ND	Community	S 72.2 ± 8.5 NS 62.3 ± 8.5	S 21.4 ± 2.5 NS 24.2 ± 3.4	Both	SFFQ: meat/eggs(times/wk)↓, dairy products(times/wk)↓, Veg(times/wk)↓	China	1417 /582 /835	AWGS 2019	1) ASMI < 7.0 kg/m² (M) or < 5.7 kg/m² (F) 2) HGS < 28 kg (M) and < 18 kg (F) 3) 2) SPPB score ≤ 9; 5-STS ≥ 12 s; or GS < 1.0 m/s	Shotgun metagenomic sequencing for α diversity, β diversity and relative abundance
Wang et al., 2023	ND	Community	S 68.4 ± 3.5 NS 68.7 ± 3.4	S 22.5 ± 2.3 NS 24.1 ± 2.4	Female	FFQ: E(kj)↓*, total P(g)↓*, HQ P(g)↓*, F(g)↓, Ca(mg)↓, Vit D(IU)↔, soybean products(g)↑, dairy products(g)↓, Veg(g)↓, fruit(g)↓, meat(g)↓, fish(g)↓	China	100/0/100	EWGSOP 2018 + AWGS 2019	1) SARC-F questionnairea score ≥ 4 2) GS < 1.0 m/s 3) HGS ≤ 18 kg (F) 4) ASMI < 5.7 kg/m² (F)	Metagenomic sequencing for relative abundance
Wu et al., 2022	ND	Hospital	S 77(65-95) NS 70(65-84)	S 22.87 ± 3.17 NS 23.52 ± 30.39	Both	ND	China	192/87 /105	EWGSOP 2	1) muscle strength < 27 kg 2) ASM < 20 kg 3) SPPB score ≤ 8	16S rRNA sequencing of V3-V4 for α diversity and relative abundance
Yamamoto et al., 2022	CLD	Hospital	N-SMI 66 (57.5–71.5) LSMI 68 (62.0–73.3)	ND	Both	Questionnaire on dietary lifestyle: meat/fish(g)↓, Veg(g)↓	Japan	69/25/44	Other	SMI < 42 cm²/m² (M) and 38 cm²/m² (F)	16S rRNA sequencing of V3-V4 for α diversity, β diversity and relative abundance
Yan et al., 2023	ND	Community	S 75.3 ± 7.14 NS 70.26 ± 6.03	S 23.60 ± 3.07 NS 25.88 ± 3.67	Female	1) MNA↓* 2) FFQ:E(kcal/d)↓, P(g/kg/d)↓*, F(g/d)↓*, CHO(g/d)↓,fiber(g/d)↓*, Vit C(mg/d)↓, Vit E(mg/d)↓*, Ca(mg/d)↓, Mg(mg/d)↓*, Fe(mg/d)↓*, Zn(mg/d)↓*,	China	276/0/276	AWGS 2019	1) ASMI < 5.7 kg/m² 2) HGS < 18 kg 3) GS < 1.0 m/s	16S RNA sequencing without region information for α diversity, β diversity and relative abundance
Zhang et al., 2023	ND	Hospital	S 71.21 ± 6.85 NS 71.00 ± 6.67	S 22.83 ± 1.91 NS 23.57 ± 2.18	Both	ND	China	35/10/25	AWGS 2019	1) ASMI < 7.0 kg/m² (M) and < 5.4 kg/m² (F) 2) HGS < 28 kg (M) and < 18 kg (F) 3) GS < 1.0 m/s	16S rRNA sequencing of V4 for α diversity, β diversity and relative abundance
Zhang et al., 2024	ND	Community	S 55.39 ± 7.59 NS 49.81 ± 6.84	S 21.02 ± 1.37 NS 22.20 ± 1.26	Both	ND	China	62/30/32	AWGS 2019	1) ASMI < 7.0 kg/m² (M) and < 5.7 kg/m² (F) 2) HGS < 28 kg (M) and < 18 kg (F) 3) GS < 1 m/s	16S rRNA sequencing of V4 for α diversity, β diversity and relative abundance

SD, ± standard; 16S rRNA, 16S Ribosomal Ribonucleic Acid; V1-V2, regions of the 16S rRNA gene; V1-V9, regions of the 16S rRNA gene; V3-V4, regions of the 16S rRNA gene; V4, regions of the 16S rRNA gene; S, sarcopenia; NS, Non-sarcopenia; PF&S, Physical frailty and sarcopenia; Non PF&S, Not Physical frailty or sarcopenia; CKD, chronic Kidney Disease; HF, heart failure; SHF, heart failure patients with sarcopenia; CLD, chronic liver disease; LM, low muscle mass group; NM, normal muscle mass group; MNA, mini nutritional assessment; SGA, subjective global assessment; MUST, malnutrition universal screening tool; MIS, malnutrition inflammation score; FFQ, food frequency questionnaires; EPIC, European Prospective Investigation into Cancer and Nutrition; SFFQ, semi-quantitative food frequency questionnaire; ↑, denote increases in evaluated outcome measures in sarcopenia compared to control group; ↓, denote decreases in evaluated outcome measures in sarcopenia compared to control groups; ↔, denote comparable evaluated outcome measures between sarcopenia and control groups; *, denote statistically significant difference evaluated outcome between sarcopenia and control groups; wk, week; d, day; h, hour; g, gram; mg, milligram; μg, microgram; IU, international unit; RAE, Retinol Activity Equivalent; kcal, kilocalories; kj, kilojoule; Veg, vegetables; P, protein; F, fat; SFA, saturated fatty acids; PUFA, polyunsaturated fatty acids; E, energy; Fe, iron; Ca, calcium; K, potassium; Zn, zinc; Vit, vitamin; HQ, high-quality; CHO, carbohydrate; Mg, magnesium; Zn, zinc; EWGSOP, European Working Group on Sarcopenia in Older People; IWGS, International Working Group on Sarcopenia; AWGS, Asian Working Group for Sarcopenia Guidelines; FNIH, Foundation for the National Institutes of Health sarcopenia project; M, men; F, female; ASM, appendicular skeletal muscle mass; ASMI, appendicular skeletal muscle mass index; SMM, skeletal muscle mass; SMI, skeletal muscle index; HGS, handgrip strength; GS, gait speed; SPPB, short-physical performance battery; 5-STS, five times sit to stand test; ALMBMI, appendicular lean mass to body mass index ratio; ND, No data.

Ten studies evaluated the dietary and nutritional status of the participants using various scales (Ticinesi et al., 2020; Margiotta et al., 2021; Ponziani et al., 2021; Han et al., 2022; Lee et al., 2022; Wang et al., 2022c; Yamamoto et al., 2022; Lee et al., 2023; Wang et al., 2023; Yan et al., 2023). Tools such as the Mini-Nutritional Assessment (MNA), Malnutrition Universal Screening Tool (MUST), and Malnutrition Inflammation Score (MIS) were utilized to directly assessed nutritional status. Furthermore, dietary intake was evaluated using the Food Frequency Questionnaire (FFQ), 24-hour dietary recall, and Dietary Lifestyle Questionnaire (DLQ), with the findings used in conjunction with the Dietary Guidelines for Chinese Residents (DGCR) to indirectly infer participants’ nutritional status (Zhang et al., 2022a). Exception for two studies that lacked specific assessment results (Margiotta et al., 2021; Lee et al., 2022), the remaining eight studies ultimately identified nutritional status (Ticinesi et al., 2020; Ponziani et al., 2021; Han et al., 2022; Wang et al., 2022c; Yamamoto et al., 2022; Lee et al., 2023; Wang et al., 2023; Yan et al., 2023), with five indicating risks of malnutrition (Margiotta et al., 2021; Han et al., 2022; Wang et al., 2022c, 2023; Yan et al., 2023).

The majority of studies adhered to AWGS (Kang et al., 2021; Lee et al., 2022; Wang et al., 2022c; Lee et al., 2023; Liu et al., 2023; Peng et al., 2023; Wang et al., 2023; Yan et al., 2023; Zhang et al., 2023, 2024) and EWGSOP (Ticinesi et al., 2019; Margiotta et al., 2021; Wu et al., 2022; Aliwa et al., 2023; Wang et al., 2023) criteria for diagnosing sarcopenia. Gut microbiota was analyzed using 16S rRNA sequencing in 14 studies (Picca et al., 2019; Kang et al., 2021; Margiotta et al., 2021; Ponziani et al., 2021; Han et al., 2022; Lee et al., 2022; Wu et al., 2022; Yamamoto et al., 2022; Aliwa et al., 2023; Lee et al., 2023; Peng et al., 2023; Yan et al., 2023; Zhang et al., 2023, 2024), and shotgun sequencing in four studies (Ticinesi et al., 2020; Wang et al., 2022c; Liu et al., 2023; Wang et al., 2023). The relative abundance of gut microbiota was assessed in all 18 included studies (Picca et al., 2019; Ticinesi et al., 2020; Kang et al., 2021; Margiotta et al., 2021; Ponziani et al., 2021; Han et al., 2022; Lee et al., 2022; Wang et al., 2022c; Wu et al., 2022; Yamamoto et al., 2022; Aliwa et al., 2023; Lee et al., 2023; Liu et al., 2023; Peng et al., 2023; Wang et al., 2023; Yan et al., 2023; Zhang et al., 2023, 2024), with 15 studies assessing α-diversity (Picca et al., 2019; Kang et al., 2021; Ponziani et al., 2021; Han et al., 2022; Lee et al., 2022; Wang et al., 2022c; Wu et al., 2022; Yamamoto et al., 2022; Aliwa et al., 2023; Lee et al., 2023; Liu et al., 2023; Peng et al., 2023; Yan et al., 2023; Zhang et al., 2023, 2024) and 14 studies assessing β-diversity (Ticinesi et al., 2020; Kang et al., 2021; Ponziani et al., 2021; Han et al., 2022; Lee et al., 2022; Wang et al., 2022c; Yamamoto et al., 2022; Aliwa et al., 2023; Lee et al., 2023; Liu et al., 2023; Peng et al., 2023; Yan et al., 2023; Zhang et al., 2023, 2024). Details of fecal processing and DNA extraction methods in the different studies are given in **Supplementary Table S1**.

### 3.3 Quality assessment

The majority of the studies included in this meta-analyses presented a high-quality score on NOS. One cohort study (Lee et al., 2023) and five case-control studies (Kang et al., 2021; Ponziani et al., 2021; Wu et al., 2022; Wang et al., 2023; Zhang et al., 2023) scored ≥ 7 points, and nine cross-sectional studies (Picca et al., 2019; Ticinesi et al., 2019; Margiotta et al., 2021; Han et al., 2022; Lee et al., 2022; Wang et al., 2022c; Liu et al., 2023; Peng et al., 2023; Yan et al., 2023) scored ≥ 4 points, which were of high quality. The remaining studies were of average medium quality: one cohort study (Aliwa et al., 2023) and two case-control studies (Yamamoto et al., 2022; Zhang et al., 2024) had a score of 6 points (**Tables 2**–**4**).

**Table 2:** Newcastle-Ottawa Scale assessment cohort studies.

First author, year	Selection				Comparability	Outcome			Total	Quality
	1	2	3	4	5	6	7	8		
Aliwa et al., 2023	*	*	*	*	–	*	*	–	6	M
Lee et al., 2023	*	*	*	*	*	*	*	–	7	H

*One point attributed in the question; -: None point attributed in the question; 1: Representativeness of the exposed cohort with sarcopenia; 2: Ascertainment of exposure: how is sarcopenia diagnosis made; 3: Selection of the non-exposed cohorts; 4: Demonstration of normal gut microbiota at start of study; 5: Comparability of cohorts on the basis of the design or analysis controlled for confounders; 6: Assessment of gut microbiota outcome; 7: Was follow-up long enough for gut microbiota outcomes to occur; 8: Adequacy of follow-up of cohorts; H: High-quality; M: Medium-quality.

**Table 3:** Newcastle-Ottawa Scale assessment case control studies.

First author, year	Selection				Comparability	Outcome			Total	Quality
	1	2	3	4	5	6	7	8		
Kang et al., 2021	*	*	*	*	–	*	*	*	7	H
Ponziani et al., 2021	*	*	*	*	*	*	*	–	7	H
Wang et al., 2023	*	*	*	*	*	*	*	–	7	H
Wu et al., 2022	*	*	*	–	**	*	*	*	7	H
Yamamoto et al., 2022	–	–	*	*	**	*	*	–	6	M
Zhang et al., 2023	*	*	*	*	*	*	*	–	7	H
Zhang et al., 2024	*	*	*	*	–	*	*	–	6	M

*One point attributed in the question; **Two points attributed in the question; -: None point attributed in the question; 1: Representativeness of the cases; 2: Is the case definition adequate; 3: Selection of controls; 4: Definition of controls; 5: Comparability of cases and controls on the basis of the design or analysis; 6: Ascertainment of exposure; 7: Same method of ascertainment for cases and controls; 8: Non-response rate; H: High-quality; M: Medium-quality.

**Table 4:** Newcastle-Ottawa Scale assessment cross-sectional studies.

First author, year	Selection			Comparability	Outcome		Total	Quality
	1	2	3	4	5	6		
Han et al., 2022	*	*	*	–	*	*	5	H
Lee et al., 2022	*	*	*	*	*	–	5	H
Liu et al., 2023	*	*	*	–	*	–	4	H
Margiotta et al., 2021	*	*	*	*	*	–	5	H
Peng et al., 2023	*	*	*	*	*	–	5	H
Picca et al., 2019	*	*	*	*	*	*	6	H
Ticinesi et al., 2020	*	*	*	**	*	*	7	H
Wang et al., 2022c	*	*	*	–	*	*	5	H
Yan et al., 2023	*	*	*	–	*	*	5	H

*One point attributed in the question; **Two points attributed in the question; -: None point attributed in the question; 1: Representativeness of the sample; 2: Selection of the non-exposed subjects; 3: Ascertainment of exposure: how is sarcopenia diagnosis made; 4:The subjects in different outcome groups are comparable, based on the study design or analysis. Confounding factors are controlled; 5: Assessment of gut microbiota outcome; 6: Response rate; H: High-quality.

### 3.4 Quantitative synthesis of α-diversity

#### 3.4.1 Meta-analysis summary

A total of 1167 sarcopenic and 2566 non-sarcopenic older people were included in 15 studies assessing α-diversity. Various α-diversity indices were used in the studies, including the Chao 1 index (Picca et al., 2019; Kang et al., 2021; Ponziani et al., 2021; Han et al., 2022; Wu et al., 2022; Yamamoto et al., 2022; Aliwa et al., 2023; Peng et al., 2023; Yan et al., 2023; Zhang et al., 2023, 2024), Observed species/OTUs (Kang et al., 2021; Han et al., 2022; Wu et al., 2022; Yamamoto et al., 2022; Peng et al., 2023), the Shannon index (Han et al., 2022; Lee et al., 2022; Wang et al., 2022c; Yamamoto et al., 2022; Lee et al., 2023; Liu et al., 2023; Peng et al., 2023; Yan et al., 2023; Zhang et al., 2023, 2024), the Simpson index (Lee et al., 2022; Peng et al., 2023; Yan et al., 2023; Zhang et al., 2023, 2024) and ACE index (Yan et al., 2023; Zhang et al., 2024).

**Figure 2:**
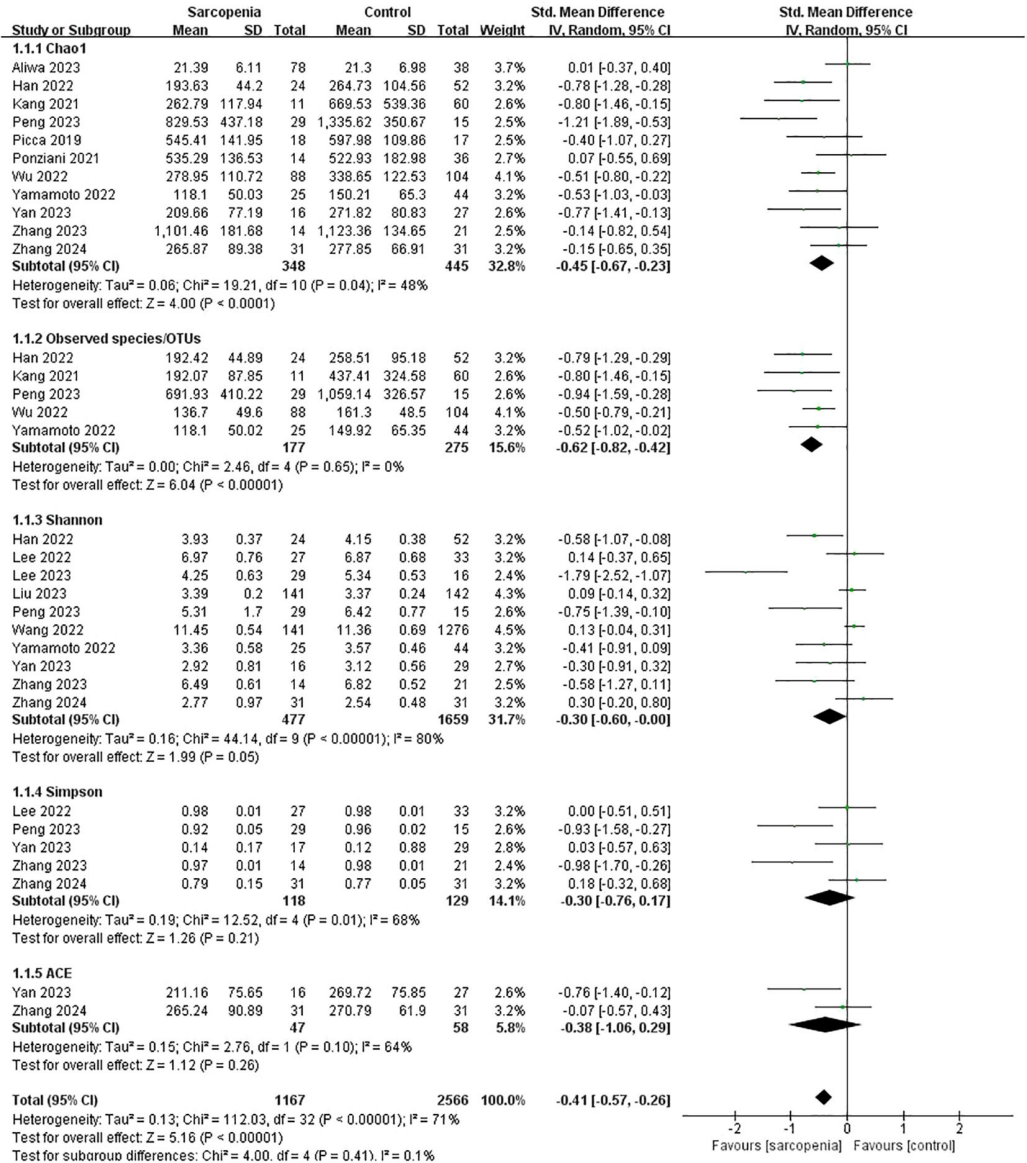
α-diversity forest plots of sarcopenia compared with non-sarcopenia in total and divided subgroups: Chao1 index, Observed species/OTUs, Shannon index, Simpson index and ACE index.

Pooled estimates showed that α-diversity was significantly lower in older people with sarcopenia than without sarcopenia, with significant heterogeneity (SMD: -0.41, 95% CI: -0.57 to -0.26, I²: 71%, P < 0.00001). To be specific, the Chao1 index (SMD: -0.45, 95% CI: -0.67 to -0.23, I²: 48%, p < 0.0001), Observed species/OTUs (SMD: -0.62, 95% CI: -0.82 to -0.42, I²: 0%, p < 0.00001) and the Shannon index (SMD: -0.30, 95% CI: -0.60 to -0.00, I²: 80%, p = 0.05) were significantly lower in the sarcopenia groups. However, there were no significant differences in the Simpson index (SMD: -0.30, 95% CI: -0.76 to 0.17, I²: 68%, p = 0.21) or the ACE index (SMD: -0.38, 95% CI: -1.06 to 0.29, I²: 64%, p = 0.26) between the two groups (**Figure 2**).

#### 3.4.2 Subgroup analyses

According to participants’ characteristics and study design, we conducted subgroup analyses of various α-diversity indexes including the Chao1 index, Observed species/OTUs, Shannon index, and Simpson index (**Table 5**).

**Table 5:** Subgroup analysis of the alpha diversity of the gut microbiota in patients with sarcopenia.

Variable	Subgroup	Studies	N	SMD [95% CI]	Heterogeneity				Test overall effects. Z (p)	Test for Subgroup Difference. Chi² (p)
					Tau²	Chi²	P	I²		
α diversity										
α diversity indicator	Chao1	11	793	-0.45 [-0.67, -0.23]	0.06	19.21	0.04	48%	4.00 (< 0.0001)	4.00 (0.41)
	Observed species/OTUs	5	452	-0.62 [-0.82, -0.42]	0.00	2.46	0.65	0%	6.04 (< 0.00001)	
	Shannon	10	2136	-0.30 [-0.60, -0.00]	0.16	44.14	<0.00001	80%	1.99 (0.05)	
	Simpson	5	247	-0.30 [-0.76, 0.17]	0.19	12.52	0.01	68%	1.26 (0.21)	
	ACE	2	105	-0.38 [-1.06, 0.29]	0.15	2.76	0.10	64%	1.12 (0.26)	
Chao1										
Age (years)	<70	5	362	-0.48 [-0.90, -0.06]	0.15	12.49	0.01	68%	2.26 (0.02)	0.02 (0.89)
	≥70	6	431	-0.45 [-0.70, -0.19]	0.03	7.14	0.21	30%	3.41 (0.0006)	
Gender	Both	10	750	-0.42 [-0.65, -0.18]	0.07	18.86	0.03	52%	3.46 (0.0005)	1.01 (0.32)
	Female	1	43	-0.77 [-1.41, -0.13]	Not applicable				2.34 (0.02)	
BMI (kg/m²)	≥24.5	4	244	-0.21 [-0.60, 0.18]	0.07	5.66	0.13	47%	1.07 (0.28)	2.13 (0.14)
	<24.5	6	480	-0.57 [-0.85, -0.29]	0.05	9.02	0.11	45%	3.94 (< 0.0001)	
Region	Eastern country	8	592	-0.58 [-0.79, -0.36]	0.02	9.46	0.22	26%	5.29 (< 0.0001)	8.41 (0.004)
	Western country	3	201	-0.04 [-0.33, 0.26]	0.00	1.55	0.46	0%	0.24 (0.81)	
Sarcopenia diagnostics criteria	AWGS	5	255	-0.59 [-1.00, -0.18]	0.12	8.57	0.07	53%	2.83 (0.005)	0.99 (0.61)
	EWGSOP	2	308	-0.26 [-0.77, 0.25]	0.11	4.46	0.03	78%	1.01 (0.31)	
	Other	4	230	-0.42 [-0.80, -0.04]	0.06	5.35	0.15	44%	2.19 (0.03)	
Muscle mass measurement	BIA	5	296	-0.70 [-1.05, -0.36]	0.07	7.06	0.13	43%	3,96 (< 0.0001)	5.49 (0.06)
	DXA	3	120	-0.12 [-0.49, 0.26]	0.00	1.40	0.50	0%	0.60 (0.55)	
	CT/MRI	2	185	-0.23 [-0.76, 0.30]	0.09	2.81	0.09	64%	0.86 (0.39)	
Nutrition status	Malnutrition /malnutrition risk	2	119	-0.78 [-1.17, -0.38]	0.00	0.00	0.98	0%	3.85 (0.0001)	6.06 (0.01)
	Healthy	1	50	0.15 [-0.47, 0.76]	Not applicable				0.46 (0.64)	
Observed species/OTUs										
Age (years)	<70	3	184	-0.79 [-1.19, -0.40]	0.03	2.55	0.28	22%	3.97 (< 0.00001)	0.85 (0.36)
	≥70	2	268	-0.58 [-0.83, -0.33]	0.00	0.86	0.35	0%	4.51 (< 0.00001)	
Sarcopenia diagnostics criteria	AWGS	2	115	-0.87 [-1.34, -0.41]	0.00	0.08	0.78	0%	3.68 (0.0002)	1.83 (0.40)
	EWGSOP	1	192	-0.50 [-0.79, -0.21]	Not applicable				3.40 (0.0007)	
	Other	2	145	-0.66 [-1.01, -0.30]	0.00	0.55	0.46	0%	3.63 (0.0003)	
Muscle mass measurement	BIA	3	191	-0.83 [-1.17, -0.49]	0.00	0.14	0.93	0%	4.80 (< 0.00001)	1.02 (0.31)
	CT/MRI	1	69	-0.52 [-1.02, -0.02]	Not applicable				2.05 (0.04)	
Shannon										
Age (years)	<70	7	1980	-0.24 [-0.59, 0.12]	0.17	35.83	<0.00001	83%	1.30 (0.19)	1.08 (0.30)
	≥70	3	156	-0.49 [-0.83, -0.16]	0.00	0.56	0.76	0%	2.88 (0.004)	
Gender	Both	9	2091	-0.31 [-0.62, 0.01]	0.17	43.46	<0.00001	82%	1.88 (0.06)	0.00 (0.99)
	Female	1	45	-0.30 [-0.91, 0.32]	Not applicable				0.95 (0.34)	
BMI (kg/m²)	<24.5	6	1679	-0.49 [-1.04, 0.05]	0.38	39.51	<0.00001	87%	1.78 (0.07)	3.48 (0.06)
	≥24.5	3	388	0.06 [-0.14, 0.26]	0.00	1.46	0.48	0%	0.55 (0.58)	
Sarcopenia diagnostics criteria	AWGS	8	1991	-0.25 [-0.59, 0.08]	0.17	36.83	<0.00001	81%	1.48 (0.14)	0.95 (0.33)
	Other	2	145	-0.49 [-0.84, -0.14]	0.00	0.22	0.64	0%	2.77 (0.006)	
Muscle mass measurement	BIA	7	1987	-0.06 [-0.30, 0.17]	0.05	15.37	0.02	61%	0.53 (0.60)	4.46 (0.11)
	DXA	2	80	-1.18 [-2.37, 0.01]	0.61	5.66	0.02	82%	1.94 (0.05)	
	CT/MRI	1	69	-0.41 [-0.91, 0.09]	Not applicable			1.62 (0.11)	
Nutrition status	Malnutrition /malnutrition risk	3	1538	-0.20 [-0.69, 0.29]	0.14	8.29	0.02	76%	0.80 (0.42)	12.76 (0.0004)
	Healthy	1	45	-1.79 [-2.52, -1.07]	Not applicable				4.86 (< 0.00001)	
Simpson										
Age (years)	<70	3	166	-0.22 [-0.83, 0.39]	0.21	7.36	0.03	73%	0.69 (0.49)	0.16 (0.69)
	≥70	2	81	-0.45 [-1.44, 0.53]	0.39	4.43	0.04	77%	0.90 (0.37)	
Gender	Both	4	201	-0.39 [-0.97, 0.19]	0.26	11.73	0.008	74%	1.32 (0.19)	0.96 (0.33)
	Female	1	46	0.03 [-0.57, 0.63]	Not applicable				0.09 (0.93)	
BMI (kg/m²)	≥24.5	2	106	0.01 [-0.38, 0.40]	0.00	0.00	0.94	0%	0.06 (0.95)	1.50 (0.22)
	<24.5	3	141	-0.55 [-1.35, 0.26]	0.40	10.08	0.006	80%	1.33 (0.18)	

##### 3.4.2.1 Chao1 index

In **Table 5**, a significant reduction in the Chao1 index was both observed in aged < 70 years (SMD: -0.48, 95% CI: -0.90 to -0.06, p: 0.02) or ≥ 70 years (SMD: -0.45, 95% CI: -0.70 to -0.19, p: 0.0006), both genders (SMD: -0.42, 95% CI: -0.65 to -0.18, p: 0.0005) or females (SMD: -0.77, 95% CI: -1.41 to -0.13, p: 0.02), with AWGS (SMD: -0.59, 95% CI: -1.00 to -0.18, p: 0.005) or other sarcopenia criteria (SMD: -0.42, 95% CI: -0.80 to -0.04, p: 0.03)

The Chao1 index also significantly decreased in participants with a BMI < 24.5 (SMD: -0.57, 95% CI: -0.85 to -0.29, p < 0.0001), originating from Eastern countries (SMD: -0.58, 95% CI: -0.79 to -0.36, p < 0.0001), utilizing BIA for muscle mass measurement (SMD: -0.70, 95% CI: -1.05 to -0.36, p < 0.0001), at risk of malnutrition or suffering from malnutrition (SMD: -0.78, 95% CI: -1.17 to -0.38, p: 0.0001).

##### 3.4.2.2 Observed species/OTUs

In **Table 5**, the Observed species/OTUs significantly decreased in participants in aged < 70 years (SMD: -0.79, 95% CI: -1.19 to -0.40, p: < 0.00001) or ≥ 70 years (SMD: -0.58, 95% CI: -0.83 to -0.33, p: < 0.00001), with AWGS (SMD: -0.87, 95% CI: -1.34 to -0.41, p: 0.0002), EWGSOP (SMD: -0.50, 95% CI: -0.79 to -0.21, p: 0.0007) or other sarcopenia criteria (SMD: -0.66, 95% CI: -1.01 to -0.30, p: 0.0003), utilizing BIA (SMD: -0.83, 95% CI: -1.17 to -0.49, p < 0.00001) or CT/MRI (SMD: -0.52, 95% CI: -1.02 to -0.02, p: 0.04) for muscle mass measurement.

##### 3.4.2.3 Shannon index

In **Table 5**, the Shannon index significantly decreased in participants in aged ≥ 70 years (SMD: -0.49, 95% CI: -0.83 to -0.16, p: 0.004), with other sarcopenia criteria (SMD: -0.49, 95% CI: -0.84 to -0.14, p: 0.006), and in healthy status (SMD: -1.79, 95% CI: -2.52 to -1.07, p < 0.00001).

##### 3.4.2.4 Simpson index

In **Table 5**, the Simpson index showed no significant differences between the sarcopenia and non-sarcopenia groups, regardless of age, sex, or body mass index subgroups.

#### 3.4.3 Risk of bias

According to the funnel plot in **Supplementary Figure S1** and Egger’s regression test in **Supplementary Table S3**, there was no publication bias in the Chao 1 index (p: 0.559) or the Observed species/OTUs (p: 0.067). However, publication bias was detected in the Shannon index (p: 0.018) and the Simpson index (p: 0.039).

#### 3.4.4 Sensitivity analysis

The sensitivity analysis revealed that the pooled effect size for all α-diversity indicators remained within the 95% CI after the exclusion of any individual study. This finding indicates the stability of the α-diversity indicators (**Supplementary Figure S2**). In addition, the sensitivity analysis revealed that different studies had the greatest influence on various α-diversity indices. Specifically, Aliwa et al. had the greatest impact on Chao1 (Aliwa et al., 2023), Wu et al. on Observed species/OTUs (Wu et al., 2022), Lee et al. on Shannon index (Lee et al., 2023), and Peng et al. on the Shannon index (Peng et al., 2023).

### 3.5 Quantitative synthesis of β-diversity

14 studies employed various methods to assess β-diversity. Six studies used the Bray-Curtis similarity. Six studies used the Bray-Curtis similarity (Han et al., 2022; Lee et al., 2022; Wang et al., 2022c; Peng et al., 2023; Zhang et al., 2023, 2024), two used the Unweighted UniFrac distances (Kang et al., 2021; Lee et al., 2023), one used the Weighted UniFrac distances (Ponziani et al., 2021), and two used the PLS-DA (Kang et al., 2021; Zhang et al., 2024), all of which demonstrated significant differences in β-diversity between the sarcopenia and non-sarcopenia groups. In contrast, the remaining studies showed no significant differences between the two groups (Ticinesi et al., 2020; Yamamoto et al., 2022; Aliwa et al., 2023; Liu et al., 2023; Yan et al., 2023) (**Table 6**).

**Table 6:** Summary of β diversity assessments in the included studies.

Study (author, year)	Beta diversity	Findings	Statistic value
Aliwa et al., 2023	Bray-Curtis dissimilamty using PCoA	No significant difference in gut microbial composition between Cirrhosis with S and Cirrhosis without S	NR
Han et al., 2022	Bray-Curtis dissimilamty using PCoA	A significant difference in gut microbial composition between NM and LM	p = 0.037
Kang et al., 2021	Unweighted UniFrac distances using PCoA	A significant difference in gut microbial composition between S and NS	p = 0.08
	PLS-DA	A significant difference in gut microbial composition between S and NS	p = 0.0001
Lee et al., 2023	Unweighted UniFrac distances	A significant difference in gut microbial composition between Cirrhosis with S and NS	p = 0.001
Lee et al., 2022	Bray-Curtis dissimilarity using NMDS based on species abundance	A significant difference in gut microbial composition between S and NS	p = 0.049
Liu et al., 2023	Bray-Curtis dissimilamty using PCoA based on genus abundance	No significant difference in gut microbial composition between S and NS	NR
Peng et al., 2023	Bray-Curtis dissimilamty using PCoA based on OTUs abundance	A significant difference in gut microbial composition between SHF and NS	p = 0.002
Ponziani et al., 2021	Weighted UniFrac distances using PCoA	A significant difference in gut microbial composition between S and NS	p = 0.03
Ticinesi et al., 2020	Bray-Curtis dissimilamty using PCoA based on species abundance	No significant difference in gut microbial composition between S and NS	p = 0.36
Wang et al., 2022c	Bray-Curtis dissimilamty using PCoA based on genus abundance	A significant difference in gut microbial composition between S and NS	p = 0.042
	Bray-Curtis dissimilamty using PCoA based on species abundance	A significant difference in gut microbial composition between S and NS	p = 0.02
Yamamoto et al., 2022	Bray-Curtis dissimilamty	No significant difference in gut microbial composition between N-SMI and L-SMI	NR
Yan et al., 2023	Bray-Curtis dissimilamty using PCoA based on OTUs abundance	No significant difference in gut microbial composition between S and NS	NR
Zhang et al., 2023	Bray-Curtis dissimilamty using PCoA based on ASVs abundance	A significant difference in gut microbial composition between S and NS	p = 0.001
Zhang et al., 2024	PLS-DA	A significant difference in gut microbial composition between S and NS	NR
	Bray-Curtis dissimilamty using PCoA based on OTUs abundance	A significant difference in gut microbial composition between S and NS	p = 0.015

NS, Non-sarcopenia; S, Sarcopenia; NM, Normal muscle mass; LM, Low muscle mass; SHF, Heart failure with sarcopenia; N-SMI, Normal skeletal muscle mass index; L-SMI, Low skeletal muscle mass index; OTUs, Operational Taxonomic Units; ASVs, amplicon sequence variants; PCoA, Principal Coordinate Analysis; NMDS, Non-metric multi-dimensional scaling; PLS-DA, Partial Least squares Discriminant Analysis; NR, Not reported.

### 3.6 Quantitative/qualitative synthesis of relative abundance

**Figure 3:**
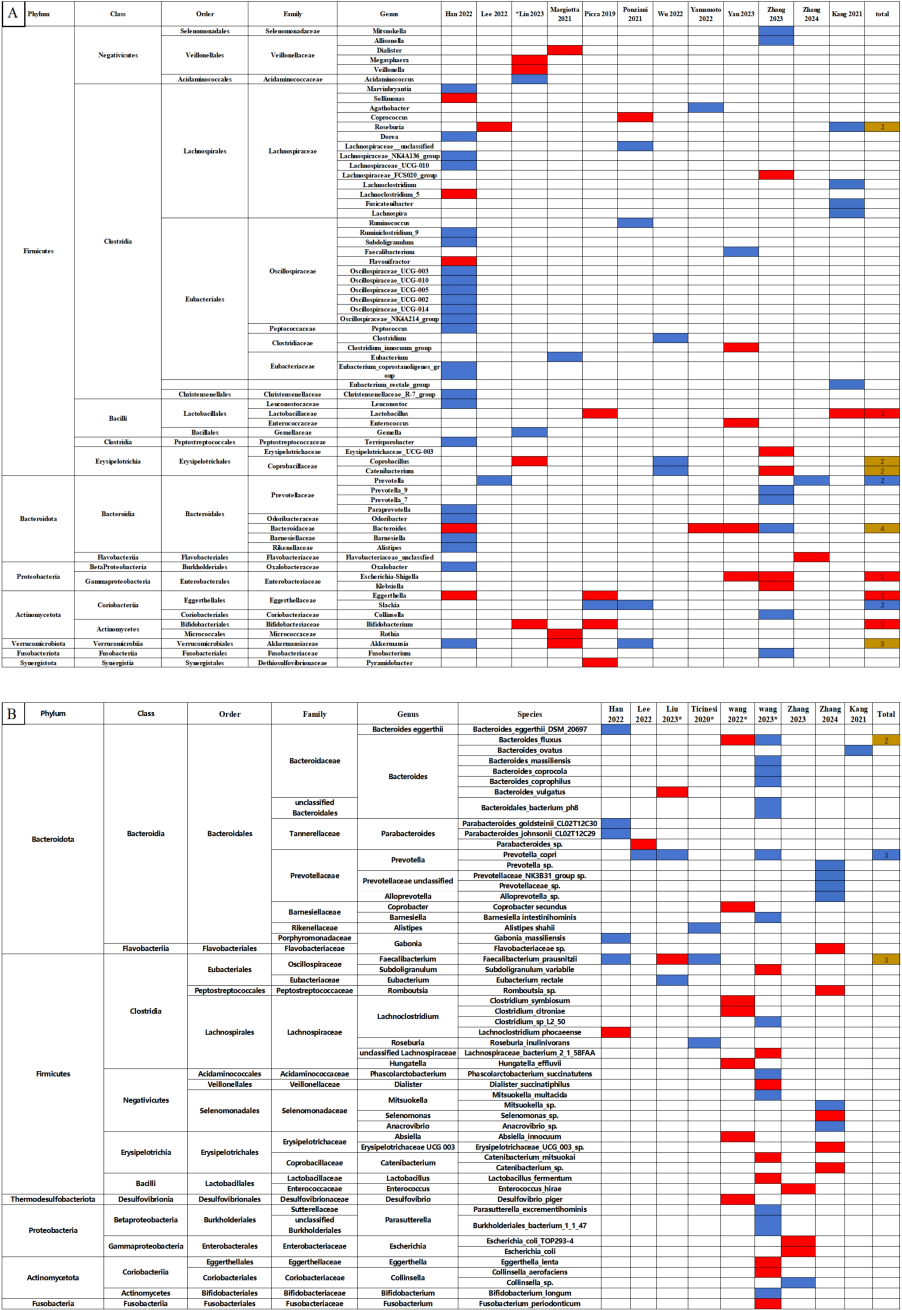
Changes in the relative abundance of microbes in the included studies. **(A)** Genus level. **(B)** Species level. The red and blue grids indicate statistically significant increases and decreases in taxa with sarcopenia, respectively. In the total row, the numerical value represents the number of studies reporting significant changes in the taxa. Red grids indicate a significant increase, blue grids a significant decrease, and brown grids indicate both increases and decreases in sarcopenia. *Represents studies using shotgun metagenomic sequencing. Each microbe is labeled with the level to which it belongs.

Quantitative comparisons of relative abundance at phyla, class, order, family, genus, and species levels between the sarcopenia and non-sarcopenia groups are presented in **Supplementary Table S2**. The qualitative analysis of relative abundance at the genus and species levels were shown in **Figure 3**, while qualitative analyses of the phylum, class, order and family levels were shown in **Supplementary Figure S3**.

In **Figure 3**, we observed that the following microbes which showed significant differences in two and more studies: The Lactobacillus, Bifidobacterium, Escherichia-Shigella and Eggerthella at the genus level significantly increased in the sarcopenia groups compared to non-sarcopenia groups. In contrast, there were significant reductions in Prevotella and Slackia at the genus level, and Prevotella copri at the species level. The relative abundance of the Roseburia, Coprobacillus, Catenibacterium, Bacteroides and Akkermansia genera, and the Bacteroides fluxus and Faecalibacterium prausnitzii species presented inconsistent results across the studies.

### 3.7 Correlation between gut microbiota and sarcopenia parameters

#### 3.7.1 Gut microbiota with a positive “Final relevance” to muscle parameters

**Figure 4:**
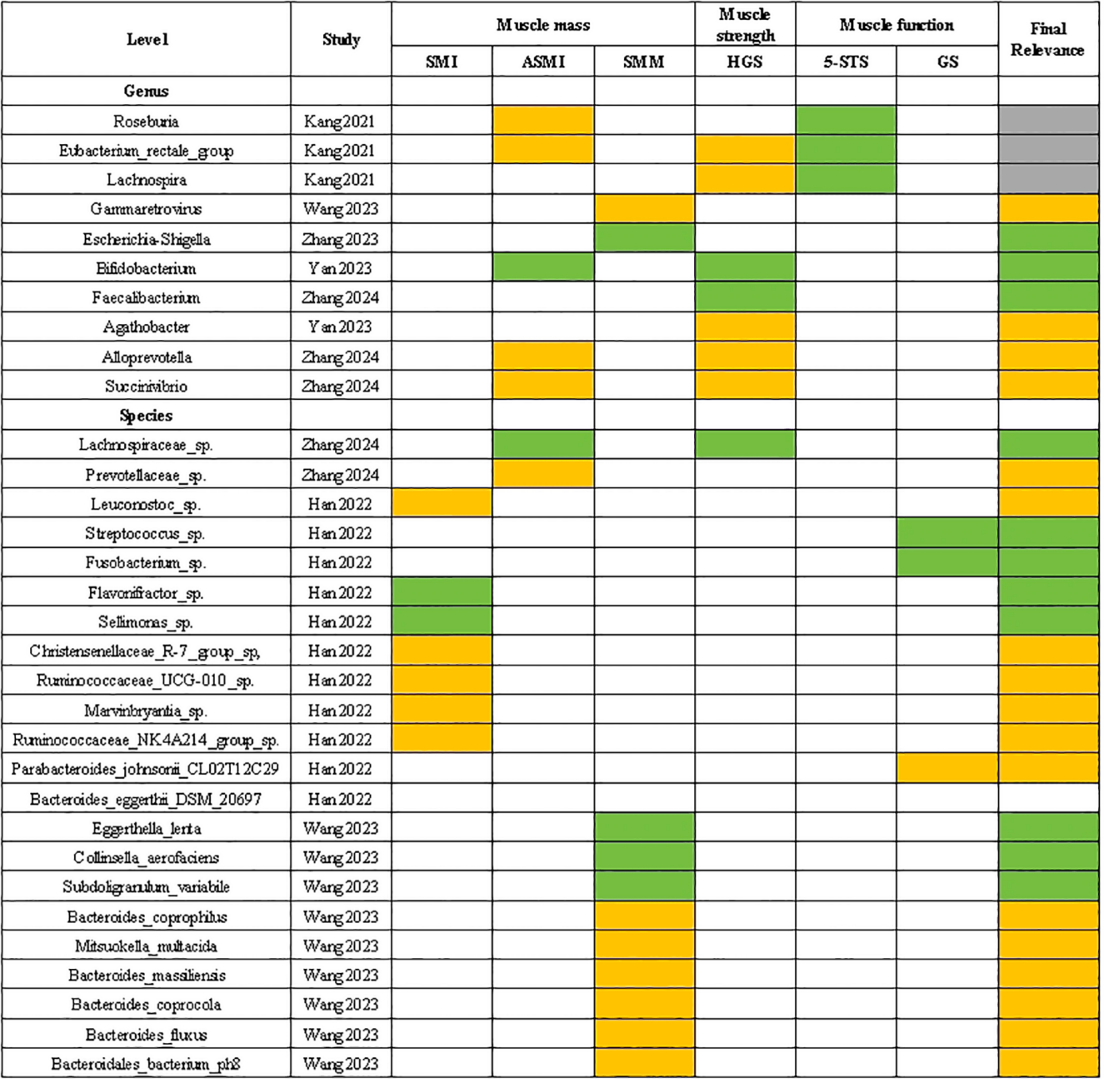
Correlation between gut microbiota and sarcopenia parameters. The green grids: negative and significant correlation; The orange grids: positive and significant correlation; SMI, skeletal muscle index; ASMI, appendicular skeletal muscle mass index; SMM, skeletal muscle mass; HGS, handgrip strength; 5-STS, five times sit to stand test; GS, gait speed.

In **Figure 4**, gut microbiota that showed a positive ‘Final relevance’ to muscle parameters include the Gammaretrovirus, Agathobacter, Alloprevotella, Succinivibrio at the genus level, and the Prevotellaceae sp., Leuconostoc sp., Christensenellaceae R-7 group sp, Ruminococcaceae UCG-010 sp., Marvinbryantia sp., Ruminococcaceae NK4A214 group sp., Parabacteroides johnsonii CL02T12C29, Bacteroides eggerthii DSM 20697, Bacteroides coprophilus, Mitsuokella multacida, Bacteroides massiliensis, Bacteroides coprocola, Bacteroides fluxus and Bacteroidales bacterium ph8 at the species level.

#### 3.7.2 Gut microbiota with a negative “Final relevance” to muscle parameters

In **Figure 4**, gut microbiota that showed a negative ‘Final relevance’ to muscle parameters include the Escherichia-Shigella. Bifidobacterium and Faecalibacterium at the genus level, and the Lachnospiraceae sp., Streptococcus sp., Fusobacterium sp., Flavonifractor sp., Sellimonas sp., Eggerthella lenta, Collinsella aerofaciens and Subdoligranulum variabile at the species level.

#### 3.7.3 Gut microbiota with an uncertain “Final relevance” to muscle parameters

In **Figure 4**, gut microbiota that showed an uncertain ‘Final relevance’ to muscle parameters include the Roseburia. Eubacterium rectale group and Lachnospira at the species level.

### 3.8 Summaries of gut microbiota with potential relevance to sarcopenia

#### 3.8.1 Gut microbiota with potential and negative relevance to sarcopenia

Gut microbiota that have a potentially negative relevance with sarcopenia include the Prevotella, Slackia, Agathobacter and Alloprevotella at the genus level, and the Prevotella copri, Prevotellaceae sp., Parabacteroides johnsonii CL02T12C29, Bacteroides coprophilus, Bacteroides massiliensis, Bacteroides coprocola and Bacteroidales bacterium ph8 species and Mitsuokella multacidaat the species level.

#### 3.8.2 Gut microbiota with potential and positive relevance to sarcopenia

Gut microbiota that have a potentially positive relevance with sarcopenia include the Lactobacillus, Escherichia-Shigella, Eggerthella, Bifidobacterium at the genus level, and the Eggerthella lenta, Collinsella aerofaciens, Subdoligranulum variabile at the species level.

#### 3.8.3 Gut microbiota with potential but unclear relevance to sarcopenia

Gut microbiota that have a potentially but unclear relevance with sarcopenia include the Roseburia, Coprobacillus, Catenibacterium, Lachnospira, Bacteroides, Akkermansia and Eubacterium rectale group at the genus level, and Bacteroides fluxus and Faecalibacterium prausnitzii at the species level.

## 4 Discussion

Our study systematically compare the diversity and composition of gut microbiota between older people with and without sarcopenia. The main findings of this study include the following: Firstly, older people with sarcopenia showed a significant reduction in α-diversity, probably predominantly due to diminished richness rather than evenness. Secondly, the findings of β-diversity varied across included studies. Thirdly, our study identified certain gut microbiota which had a potential and negative correlation with sarcopenia, such as Prevotella, Slackia, Agathobacter, Alloprevotella, Prevotella copri, Prevotellaceae sp., Bacteroides coprophilus, Mitsuokella multacida, Bacteroides massiliensis, Bacteroides coprocola, suggesting their potential probiotic role for sarcopenia. In addition, we also identified conditionally pathogenic bacteria with a potential and positive association with sarcopenia like Escherichia-Shigella, Eggerthella, Eggerthella lenta and Collinsella aerofaciens, implying that their targeted suppression may be beneficial in sarcopenia treatment.

Numerous studies align with the outcomes of our meta-analysis, consistently reporting a significant reduction in α-diversity among individuals with sarcopenia (Wang et al., 2022c; Lou et al., 2024). α-diversity in the gut microbiota is a key indicator of host health, with higher diversity typically associated with a stable gut ecosystem. Low α-diversity in older people with sarcopenia likely indicates gut dysbiosis and an impaired state of health (Lloyd-Price et al., 2016). In this study, we observed that the Chao 1 index and observed species/OTUs, indicators of species richness, showed significant reductions in older people with sarcopenia, suggesting a loss of certain gut microbial species. The Shannon and Simpson indices, which account for both richness and evenness, provide additional insights (Lozupone et al., 2012). A decline in the Shannon index suggesteds that reduced α-diversity may be accompanied by decreased evenness, which may be due to an increase in pathogenic microbes or a decrease in beneficial microbes in sarcopenia (Zhang et al., 2022b). However, the constancy of the Simpson index may indicate that the overall evenness of the gut microbial community has not changed significantly. These findings suggest that sarcopenia is closely associated with a significant reduction in α-diversity of the gut microbiota, with a primary reduction in species richness and a less pronounced impact on evenness.

Subgroup analyses consistently revealed an overall decline in α-diversity among older people with sarcopenia, though significant heterogeneity existed between subgroups. The reduction in Chao1 index and OTUs across age groups (<70 and ≥70 years) suggested that the age-related effects on α-diversity stem from multiple factors rather than a single age threshold. Specifically, aging may lead to immune system dysregulation, resulting in chronic low-grade inflammation (Franceschi and Campisi, 2014), which negatively impacts both muscle function (Nardone et al., 2021) and gut microbiota (Evenepoel et al., 2023). Furthermore, the cumulative effects of comorbidities, such as diabetes (Yang et al., 2021) and cardiovascular diseases (Chen et al., 2023), may exacerbate these changes by altering metabolism and promoting inflammation, ultimately affecting α-diversity. Notably, a marked decrease in the Chao1 index was observed in individuals with a BMI below 24.5 or at risk of malnutrition, suggesting a potential link between low BMI, malnutrition, and reduced gut microbial diversity (Lozupone et al., 2012; Sergeev et al., 2020; Iddrisu et al., 2021). Consistent with these findings, Farsijani et al. conducted a cross-sectional analysis of 775 older men from the Osteoporotic Fractures in Men Study (MrOS), which showed that higher protein intake, whether from animal or vegetable sources, was associated with increased gut microbiome diversity (Farsijani et al., 2023). Similarly, Dominianni et al. highlighted that both BMI and dietary fiber intake contribute to shaping the human gut microbiome (Dominianni et al., 2015). Collectively, these studies underscore the critical role of nutritional interventions in improving both gut microbiota and sarcopenia (Chen et al., 2020). Moreover, subgroup analyses revealed a significant impact of geographic region on α-diversity. An observational study exploring the significant differences in gut microbiota composition between older women from island and inland areas supports this view (Shin et al., 2016). The study found that the subjects from the island area exhibited higher gut microbial diversity, with notable differences in microbial community composition between the two groups. Specifically, Catenibacterium was enriched in the island group, while Butyricimonas was enriched in the inland group. These differences were associated with environmental factors such as diet and physical activity. Additionally, subgroup analyses indicated that the diagnostic criteria for sarcopenia and methods for measuring muscle mass significantly influenced α-diversity.

In **Table 6**, β-diversity of the 14 studies showed significant inconsistencies. Similar to α-diversity, differences in β-diversity across studies may stem from the metrics to measure β-diversity, the statistical methods applied, the participants’ characteristics and study design. For example, there are significant differences in gut microbiota composition between East Asian and Western populations. Specifically, East Asian populations generally exhibit a Prevotella-dominated enterotype, while Western populations are predominantly Bacteroides-dominated. These differences may arise from geographic-specific factors such as dietary patterns, host genetic backgrounds, and early microbial colonization patterns (Wu et al., 2011). In addition, different sarcopenia diagnostic criteria place varying emphasis on participant inclusion. Specifically, the AWGS criteria may include more individuals with mildly reduced muscle mass but relatively preserved function (Chen et al., 2020), while the EWGSOP criteria tend to include individuals with more severe muscle function impairment (Cruz-Jentoft et al., 2010). The severity of sarcopenia, along with associated chronic low-grade inflammation and alterations in immune system function, can influence the composition of the gut microbiota. In fact, the majority of the studies included in our review analyzed β-diversity without conducting subgroup analyses based on participants’ characteristics or study design, which may limit the identification of confounding factors that could affect β-diversity. Of particular note, the small sample size of the study by Ticinesi et al (Ticinesi et al., 2020), which included only five patients in the sarcopenia group, may not have been sufficient to accurately assess inter-individual microbial diversity, and thus the reliability of the results is limited.

According to the criteria established by this study, we identified gut microbiota with potential and negative relevance to sarcopenia: the Prevotella, Slackia, Agathobacter and Alloprevotella at the genus level, and the Prevotella copri, Prevotellaceae sp., Parabacteroides johnsonii CL02T12C29, Bacteroides coprophilus, Bacteroides massiliensis, Bacteroides coprocola, Bacteroidales bacterium ph8 and Mitsuokella multacida at the species level. The Prevotella (Trautmann et al., 2020), Agathobacter (Scott et al., 2014), Alloprevotella (Han et al., 2024) and Mitsuokella multacida (De Vos et al., 2024) were recognized as producers of SCFAs and previous research suggests that Slackia also reacts positively to SCFAs (Jin et al., 2021). SCFAs, key metabolic products of the gut microbiota, primarily include butyrate, acetate, and propionate (Tramontano et al., 2018). SCFAs play crucial roles in regulating muscle cell function through various mechanisms, such as reducing inflammation (Vinolo et al., 2011), enhancing mitochondrial activity (Saint-Georges-Chaumet and Edeas, 2016), stimulating protein synthesis (Lin et al., 2017), and improving energy supply (Yang et al., 2018). Besten et al. demonstrated that SCFAs regulate skeletal muscle by increasing the AMP/ATP ratio or activating AMPK via the FFAR2-leptin pathway (den Besten et al., 2013). Additionally, SCFAs promote the expression of genes involved in muscle protein synthesis through the mTOR/IGF-1 pathway (Hay and Sonenberg, 2004; Grosicki et al., 2018). Further research has shown that SCFAs raise GLP-1 concentrations in the blood, which has been shown to enhance glucose-stimulated insulin secretion (Delzenne et al., 2007). Therefore, a reduction in SCFAs production is associated with insulin resistance and the accumulation of fatty acids in muscle cells (Gao et al., 2009), leading to a decline in muscle mass and exacerbating insulin resistance, ultimately contributing to the development of sarcopenia (Poggiogalle et al., 2019; Sachs et al., 2019). Consequently, the observed potential and negative correlation between SCFA-producing bacteria and sarcopenia suggested that these bacteria may play a protective role against muscle atrophy, and these bacteria may be considered as potential probiotic candidates for the treatment of sarcopenia.

Although direct evidence of SCFAs production by Prevotella copri and Prevotellaceae sp. was lacking, their taxonomic affinity to Prevotella suggested that they may also contribute positively to muscle health (Prasoodanan et al., 2021). Additionally, within the Bacteroides genus, several species have shown a potential and negative correlation with sarcopenia. For instance, Bacteroides massiliensis was known for its production of SCFAs (Sokol et al., 2021). B. coprophilus was found to be inversely associated with the pro-inflammatory cytokines (Yan et al., 2019), and inflammatory reactions play a significant role in the development of sarcopenia (Wumaer et al., 2022), suggesting that it may have a positive impact on the treatment of sarcopenia through anti-inflammatory effects. Furthermore, the reduction of Bacteroides coprocola in patients with polycystic ovary syndrome (PCOS) implied a potential link between the microbe and poor health (Yang et al., 2022b), but the specific mechanisms by which this bacterium might be related to sarcopenia require further investigation.

At the same time, we also summarized gut microbiota with potential and positive relevance to sarcopenia: the Lactobacillus, Bifidobacterium, Escherichia-Shigella, Eggerthella at the genus level, and the Eggerthella lenta, Collinsella aerofaciens, Subdoligranulum variabile at the species level.

There was a significant increase in certain conditionally pathogenic bacteria in sarcopenia. For instance, the Escherichia-Shigella, merged into one genus in the 16S SILVA database (Lu and Salzberg, 2020), may promote inflammation and amino acid metabolism abnormalities by increasing the permeability of the intestinal barrier, thereby disrupting the normal metabolism of muscles (Sartor, 2008; Wang et al., 2021; Yang et al., 2022a). The genus Eggerthella contains many pathogenic species, including Eggerthella lenta, which is associated with gastrointestinal diseases (Krogius-Kurikka et al., 2009; Würdemann et al., 2009; Thota et al., 2011). Eggerthella lenta was associated with systemic inflammation and insulin resistance (Koh et al., 2018; Vieira-Silva et al., 2020). The uremic toxins and inflammatory mediators produced by this bacteria may lead to a loss of muscle mass (Popkov et al., 2022; Hung et al., 2023). Additionally, an increase in the Eggerthella genus among the older people with frailty indicated a potential role in the progression of sarcopenia (Jackson et al., 2016). Collinsella aerofaciens was abundant in inflammatory diseases (Malinen et al., 2010; Joossens et al., 2011; Walker et al., 2011), metabolic syndrome and obesity (Gomez-Arango et al., 2018; Gallardo-Becerra et al., 2020), so we speculated its increase may be associated with the host’s metabolic abnormalities and inflammatory status, both of which are key factors in the development of sarcopenia.

Conditional pathogens can trigger systemic inflammation (La Ragione et al., 2004) and interfere with the metabolic homeostasis through the production of harmful metabolites such as lipopolysaccharide (LPS), which further affects muscle protein synthesis and catabolic processes, ultimately leading to sarcopenia (Sawicka et al., 2018; Liu et al., 2021b). Specifically, TNF-α activates the NF-κB pathway, which prevents myogenic differentiation, leading to muscle atrophy (Langen et al., 2001). Elevated levels of IL-6 are associated with insulin resistance (Rehman et al., 2017) accelerating muscle wasting. LPS-induced activation of TLR4 and p38 MAPK leads to C2C12 muscle atrophy by enhancing autophagy and increasing the expression of ubiquitin ligases (Doyle et al., 2011). Moreover, gut microbiota dysbiosis may further promote the growth of conditional pathogens, creating a vicious cycle that exacerbates the decline in muscle mass and function (Huang et al., 2017; Chen et al., 2022). Therefore, targeting the regulation of gut microbiota, especially inhibiting the proliferation of these conditionally pathogenic bacteria, could serve as an important strategy for the prevention and treatment of sarcopenia.

In addition, our research has revealed an interesting phenomenon: some bacteria typically associated with health benefits, such as Lactobacillus (Zhai et al., 2019), Bifidobacterium (Xu et al., 2021), and Subdoligranulum variabile (Van Hul et al., 2020), have been found to increase in older people with sarcopenia. This phenomenon may be explained by two mechanisms. Firstly, these bacteria were capable of benefiting muscle through the production of SCFAs or other pathways (Louis and Flint, 2009; 2017; Wang et al., 2022a; Xiao et al., 2022). Thus, their increase in sarcopenia may represent a compensatory response, aimed at combating the chronic inflammation and metabolic dysregulation associated with sarcopenia. Additionally, Bifidobacterium facilitated the absorption and utilization of essential nutrients like vitamin D and minerals (Montazeri-Najafabady et al., 2019), potentially improving the nutrient malabsorption in sarcopenic patients (Nishida et al., 2020). Secondly, although these genera generally exhibited beneficial effects, certain species within them may demonstrate pathogenic potential under specific conditions (Costa et al., 2020). For instance, some Lactobacillus species have been observed to increase under inflammatory conditions (Salminen et al., 2006; Liu et al., 2013), and their treatment in mice has led to a significant upregulation of inflammatory cytokines (Roh et al., 2018), suggesting a complex relationship between these bacteria and sarcopenia.

The Roseburia, Coprobacillus, Catenibacterium, Lachnospira, Bacteroides, Akkermansia, and Eubacterium rectale group genera, as well as Bacteroides fluxus and the Faecalibacterium prausnitzii species, were categorized as gut microbiota with potential but unclear relevance to sarcopenia. Except for Bacteroides fluxus which was pathogenic (Li et al., 2022), the remaining microbes were typically beneficial for their direct or indirect favorable role in producing SCFAs (Kageyama and Benno, 2000; Duncan and Flint, 2008; Sokol et al., 2008; Reichardt et al., 2014; Barrett et al., 2018; Ding et al., 2019; Leyva-Diaz et al., 2021; Pardesi et al., 2022). Consequently, we anticipated observing reduced abundances of these bacteria in sarcopenia. The growth of these microbes in some studies might be ascribed to compensatory mechanisms, as well as differences in species levels within genera (Costa et al., 2020).

**Figure 5:**
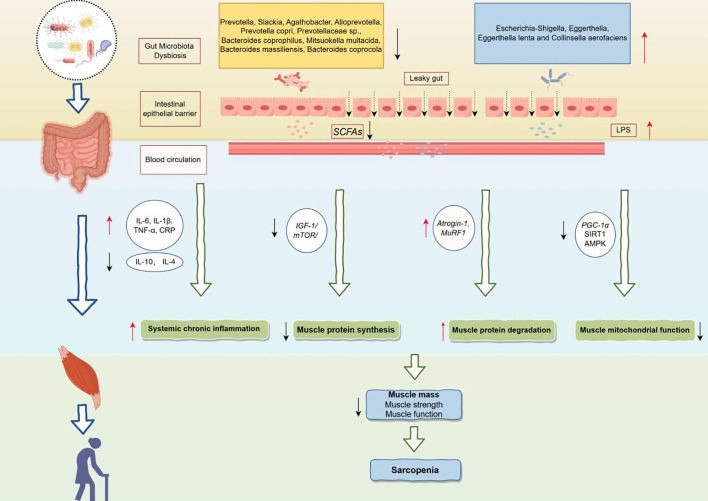
Potential mechanisms of the gut microbiota leading to sarcopenia. IL-6, Interleukin-6; IL-1β, Interleukin-1 beta; TNF-α, Tumor necrosis factor alpha; CRP, C-reactive protein; IL-10, Interleukin-10; IL-4, Interleukin-4; IGF-1, Insulin-like growth factor 1; mTOR, Mechanistic target of rapamycin; Atrogin-1, F-box only protein 32; MuRF1, Muscle RING finger protein 1; PGC-1α, PPAR-gamma coactivator 1-alpha; SIRT1, Sirtuin 1; AMPK, AMP-activated protein kinase.

**Figure 5** showed the potential mechanisms by which the gut microbiota above contribute to sarcopenia. Gut dysbiosis, characterized by an overgrowth of pathogenic bacteria and a deficiency of beneficial bacteria, can compromise the intestinal barrier and increase intestinal permeability (Ling et al., 2022). This gut microbiota imbalance leads to a decrease in beneficial metabolites such as SCFAs and an increase in harmful metabolites such as LPS (Sun and Shen, 2018). Specifically, SCFAs provide approximately 10% of the daily energy required by the human body (Marchesi et al., 2016) and play a crucial role in regulating cell growth and differentiation (Rosser et al., 2020). SCFAs have been shown to influence skeletal muscle by modulating myelocyte function and protein synthesis pathways, increasing ATP production, improving insulin sensitivity, promoting fat oxidation, limiting muscle fat deposition, and reducing inflammation (Lv et al., 2021). Walsh et al. demonstrated that supplementation with butyrate in mice could inhibit histone β-hydroxybutyrylase activity and provide protection against hindlimb muscle atrophy (Walsh et al., 2015).

Conversely, the increase in LPS resulting from gut microbiota dysbiosis activates pro-inflammatory pathways, leading to elevated levels of pro-inflammatory cytokines such as interleukin-6 (IL-6), interleukin-1β (IL-1β), and tumor necrosis factor-α (TNF-α) in the blood, which may induce systemic chronic inflammation (Liu et al., 2021a). In addition, gut dysbiosis may inhibit muscle protein synthesis by disrupting the Insulin-like growth factor 1/Mechanistic target of rapamycin (IGF-1/mTOR) signaling pathway (Dukes et al., 2015; de Marco Castro et al., 2021), which in turn affects muscle growth and repair processes. Meanwhile, upregulation of F-box only protein 32 (Atrogin-1) and Muscle RING finger protein 1 (MuRF1) promoted muscle protein degradation and exacerbated the loss of muscle mass (Kang et al., 2024). Furthermore, gut dysbiosis may inhibit mitochondrial function by down-regulating key metabolic regulators, such as PPAR-gamma coactivator 1-alpha (PGC-1α), Sirtuin 1 (SIRT1) and AMP-activated protein kinase (AMPK), affecting energy metabolism and function of muscle cells (Zhang et al., 2022b).

In summary, gut microbiota dysbiosis, through metabolic disturbances, chronic inflammation, and an imbalance in protein synthesis and degradation, ultimately leads to a decline in skeletal muscle mass, strength, and function, thereby contributing to sarcopenia. Moreover, studies have shown that additional supplementation with probiotics has been considered a viable nutritional intervention for sarcopenia. Oral probiotics containing Lactobacillus roche and Lactobacillus galaei can reduce serum pro-inflammatory cytokine levels and improve muscle mass (Bindels et al., 2012). Karim et found the multistrain probiotic enhances muscle strength and functional performance in COPD patients by decreasing intestinal permeability and stabilizing the neuromuscular junction (Karim et al., 2022). Therefore, identifying gut microbiota biomarkers associated with sarcopenia and regulating dysbiosis through targeted interventions to supplement beneficial bacteria is crucial for the treatment of sarcopenia.

We performed an meta-analysis of 18 articles to discern differences in the gut microbiota diversity and composition between older people with and without sarcopenia. Through this analysis, we have pinpointed specific gut microbiota that demonstrate therapeutic potential as targets for sarcopenia intervention. However, there were several limitations of the study. First, our relatively small sample size and the participants from specific racial groups may limit the generalizability of our results. Second, the heterogeneity across studies may stem from a variety of factors, including subject-specific characteristics, diagnostic criteria for sarcopenia, sample collection and storage conditions, and differences in DNA extraction and sequencing techniques. Although we have conducted subgroup analyses of α-diversity for some confounding factors such as age and BMI, we were unable to fully reveal the role of all factors because the lack of relevant information. For example, protein and fiber intake have a significant effect on the gut microbiota of patients with sarcopenia. High-protein diets, especially animal protein intake, may promote the growth of certain protein-degrading bacteria, whereas plant-based proteins have the potential to have a positive effect on the abundance of probiotics (Strasser et al., 2021). Dietary fiber promotes the production of SCFAs by providing an energy source for beneficial intestinal bacteria (Holscher, 2017), which in turn improves gut health and enhances the diversity of intestinal microorganisms. On the other hand, medication use, especially antibiotics (Ramirez et al., 2020) and proton pump inhibitors (Imhann et al., 2016), may inhibit the growth of beneficial bacteria and promote the proliferation of harmful bacteria. In addition, we failed to perform subgroup analyses of β-diversity and relative abundance of gut microbiota. Third, the majority of the studies were based on 16S rRNA gene sequencing, which provided valuable information for identifying microbial community diversity but may not be a sufficiently deep approach to the species level. Fourth, the use of self-reported FFQs in included studies may result in measurement error. Fifth, our study showed a risk of bias for the Shannon and Simpson indices, but sensitivity analyses showed that our results were robust. In conclusion, the present study summarizes the characteristics of the gut microbiota in older people with sarcopenia. Future studies should aim to expand the sample size, incorporate more diverse populations, and use more advanced sequencing technologies to improve the accuracy and generalizability of the results. Moreover, a more thorough exploration and control of confounding factors will be essential to identify potential microbial targets in older adults with sarcopenia.

## Data Availability

The datasets presented in this study can be found in online repositories. The names of the repository/repositories and accession number(s) can be found in the article/**Supplementary Material**.
