# Supplementary material for: Comparison of the gut microbiota in older people with and without sarcopenia: a systematic review and meta-analysis
Source: Front Cell Infect Microbiol. 2025 Apr 28;15:1480293. doi: 10.3389/fcimb.2025.1480293 (PMC12066693; doi:10.3389/fcimb.2025.1480293)
Supplement: Supplementary file 1 [file DataSheet1.zip › Supplementary materials/Appendix 1. Electronic search strategy..pdf]

Appendix 1 Electronic search strategy in PubMed, Embase and Cochrane Library databases

PubMed

step	Search	Hits
#1	(((("Sarcopenia"[Mesh]) OR ("Muscle Weakness"[Mesh])) OR ("Muscular Atrophy"[Mesh])) OR (((((((((sarcopeni* [Title/Abstract])) OR (Presarcopenia[Title/Abstract])) OR (Myopenia[Title/Abstract])) OR (myoatrophy[Title/Abstract])) OR (Myophagism[Title/Abstract])) OR (myatrophy[Title/Abstract])) OR (myodegeneration[Title/Abstract])) OR (atrophy type 2[Title/Abstract])) OR (amyotroph*[Title/Abstract])))	71177
#2	"Gastrointestinal Microbiome"[Mesh]	39870
#3	((Gastrointestinal Microbiomes[Title/Abstract])) OR (Microbiome, Gastrointestinal[Title/Abstract])) OR (Gut Microbiome[Title/Abstract])) OR (Gut Microbiomes[Title/Abstract])) OR (Microbiome, Gut[Title/Abstract])) OR (Gut Microflora[Title/Abstract])) OR (Microflora, Gut[Title/Abstract])) OR (Gut Microbiota[Title/Abstract])) OR (Gut Microbiotas[Title/Abstract])) OR (Microbiota, Gut[Title/Abstract])) OR (Gastrointestinal Flora[Title/Abstract])) OR (Flora, Gastrointestinal[Title/Abstract])) OR (Gut Flora[Title/Abstract])) OR (Flora, Gut[Title/Abstract])) OR (Gastrointestinal Microbiota[Title/Abstract])) OR (Gastrointestinal Microbiotas[Title/Abstract])) OR (Microbiota, Gastrointestinal[Title/Abstract])) OR (Gastrointestinal Microbial Community[Title/Abstract])) OR (Gastrointestinal Microbial Communities[Title/Abstract])) OR (Microbial Community, Gastrointestinal[Title/Abstract])) OR (Gastrointestinal Microflora[Title/Abstract])) OR (Microflora, Gastrointestinal[Title/Abstract])) OR (Gastric Microbiome[Title/Abstract])) OR (Gastric Microbiomes[Title/Abstract])) OR (Microbiome, Gastric[Title/Abstract])) OR (Intestinal Microbiome[Title/Abstract])) OR (Intestinal Microbiomes[Title/Abstract])) OR (Microbiome, Intestinal[Title/Abstract])) OR (Intestinal Microbiota[Title/Abstract])) OR (Intestinal Microbiotas[Title/Abstract])) OR (Microbiota, Intestinal[Title/Abstract])) OR (Intestinal Microflora[Title/Abstract])) OR (Microflora, Intestinal[Title/Abstract])) OR (Intestinal Flora[Title/Abstract])) OR (Flora, Intestinal[Title/Abstract])) OR (Enteric Bacteria[Title/Abstract])) OR (Bacteria, Enteric[Title/Abstract])) OR (alimentary canal flora[Title/Abstract])) OR (alimentary tract flora[Title/Abstract])) OR (bowel flora[Title/Abstract])) OR (bowel microbiota[Title/Abstract])) OR (digestive canal flora[Title/Abstract])) OR (digestive tract flora[Title/Abstract])) OR (enteric flora[Title/Abstract])) OR (enteric microbiota[Title/Abstract])) OR (intestine flora[Title/Abstract])) OR (flora, intestine[Title/Abstract])) OR (gastrointestinal flora[Title/Abstract])) OR (gastrointestinal canal flora[Title/Abstract])) OR (gastrointestinal microbiome[Title/Abstract])) OR (gastrointestinal microbiota[Title/Abstract])) OR (gastrointestinal tract flora[Title/Abstract])) OR (gastrointestine flora[Title/Abstract])) OR (gastrointestine tract flora[Title/Abstract])) OR (gut bacteriagut microbiota[Title/Abstract])) OR (intestinal bacteria[Title/Abstract])) OR (intestinal bacterial flora[Title/Abstract])) OR (intestinal bacterium[Title/Abstract])) OR (intestinal canal flora[Title/Abstract])) OR (intestinal flora[Title/Abstract])) OR (intestinal microbe[Title/Abstract])) OR (intestinal microbes[Title/Abstract])) OR (intestinal microbiota[Title/Abstract])) OR (intestinal	97315

	microflora[Title/Abstract])) OR (intestinal microorganism[Title/Abstract])) OR (intestinal tract flora[Title/Abstract])) OR (intestine bacteria[Title/Abstract])) OR (intestine bacterium[Title/Abstract])) OR (intestine microbial flora[Title/Abstract])) OR (intestine microflora[Title/Abstract])) OR (faecal bacteria [Title/Abstract])) OR (faecal flora [Title/Abstract])) OR (faecal microbiota[Title/Abstract])) OR (faecal microflora [Title/Abstract])) OR (faeces flora [Title/Abstract])) OR (faeces microflora [Title/Abstract])) OR (fecal bacteria [Title/Abstract])) OR (fecal flora [Title/Abstract])) OR (fecal microbiota [Title/Abstract])) OR (fecal microflora [Title/Abstract])) OR (feces flora [Title/Abstract])	
#4	(((("Bacteria"[Mesh]) OR ("Microbiota"[Mesh])) OR ("Dysbiosis"[Mesh])) OR (((((((((((((((((((((((((Bacteria[Title/Abstract])) OR (Eubacteria[Title/Abstract])) OR (Eubacterium[Title/Abstract])) OR (Bacterial[Title/Abstract])) OR (bacteriologic[Title/Abstract])) OR (bacterium[Title/Abstract])) OR (Microbiotas[Title/Abstract])) OR (Microbiota[Title/Abstract])) OR (Microflora[Title/Abstract])) OR (Microbial[Title/Abstract])) OR (Microbiome[Title/Abstract])) OR (Microbiomes[Title/Abstract])) OR (Flora[Title/Abstract])) OR (Microorganism[Title/Abstract])) OR (Microorganisms[Title/Abstract])) OR (Microbe[Title/Abstract])) OR (microbes[Title/Abstract])) OR (Disbiosis[Title/Abstract])) OR (Dysbioses[Title/Abstract])) OR (Disbioses[Title/Abstract])) OR (Dys symbiosis[Title/Abstract])) OR (Dysbacteriosis[Title/Abstract])) OR (Dysbacterioses[Title/Abstract])) OR (Disbacteriosis[Title/Abstract])) OR (Dysbacteriosis[Title/Abstract]))) AND (((("Intestines"[Mesh]) OR ("Gastrointestinal Tract"[Mesh])) OR ("Feces"[Mesh])) OR (((((((((((((((((((((Gastrointestinal[Title/Abstract])) OR (bowel[Title/Abstract])) OR (Gut [Title/Abstract])) OR (Intestinal[Title/Abstract])) OR (Intestine[Title/Abstract])) OR (Intestines[Title/Abstract])) OR (intestinum[Title/Abstract])) OR (Enteric [Title/Abstract])) OR (alimentary tract[Title/Abstract])) OR (alimentary tracts[Title/Abstract])) OR (Alimentary canal[Title/Abstract])) OR (Alimentary canals[Title/Abstract])) OR (digestive tract[Title/Abstract])) OR (digestive tracts[Title/Abstract])) OR (digestive canal[Title/Abstract])) OR (digestive canals[Title/Abstract])) OR (GI Tract[Title/Abstract])) OR (GI Tracts[Title/Abstract])) OR (fecal [Title/Abstract])) OR (Feces[Title/Abstract])) OR (stool [Title/Abstract])))	254100
#5	#2 OR #3 OR #4	256278
#6	#1 AND #5	402
Limits: English		

Embase

step	Search	**Hits**
#1	'Sarcopenia'/exp OR 'Sarcopenias'/exp OR 'Sarcopenic'/exp OR 'Sarcopenie'/exp OR ' Muscle Weakness'/exp OR 'Muscle Weaknesses'/exp OR 'Weakness, Muscle'/exp OR 'Weaknesses, Muscle'/exp OR 'Muscular Weakness'/exp OR 'Muscular Weaknesses'/exp OR 'Weakness, Muscular'/exp OR 'Weaknesses, Muscular'/exp OR 'Muscular Atrophy '/exp OR 'Atrophies, Muscular'/exp OR 'Atrophy, Muscular'/exp OR 'Muscular Atrophies'/exp OR 'Atrophy, Muscle'/exp OR 'Atrophies, Muscle'/exp OR 'Muscle Atrophies'/exp OR 'Muscle Atrophy'/exp OR 'Neurogenic Muscular Atrophy'/exp OR 'Atrophies, Neurogenic	**520623**

	Muscular'/exp OR 'Atrophy, Neurogenic Muscular'/exp OR 'Muscular Atrophies, Neurogenic'/exp OR 'Muscular Atrophy, Neurogenic'/exp OR 'Neurogenic Muscular Atrophies'/exp OR 'Neurotrophic Muscular Atrophy'/exp OR 'Atrophies, Neurotrophic Muscular'/exp OR 'Atrophy, Neurotrophic Muscular'/exp OR 'Muscular Atrophies, Neurotrophic'/exp OR 'Muscular Atrophy, Neurotrophic'/exp OR 'Neurotrophic Muscular Atrophies'/exp OR 'amyotrophia'/exp OR 'amyotrophy'/exp OR 'atrophic muscular disorders'/exp OR 'atrophy type 2'/exp OR 'degeneration, muscle'/exp OR 'hirayama disease'/exp OR 'muscle atrophia'/exp OR 'muscle cell degeneration'/exp OR 'muscle degeneration'/exp OR 'muscle fiber atrophy'/exp OR 'muscle fiber degeneration'/exp OR 'muscle recession'/exp OR 'muscular degeneration'/exp OR 'muscular disorders, atrophic'/exp OR 'myoatrophy'/exp OR 'myodegeneration'/exp OR 'myofibrillar degeneration'/exp OR 'Myophagism'/exp OR 'muscle loss'/exp OR 'muscle wasting'/exp OR 'muscle insufficiency'/exp OR 'muscle depletion'/exp	
#2	'Gastrointestinal Microbiomes'/exp OR 'Microbiome, Gastrointestinal'/exp OR 'Gut Microbiome'/exp OR 'Gut Microbiomes'/exp OR 'Microbiome, Gut'/exp OR 'Gut Microflora'/exp OR 'Microflora, Gut'/exp OR 'Gut Microbiota'/exp OR 'Gut Microbiotas'/exp OR 'Microbiota, Gut'/exp OR 'Gastrointestinal Flora'/exp OR 'Flora, Gastrointestinal'/exp OR 'Gut Flora'/exp OR 'Flora, Gut'/exp OR 'Gastrointestinal Microbiota'/exp OR 'Gastrointestinal Microbiotas'/exp OR 'Microbiota, Gastrointestinal'/exp OR 'Gastrointestinal Microbial Community'/exp OR 'Gastrointestinal Microbial Communities'/exp OR 'Microbial Community, Gastrointestinal'/exp OR 'Gastrointestinal Microflora'/exp OR 'Microflora, Gastrointestinal'/exp OR 'Gastric Microbiome'/exp OR 'Gastric Microbiomes'/exp OR 'Microbiome, Gastric'/exp OR 'Intestinal Microbiome'/exp OR 'Intestinal Microbiomes'/exp OR 'Microbiome, Intestinal'/exp OR 'Intestinal Microbiota'/exp OR 'Intestinal Microbiotas'/exp OR 'Microbiota, Intestinal'/exp OR 'Intestinal Microflora'/exp OR 'Microflora, Intestinal'/exp OR 'Intestinal Flora'/exp OR 'Flora, Intestinal'/exp OR 'Enteric Bacteria'/exp OR 'Bacteria, Enteric'/exp OR 'alimentary canal flora'/exp OR 'alimentary tract flora'/exp OR 'bowel flora'/exp OR 'bowel microbiota'/exp OR 'digestive canal flora'/exp OR 'digestive tract flora'/exp OR 'enteric flora'/exp OR 'enteric microbiota'/exp OR 'intestine flora'/exp OR 'flora, intestine'/exp OR 'gastrointestinal flora'/exp OR 'gastrointestinal canal flora'/exp OR 'gastrointestinal microbiome'/exp OR 'gastrointestinal microbiota'/exp OR 'gastrointestinal tract flora'/exp OR 'gastrointestine flora'/exp OR 'gastrointestine tract flora'/exp OR 'gut bacteriagut microbiota'/exp OR 'intestinal bacteria'/exp OR 'intestinal bacterial flora'/exp OR 'intestinal bacterium'/exp OR 'intestinal canal flora'/exp OR 'intestinal flora'/exp OR 'intestinal microbe'/exp OR 'intestinal microbes'/exp OR 'intestinal microbiota'/exp OR 'intestinal microflora'/exp OR 'intestinal microorganism'/exp OR 'intestinal tract flora'/exp OR 'intestine bacteria'/exp OR 'intestine bacterium'/exp OR 'intestine microbial flora'/exp OR 'intestine microflora'/exp	**752790**
#3	('bacterium'/exp OR 'microflora'/exp) AND 'intestine'/exp	**64418**
#4	#2 OR #3	**783463**
#5	#1 AND #4	**3144**

Web of Science

step	Search	Hits
#1	((((((((((TS=(sarcopeni*)) OR TS=(Muscle Weakness)) OR TS=(Muscular Atrophy)) OR TS=(Presarcopenia)) OR TS=(Myopenia)) OR TS=(myoatrophy)) OR TS=(Myophagism)) OR TS=(myatrophy)) OR TS=(myodegeneration)) OR TS=(atrophy type 2)) OR TS=(amyotroph*)	**112795**
#2	(((TS=(Gastrointestinal Microbiomes)) OR TS=(Microbiome, Gastrointestinal)) OR TS=(Gut Microbiome)) OR TS=(Gut Microbiomes)) OR TS=(Microbiome, Gut)) OR TS=(Gut Microflora)) OR TS=(Microflora, Gut)) OR TS=(Gut Microbiota)) OR TS=(Gut Microbiotas)) OR TS=(Microbiota, Gut)) OR TS=(Gastrointestinal Flora)) OR TS=(Flora, Gastrointestinal)) OR TS=(Gut Flora)) OR TS=(Flora, Gut)) OR TS=(Gastrointestinal Microbiota)) OR TS=(Gastrointestinal Microbiotas)) OR TS=(Microbiota, Gastrointestinal)) OR TS=(Gastrointestinal Microbial Community)) OR TS=(Gastrointestinal Microbial Communities)) OR TS=(Microbial Community, Gastrointestinal)) OR TS=(Gastrointestinal Microflora)) OR TS=(Microflora, Gastrointestinal)) OR TS=(Gastric Microbiome)) OR TS=(Gastric Microbiomes)) OR TS=(Microbiome, Gastric)) OR TS=(Intestinal Microbiome)) OR TS=(Intestinal Microbiomes)) OR TS=(Microbiome, Intestinal)) OR TS=(Intestinal Microbiota)) OR TS=(Intestinal Microbiotas)) OR TS=(Microbiota, Intestinal)) OR TS=(Intestinal Microflora)) OR TS=(Microflora, Intestinal)) OR TS=(Intestinal Flora)) OR TS=(Flora, Intestinal)) OR TS=(Enteric Bacteria)) OR TS=(Bacteria, Enteric)) OR TS=(alimentary canal flora)) OR TS=(alimentary tract flora)) OR TS=(bowel flora)) OR TS=(bowel microbiota)) OR TS=(digestive canal flora)) OR TS=(digestive tract flora)) OR TS=(enteric flora)) OR TS=(enteric microbiota)) OR TS=(intestine flora)) OR TS=(flora, intestine)) OR TS=(gastrointestinal flora)) OR TS=(gastrointestinal canal flora)) OR TS=(gastrointestinal microbiome)) OR TS=(gastrointestinal microbiota)) OR TS=(gastrointestinal tract flora)) OR TS=(gastrointestine flora)) OR TS=(gastrointestine tract flora)) OR TS=(gut bacteriagut microbiota)) OR TS=(intestinal bacteria)) OR TS=(intestinal bacterial flora)) OR TS=(intestinal bacterium)) OR TS=(intestinal canal flora)) OR TS=(intestinal flora)) OR TS=(intestinal microbe)) OR TS=(intestinal microbes)) OR TS=(intestinal microbiota)) OR TS=(intestinal microflora)) OR TS=(intestinal microorganism)) OR TS=(intestinal tract flora)) OR TS=(intestine bacteria)) OR TS=(intestine bacterium)) OR TS=(intestine microbial flora)) OR TS=(intestine microflora)	**156976**
#3	(((((((((((((((((((((((((((TS=(Bacteria)) OR TS=(Eubacteria)) OR TS=(Eubacterium)) OR TS=(Bacterial)) OR TS=(bacteriologic)) OR TS=(bacterium)) OR TS=(Microbiotas)) OR TS=(Microbiota)) OR TS=(Microflora)) OR TS=(Microbial)) OR TS=(Microbiome)) OR TS=(Microbiomes)) OR TS=(Flora)) OR TS=(Microorganism)) OR TS=(Microorganisms)) OR TS=(Microbe)) OR TS=(microbes)) OR TS=(Disbiosis)) OR TS=(Dysbioses)) OR TS=(Disbioses)) OR TS=(Dys-symbiosis)) OR TS=(Dys symbiosis)) OR TS=(Dys-symbioses)) OR TS=(Dysbacteriosis)) OR TS=(Dysbacterioses)) OR TS=(Disbacteriosis)) OR TS=(Disbacterioses)) OR TS=(Dysbacteriosis)) AND (((((((((((((((((((((TS=(Gastrointestinal)) OR TS=(bowel)) OR TS=(Gut)) OR TS=(Intestinal)) OR TS=(Intestine)) OR TS=(Intestines)) OR TS=(intestinum)) OR TS=(Enteric)) OR TS=(alimentary tract)) OR TS=(alimentary tracts)) OR TS=(Alimentary	**224435**

	canal)) OR TS=(Alimentary canals)) OR TS=(digestive tract)) OR TS=(digestive tracts)) OR TS=(digestive canal)) OR TS=(digestive canals)) OR TS=(GI Tract)) OR TS=(GI Tracts)) OR TS=(fecal)) OR TS=(Feces)) OR TS=(stool))	
#4	#2 OR #3	**224647**
#5	#1 AND #4	**557**

Cochrane Library

step	Search	**Hits**
#1	MeSH descriptor: [Sarcopenia] explode all trees	**854**
#2	MeSH descriptor: [Muscle Weakness] explode all trees	**865**
#3	MeSH descriptor: [Muscular Atrophy] explode all trees	**1304**
#4	(sarcopeni*):ab,ti,kw OR (Presarcopenia):ab,ti,kw OR (Myopenia):ab,ti,kw OR (myoatrophy):ab,ti,kw OR (Myophagism):ab,ti,kw OR (myatrophy):ab,ti,kw OR (myodegeneration):ab,ti,kw OR (atrophy type 2):ab,ti,kw OR (amyotroph*):ab,ti,kw	**4742**
#5	(Muscle):ab,ti,kw OR (muscular):ab,ti,kw	**98657**
#6	(atroph*):ab,ti,kw OR (wasting):ab,ti,kw OR (weak*):ab,ti,kw OR (loss*):ab,ti,kw OR (depletion):ab,ti,kw OR (Insufficiency):ab,ti,kw OR (degeneration):ab,ti,kw OR (Dystrophy):ab,ti,kw	**154246**
#7	#5 AND #6	**16334**
#8	#1 OR #2 OR #3 OR #4 OR #7	**19751**
#9	MeSH descriptor: [Gastrointestinal Microbiome] explode all trees	**1362**
#10	(Gastrointestinal Microbiomes):ab,ti,kw OR (Microbiome, Gastrointestinal):ab,ti,kw OR (Gut Microbiome):ab,ti,kw OR (Gut Microbiomes):ab,ti,kw OR (Microbiome, Gut):ab,ti,kw OR (Gut Microflora):ab,ti,kw OR (Microflora, Gut):ab,ti,kw OR (Gut Microbiota):ab,ti,kw OR (Gut Microbiotas):ab,ti,kw OR (Microbiota, Gut):ab,ti,kw OR (Gastrointestinal Flora):ab,ti,kw OR (Flora, Gastrointestinal):ab,ti,kw OR (Gut Flora):ab,ti,kw OR (Flora, Gut):ab,ti,kw OR (Gastrointestinal Microbiota):ab,ti,kw OR (Gastrointestinal Microbiotas):ab,ti,kw OR (Microbiota, Gastrointestinal):ab,ti,kw OR (Gastrointestinal Microbial Community):ab,ti,kw OR (Gastrointestinal Microbial Communities):ab,ti,kw OR (Microbial Community, Gastrointestinal):ab,ti,kw OR (Gastrointestinal Microflora):ab,ti,kw OR (Microflora, Gastrointestinal):ab,ti,kw OR (Gastric Microbiome):ab,ti,kw OR (Gastric Microbiomes):ab,ti,kw OR (Microbiome, Gastric):ab,ti,kw OR (Intestinal Microbiome):ab,ti,kw OR (Intestinal Microbiomes):ab,ti,kw OR (Microbiome, Intestinal):ab,ti,kw OR (Intestinal Microbiota):ab,ti,kw OR (Intestinal Microbiotas):ab,ti,kw OR (Microbiota, Intestinal):ab,ti,kw OR (Intestinal Microflora):ab,ti,kw OR (Microflora, Intestinal):ab,ti,kw OR (Intestinal Flora):ab,ti,kw OR (Flora, Intestinal):ab,ti,kw OR (Enteric Bacteria):ab,ti,kw OR (Bacteria, Enteric):ab,ti,kw OR (alimentary canal flora):ab,ti,kw OR (alimentary tract flora):ab,ti,kw OR (bowel flora):ab,ti,kw OR (bowel microbiota):ab,ti,kw OR (digestive canal flora):ab,ti,kw OR (digestive tract flora):ab,ti,kw OR (enteric flora):ab,ti,kw OR (enteric microbiota):ab,ti,kw OR (intestine flora):ab,ti,kw OR (flora, intestine):ab,ti,kw OR (gastrointestinal flora):ab,ti,kw OR (gastrointestinal canal flora):ab,ti,kw OR (gastrointestinal microbiome):ab,ti,kw OR (gastrointestinal microbiota):ab,ti,kw OR	**11633**

	(gastrointestinal tract flora):ab,ti,kw OR (gastrointestine flora):ab,ti,kw OR (gastrointestine tract flora):ab,ti,kw OR (gut bacteriagut microbiota):ab,ti,kw OR (intestinal bacteria):ab,ti,kw OR (intestinal bacterial flora):ab,ti,kw OR (intestinal bacterium):ab,ti,kw OR (intestinal canal flora):ab,ti,kw OR (intestinal flora):ab,ti,kw OR (intestinal microbe):ab,ti,kw OR (intestinal microbes):ab,ti,kw OR (intestinal microbiota):ab,ti,kw OR (intestinal microflora):ab,ti,kw OR (intestinal microorganism):ab,ti,kw OR (intestinal tract flora):ab,ti,kw OR (intestine bacteria):ab,ti,kw OR (intestine bacterium):ab,ti,kw OR (intestine microbial flora):ab,ti,kw OR (intestine microflora):ab,ti,kw OR (faecal bacteria):ab,ti,kw OR (faecal flora):ab,ti,kw OR (faecal microbiota):ab,ti,kw OR (faecal microflora):ab,ti,kw OR (faeces flora):ab,ti,kw OR (faeces microflora):ab,ti,kw OR (fecal bacteria):ab,ti,kw OR (fecal flora):ab,ti,kw OR (fecal microbiota):ab,ti,kw OR (fecal microflora):ab,ti,kw OR (feces flora):ab,ti,kw	
#11	MeSH descriptor: [Bacteria] explode all trees	**17451**
#12	MeSH descriptor: [Microbiota] explode all trees	**1818**
#13	MeSH descriptor: [Dysbiosis] explode all trees	**202**
#14	(Bacteria):ab,ti,kw OR (Eubacteria):ab,ti,kw OR (Eubacterium):ab,ti,kw OR (Bacterial):ab,ti,kw OR (bacteriologic):ab,ti,kw OR (bacterium):ab,ti,kw OR (Microbiotas):ab,ti,kw OR (Microbiota):ab,ti,kw OR (Microflora):ab,ti,kw OR (Microbial):ab,ti,kw OR (Microbiome):ab,ti,kw OR (Microbiomes):ab,ti,kw OR (Flora):ab,ti,kw OR (Microorganism):ab,ti,kw OR (Microorganisms):ab,ti,kw OR (Microbe):ab,ti,kw OR (microbes):ab,ti,kw OR (Disbiosis):ab,ti,kw OR (Dysbioses):ab,ti,kw OR (Disbioses):ab,ti,kw OR (Dys symbiosis):ab,ti,kw OR (Dysbacteriosis):ab,ti,kw OR (Dysbacterioses):ab,ti,kw OR (Disbacteriosis):ab,ti,kw OR (Dysbacteriosis):ab,ti,kw OR (Disbacterioses):ab,ti,kw OR (Dys-symbiosis):ab,ti,kw OR (Dys-symbioses):ab,ti,kw	**59622**
#15	#11 OR #12 OR #13 OR #14	**64337**
#16	MeSH descriptor: [Gastrointestinal Tract] explode all trees	**15533**
#17	MeSH descriptor: [Intestines] explode all trees	**7874**
#18	MeSH descriptor: [Feces] explode all trees	**3869**
#19	(Gastrointestinal):ab,ti,kw OR (bowel):ab,ti,kw OR (Gut):ab,ti,kw OR (Intestinal):ab,ti,kw OR (Intestine):ab,ti,kw OR (Intestines):ab,ti,kw OR (intestinum):ab,ti,kw OR (Enteric):ab,ti,kw OR (alimentary tract):ab,ti,kw OR (alimentary tracts):ab,ti,kw OR (Alimentary canal):ab,ti,kw OR (Alimentary canals):ab,ti,kw OR (digestive tract):ab,ti,kw OR (digestive tracts):ab,ti,kw OR (digestive canal):ab,ti,kw OR (digestive canals):ab,ti,kw OR (GI Tract):ab,ti,kw OR (GI Tracts):ab,ti,kw OR (fecal):ab,ti,kw OR (Feces):ab,ti,kw OR (stool):ab,ti,kw	**104766**
#20	#16 OR #17 OR #18 OR #19	**112311**
#21	#15 AND #20	**18588**
#22	#9 OR #10 OR #21	**18593**
#23	#8 AND #22	**183**

万方数据库

step	Search	**Hits**
#1	((主题=肌少症) OR (主题=少肌症) OR (主题=肌减症) OR (主题=肌肉减少症) OR (主题=	**1089**

	骨骼肌减少症) OR (主题=骨骼肌衰减症) OR (主题=肌肉衰减综合征) OR (主题=老年性骨骼肌衰减) OR (主题=原发性老年肌肉衰减综合征) OR (主题=肌肉无力) OR (主题=肌肉萎缩) OR (主题=肌肉流失) OR (主题=肌肉损耗) OR (主题=肌肉衰退)) AND ((主题=肠道细菌) OR (主题=肠道菌群) OR (主题=肠道微生物) OR (主题=肠道微生态) OR (主题=粪便细菌) OR (主题=粪便菌群) OR (主题=粪便微生物) OR (主题=粪便微生态) OR (主题=消化道细菌) OR (主题=消化道菌群) OR (主题=消化道微生物) OR (主题=消化道微生态))	
限制	文献类型：期刊、学位、会议<br>发表时间：不限-至今<br>智能检索：中英文扩展&主题词扩展	

中国知网

Search	Hits
((SU%=肌少症) OR (SU%=少肌症) OR (SU%=肌减症) OR (SU%=肌肉减少症) OR (SU%=骨骼肌减少症) OR (SU%=骨骼肌衰减症) OR (SU%=肌肉衰减综合征) OR (SU%=老年性骨骼肌衰减) OR (SU%=原发性老年肌肉衰减综合征) OR (SU%=肌肉无力) OR (SU%=肌肉萎缩) OR (SU%=肌肉流失) OR (SU%=肌肉损耗) OR (SU%=肌肉衰退))AND((SU%=肠道细菌) OR (SU%=肠道菌群) OR (SU%=肠道微生物) OR (SU%=肠道微生态) OR (SU%=粪便细菌) OR (SU%=粪便菌群) OR (SU%=粪便微生物) OR (SU%=粪便微生态) OR (SU%=消化道细菌) OR (SU%=消化道菌群) OR (SU%=消化道微生物) OR (SU%=消化道微生态))	**81**
资源范围：总库<br>同义词扩展;<br>时间范围：更新时间：不限	
