# Supplementary material for: Comparison of the gut microbiota in older people with and without sarcopenia: a systematic review and meta-analysis
Source: Front Cell Infect Microbiol. 2025 Apr 28;15:1480293. doi: 10.3389/fcimb.2025.1480293 (PMC12066693; doi:10.3389/fcimb.2025.1480293)
Supplement: Supplementary file 1 [file DataSheet1.zip › Supplementary materials/Supplement Figure 3. Relative abundance (phyla-family).pdf]

A Phylum	Lee 2023	Ponziani 2021	Wang 2023*	Yamamoto 2022	Yan 2023	Zhang 2023	kang 2021	Total
Actinomycetota								2
Bacteroidota								3
Firmicutes								4
Proteobacteria								5
Verrucomicrobiota								2

B Phylum	Class	Yamamoto 2022	Zhang 2023
Firmicutes	Clostridia		
	Erysipelotrichi		
Bacteroidota	Bacteroidia		
Proteobacteria	Gammaproteobacteria		

C Phylum	Class	Order	Yamamoto 2022	Zhang 2023	Zhang 2024
Firmicutes	Clostridia	Clostridiales			
	Erysipelotrichia	Erysipelotrichales			
Bacteroidota	Bacteroidia	Bacteroidales			
	Flavobacteriia	Flavobacteriales			
Proteobacteria	Gammaproteobacteria	Enterobacteriales			

D Phylum	Class	Order	Family	Margiotta 2021	Picca 2019	Ponziani 2021	Yamamoto 2022	Zhang 2023	Zhang 2024	Total
Firmicutes	Negativicutes	Veillonellales	Veillonellaceae							2
	Clostridia	Peptostreptococcales	Peptostreptococcaceae							
		Eubacteriales	Oscillospiraceae							
	Bacilli	Lactobacillales	Enterococcaceae							
			Lactobacillaceae							
		Bacillales	Gemellaceae							
	Erysipelotrichia	Erysipelotrichales	Erysipelotrichaceae							2
Bacteroidota	Bacteroidia	Bacteroidales	Bacteroidaceae							2
			Rikenellaceae							
	Flavobacteriia	Flavobacteriales	Flavobacteriaceae							
Actinomycetota	Actinomycetes	Bifidobacteriales	Bifidobacteriaceae							
		Micrococcales	Micrococcaceae							
	Coriobacteriia	Coriobacteriales	Coriobacteriaceae							
Proteobacteria	Gammaproteobacteria	Enterobacterales	Enterobacteriaceae							
Verrucomicrobiota	Verrucomicrobiia	Verrucomicrobiotales	Verrucomicrobiotaceae							2

Supplement Figure 3. Changes in the relative abundance of microbes in the included studies. A Phylum level. B Class level. C Order. D Family. The red and blue grids indicate statistically significant increases and decreases in taxa with sarcopenia, respectively. In the total row, the numerical value represents the number of studies reporting significant changes in the taxa. Red grids indicate a significant increase, blue grids a significant decrease, and brown grids indicate both increases and decreases in sarcopenia.*Represents studies using shotgun metagenomic sequencing. Each microbe is labeled with the level to which it belongs.
