# Supplementary material for: Comparison of the gut microbiota in older people with and without sarcopenia: a systematic review and meta-analysis
Source: Front Cell Infect Microbiol. 2025 Apr 28;15:1480293. doi: 10.3389/fcimb.2025.1480293 (PMC12066693; doi:10.3389/fcimb.2025.1480293)
Supplement: Supplementary file 1 [file DataSheet1.zip › Supplementary materials/Supplement Table 2. Comparison between S and NS for the relative..docx]

**Supplemental Table 2.** Comparison between S and NS for the relative abundance of bacteria phyla, class, order, family, genus, and species.

| **Phyla of bacteria** | **Sarcopenia effect** | **P-value** | **Value NS vs S** | **Sample size NS vs S** | **Sarcopenia criteria** | **Ref** |
| --- | --- | --- | --- | --- | --- | --- |
| Actinomycetota | NS ＞ S | p ＞ 0.05 | 0.009 ± 0.007 vs 0.005 ± 0.005(MD) | 33 vs 27 | AWGS 2019 | Lee, 2022 |
| Actinomycetota | NS ＞ S | p ＞ 0.05 | 0.129 vs 0.09(M) | 16 vs 29 | AWGS 2019 | Lee, 2023 |
| ^Actinomycetota | NS ＜ S | p ＞ 0.05 | 0.013 vs 0.022(M) | 104 vs 88 | EWGSOP 2 | Wu, 2022 |
| ^Actinomycetota | NS ＜ S | p ＞ 0.05 | 0.068 vs 0.085(M) | 142 vs 141 | AWGS 2014 | Liu, 2023 |
| ^Actinomycetota | NS ＜ S | p ＞ 0.05 | -0.574*(DAA) | 17 vs 18 | FNIH | Picca, 2019 |
| ^Actinomycetota | NS ＞ S | p ＞ 0.05 | 0.664*(DAA) | 36 vs 14 | FNIH | Ponziani, 2021 |
| **^Actinomycetota** | **NS ＞ S** | **p ＜ 0.05** | **0.122 ± 0.126 vs 0.098 ± 0.083(MD)** | **29 vs 17** | **AWGS 2019** | **Yan, 2023** |
| **^Actinomycetota** | **NS ＜ S** | **p ＜ 0.05** | **0.0398 vs 0.0713(M)** | **50 vs 50** | **EWGSOP 2018 AWGS 2019** | **Wang, 2023** |
| **^Bacteroidota** | **NS ＜ S** | **p ＜ 0.05** | **0.027 ± 0.055 vs 0.038 ± 0.043(MD)** | **29 vs 17** | **AWGS 2019** | **Yan, 2023** |
| Bacteroidota | NS ＞ S | p ＞ 0.05 | 0.408 ± 0.149 vs 0.379 ± 0.161(MD) | 33 vs 27 | AWGS 2019 | Lee, 2022 |
| Bacteroidota | NS ＞ S | p ＞ 0.05 | 0.208 vs 0.192(M) | 16 vs 29 | AWGS 2019 | Lee, 2023 |
| **^Bacteroidota** | **NS ＞ S** | **p ＜ 0.05** | **0.493 vs 0. 417(M)** | **50 vs 50** | **EWGSOP 2018 AWGS 2019** | **Wang, 2023** |
| ^Bacteroidota | NS ＜ S | p ＞ 0.05 | 0.435 vs 0.452(M) | 104 vs 88 | EWGSOP 2 | Wu, 2022 |
| ^Bacteroidota | NS ＞ S | p ＞ 0.05 | 0.5 vs 0.477(M) | 142 vs 141 | AWGS 2014 | Liu, 2023 |
| **^Bacteroidota** | **N-SMI ＜ L-SMI** | **p ＜ 0.05** | **-4.722*(DAA)** | **44 vs 25** | **Other** | **Yamamoto, 2022** |
| ^Bacteroidota | NS ＞ S | p ＞ 0.05 | 0.335*(DAA) | 17 vs 18 | FNIH | Picca, 2019 |
| ^Bacteroidota | NS ＞ S | p ＞ 0.05 | 0.141*(DDA) | 36 vs 14 | FNIH | Ponziani, 2021 |
| Firmicutes | NS ＜ S | p ＞ 0.05 | 0.049 ± 0.091 vs 0.570 ± 0.154(MD) | 33 vs 27 | AWGS 2019 | Lee, 2022 |
| **^Firmicutes** | **NS ＞ S** | **p ＜ 0.05** | **0.722 ± 0.198 vs 0.652 ± 0.259(MD)** | **29 vs 17** | **AWGS 2019** | **Yan, 2023** |
| Firmicutes | NS ＞ S | p ＞ 0.05 | 0.601 vs 0.494(M) | 16 vs 29 | AWGS 2019 | Lee, 2023 |
| **^Firmicutes** | **NS ＜ S** | **p ＜ 0.05** | **0.4132 vs 0.4475(M)** | **50 vs 50** | **EWGSOP 2018 AWGS 2019** | **Wang, 2023** |
| **^Firmicutes** | **NS＞S** | **p＜0.05** | **0.544 vs 0.404 (M)** | **11 vs 60** | **AWGS 2019** | **Kang, 2021** |
| ^Firmicutes | NS ＜ S | p ＞ 0.05 | 0.440 vs 0.448(M) | 104 vs 88 | EWGSOP 2 | Wu, 2022 |
| ^Firmicutes | NS ＜ S | p ＞ 0.05 | 0.392 vs 0.398(M) | 142 vs 141 | AWGS 2014 | Liu, 2023 |
| **^Firmicutes** | **N-SMI ＞ L-SMI** | **p ＜ 0.05** | **4.693*(DAA)** | **44 vs 25** | **Other** | **Yamamoto, 2022** |
| ^Firmicutes | NS ＜ S | p ＞ 0.05 | -0.24*(DAA) | 17 vs 18 | FNIH | Picca, 2019 |
| ^Firmicutes | NS ＞ S | p ＞ 0.05 | 0.641*(DAA) | 36 vs 14 | FNIH | Ponziani, 2021 |
| Proteobacteria | NS ＞ S | p ＞ 0.05 | 0.040 ± 0.034 vs 0.015 ± 0.011(MD) | 33 vs 27 | AWGS 2019 | Lee, 2022 |
| **^Proteobacteria** | **NS ＜ S** | **p ＜ 0.05** | **0.126 ± 0.198 vs 0.207 ± 0.29(MD)** | **29 vs 17** | **AWGS 2019** | **Yan, 2023** |
| **Proteobacteria** | **NS ＜ S** | **p ＜ 0.05** | **0.034 vs 0.164(M)** | **16 vs 29** | **AWGS 2019** | **Lee, 2023** |
| **^Proteobacteria** | **NS ＞ S** | **p ＜ 0.05** | **0.0445 vs 0.036(M)** | **50 vs 50** | **EWGSOP 2018 AWGS 2019** | **Wang, 2023** |
| ^Proteobacteria | NS ＞ S | p ＞ 0.05 | 0.106 vs 0.071(M) | 104 vs 88 | EWGSOP 2 | Wu, 2022 |
| Proteobacteria | NS ＜ S | p ＞ 0.05 | 0.03 vs 0.034(M) | 142 vs 141 | AWGS 2014 | Liu, 2023 |
| **^Proteobacteria** | **N-SMI ＜ L-SMI** | **p ＜ 0.05** | **-4.226*(DAA)** | **44 vs 25** | **Other** | **Yamamoto, 2022** |
| ^Proteobacteria | NS ＜ S | p ＞ 0.05 | -1.348*(DAA) | 17 vs 18 | FNIH | Picca, 2019 |
| **^Proteobacteria** | **NS ＜ S** | **p ＜ 0.05** | **-4.9507*(DAA)** | **21 vs 14** | **AWGS 2019** | **Zhang, 2023** |
| ^Proteobacteria | NS ＜ S | p ＞ 0.05 | -1.239*(DAA) | 36 vs 14 | FNIH | Ponziani, 2021 |
| Verrucomicrobiota | NS ＜ S | p ＞ 0.05 | 0.026 ± 0.056 vs 0.0809 ± 0.073(MD) | 33 vs 27 | AWGS 2019 | Lee, 2022 |
| **Verrucomicrobiota** | **NS ＜ S** | **p ＜ 0.05** | **0.001 ± 0.002 vs 0.002 ± 0.004(MD)** | **29 vs 17** | **AWGS 2019** | **Yan, 2023** |
| Verrucomicrobiota | NS ＜ S | p ＞ 0.05 | 0.023 vs 0.039(M) | 16 vs 29 | AWGS 2019 | Lee, 2023 |
| Verrucomicrobiota | NS ＜ S | p ＞ 0.05 | 0.005 vs 0.006(M) | 142 vs 141 | AWGS 2014 | Liu, 2023 |
| ^Verrucomicrobiota | NS ＞ S | p ＞ 0.05 | 1.112*(DAA) | 17 vs 18 | FNIH | Picca, 2019 |
| **^Verrucomicrobiota** | **NS ＞ S** | **p ＜ 0.05** | **6.415*(DAA)** | **36 vs 14** | **FNIH** | **Ponziani, 2021** |
| ^TM7 | NS ＜ S | p ＞ 0.05 | -0.81*(DAA) | 17 vs 18 | FNIH | Picca, 2019 |
| ^TM7 | NS ＞ S | p ＞ 0.05 | 0.893*(DAA) | 36 vs 14 | FNIH | Ponziani, 2021 |
| ^Cyanobacteriota | NS ＜ S | p ＞ 0.05 | -0.824*(DAA) | 17 vs 18 | FNIH | Picca, 2019 |
| ^Cyanobacteriota | NS ＜ S | p ＞ 0.05 | -0.166*(DAA) | 36 vs 14 | FNIH | Ponziani, 2021 |
| ^Euryarchaeota | NS ＞ S | p ＞ 0.05 | 2.442*(DAA) | 36 vs 14 | FNIH | Ponziani, 2021 |
| Fusobacteriota | NS ＜ S | p ＞ 0.05 | 0.005 vs 0.006(M) | 104 vs 88 | EWGSOP 2 | Wu, 2022 |
| ^Synergistota | NS ＜ S | p ＞ 0.05 | -4.328*(DAA) | 17 vs 18 | FNIH | Picca, 2019 |
| **Class of bacteria** | **Sarcopenia effect** | **P-value** | **Value NS vs S** | **Sample size NS vs S** | **Sarcopenia criteria** | **Ref** |
| **^Clostridia** | **N-SMI ＞ L-SMI** | **p ＜ 0.05** | **4.7074*(DAA)** | **44 vs 25** | **Other** | **Yamamoto, 2022** |
| **^Erysipelotrichia** | **N-SMI ＞ L-SMI** | **p ＜ 0.05** | **3.7451*(DAA)** | **44 vs 25** | **Other** | **Yamamoto, 2022** |
| **^Bacteroidia** | **N-SMI ＜ L-SMI** | **p ＜ 0.05** | **-4.7289*(DAA)** | **44 vs 25** | **Other** | **Yamamoto, 2022** |
| **^Gammaproteobacteria** | **NS ＜ S** | **p ＜ 0.05** | **-4.9507*(DAA)** | **21 vs 14** | **AWGS 2019** | **Zhang, 2023** |
| **Order of bacteria** | **Sarcopenia effect** | **P-value** | **Value NS vs S** | **Sample size NS vs S** | **Sarcopenia criteria** | **Ref** |
| **^Clostridiales** | **N-SMI ＞ L-SMI** | **p ＜ 0.05** | **4.7074*(DAA)** | **44 vs 25** | **Other** | **Yamamoto, 2022** |
| **^Erysipelotrichales** | **N-SMI ＞ L-SMI** | **p ＜ 0.05** | **3.7522*(DAA)** | **44 vs 25** | **Other** | **Yamamoto, 2022** |
| **^Bacteroidales** | **N-SMI ＜ L-SMI** | **p ＜ 0.05** | **-4.7289*(DAA)** | **44 vs 25** | **Other** | **Yamamoto, 2022** |
| **Flavobacteriales** | **NS ＜ S** | **p＜0.05** | **0.00 ± 0.00 vs 0.0008 ± 0.001(MD)** | **31 vs 31** | **AWGS 2019** | **Zhang, 2024** |
| **^Enterobacteriales** | **NS ＜ S** | **p ＜ 0.05** | **-4.9507*(DAA)** | **21 vs 14** | **AWGS 2019** | **Zhang, 2023** |
| **Family of bacteria** | **Sarcopenia effect** | **P-value** | **Value NS vs S** | **Sample size NS vs S** | **Sarcopenia criteria** | **Ref** |
| Acidaminococcaceae | NS ＜ S | p ＞ 0.05 | 0.018 vs 0.022(M) | 16 vs 29 | AWGS 2019 | Lee, 2023 |
| Veillonellaceae | NS ＜ S | p ＞ 0.05 | 0.061 vs 0.07(M) | 16 vs 29 | AWGS 2019 | Lee, 2023 |
| **^Veillonellaceae** | **NS ＞ S** | **p ＜ 0.05** | **0.012(FDR)** | **45 vs 18** | **EWGSOP 2** | **Margiotta, 2021** |
| ^Veillonellaceae | NS ＜ S | p ＞ 0.05 | -0.906*(DAA) | 17 vs 18 | FNIH | Picca, 2019 |
| **^Veillonellaceae** | **NS ＜ S** | **p ＜ 0.05** | **-4.258*(DAA)** | **36 vs 14** | **FNIH** | **Ponziani, 2021** |
| Christensenellaceae | NS ＞ S | p ＞ 0.05 | 0.002 vs 0(M) | 16 vs 29 | AWGS 2019 | Lee, 2023 |
| ^Christensenellaceae | NS ＞ S | p ＞ 0.05 | 0.177*(DAA) | 17 vs 18 | FNIH | Picca, 2019 |
| ^Christensenellaceae | NS ＞ S | p ＞ 0.05 | 1.182*(DAA) | 36 vs 14 | FNIH | Ponziani, 2021 |
| Peptostreptococcaceae | NS ＜ S | p ＞ 0.05 | 0 vs 0.003(M) | 16 vs 29 | AWGS 2019 | Lee, 2023 |
| Peptostreptococcaceae | NS ＞ S | p ＞ 0.05 | 0.028 vs 0.013(M) | 16 vs 29 | AWGS 2019 | Lee, 2023 |
| **^Peptostreptococcaceae** | **NS ＜ S** | **p ＜ 0.05** | **-3.018*(DAA)** | **17 vs 18** | **FNIH** | **Picca, 2019** |
| ^Peptostreptococcaceae | NS ＜ S | p ＞ 0.05 | -1.213*(DAA) | 36 vs 14 | FNIH | Ponziani, 2021 |
| ^Mogibacteriaceae | NS ＞ S | p ＞ 0.05 | 0.544*(DAA) | 36 vs 14 | FNIH | Ponziani, 2021 |
| ^Mogibacteriaceae | NS ＞ S | p ＞ 0.05 | 0.422*(DAA) | 17 vs 18 | FNIH | Picca, 2019 |
| Clostridiaceae | NS ＜ S | p ＞ 0.05 | 0.002 vs 0.014(M) | 16 vs 29 | AWGS 2019 | Lee, 2023 |
| ^Clostridiaceae | NS ＜ S | p ＞ 0.05 | -1.422*(DAA) | 17 vs 18 | FNIH | Picca, 2019 |
| ^Clostridiaceae | NS ＞ S | p ＞ 0.05 | 0.052*(DAA) | 36 vs 14 | FNIH | Ponziani, 2021 |
| Oscillospiraceae | NS ＞ S | p ＞ 0.05 | 0.177 vs 0.051(M) | 16 vs 29 | AWGS 2019 | Lee, 2023 |
| ^Oscillospiraceae | NS ＞ S | p ＞ 0.05 | 0.117*(DAA) | 17 vs 18 | FNIH | Picca, 2019 |
| **^Oscillospiraceae** | **NS ＜ S** | **p ＜ 0.05** | **-1.266*(DAA)** | **36 vs 14** | **FNIH** | **Ponziani, 2021** |
| Lachnospiraceae | NS ＞ S | p ＞ 0.05 | 0.242 vs 0.167(M) | 16 vs 29 | AWGS 2019 | Lee, 2023 |
| ^Lachnospiraceae | NS ＜ S | p ＞ 0.05 | -0.509*(DAA) | 17 vs 18 | FNIH | Picca, 2019 |
| ^Lachnospiraceae | NS ＜ S | p ＞ 0.05 | -0.767*(DAA) | 36 vs 14 | FNIH | Ponziani, 2021 |
| Lachnospiraceae | NS ＜ S | p ＞ 0.05 | 0.008 ± 0.007 vs 0.01 ± 0.008(MD) | 29 vs 17 | AWGS 2019 | Yan, 2023 |
| Enterococcaceae | NS ＜ S | p ＞ 0.05 | 0.004 vs 0.007(M) | 16 vs 29 | AWGS 2019 | Lee, 2023 |
| **^Enterococcaceae** | **NS ＜ S** | **p ＜ 0.05** | **-3.7565*(DAA)** | **21 vs 14** | **AWGS 2019** | **Zhang, 2023** |
| ^Enterococcaceae | NS ＜ S | p ＞ 0.05 | -1.325*(DAA) | 17 vs 18 | FNIH | Picca, 2019 |
| ^Enterococcaceae | NS ＜ S | p ＞ 0.05 | -1.874*(DAA) | 36 vs 14 | FNIH | Ponziani, 2021 |
| Lactobacillaceae | NS ＜ S | p ＞ 0.05 | 0 vs 0.056(M) | 16 vs 29 | AWGS 2019 | Lee, 2023 |
| ^Lactobacillaceae | NS ＜ S | p ＞ 0.05 | -1.758*(DAA) | 17 vs 18 | FNIH | Picca, 2019 |
| **^Lactobacillaceae** | **NS ＜ S** | **p ＜ 0.05** | **-2.985*(DAA)** | **36 vs 14** | **FNIH** | **Ponziani, 2021** |
| Streptococcaceae | NS ＜ S | p ＞ 0.05 | 0.018 vs 0.074(M) | 16 vs 29 | AWGS 2019 | Lee, 2023 |
| ^Streptococcaceae | NS ＞ S | p ＞ 0.05 | 0.679*(DAA) | 17 vs 18 | FNIH | Picca, 2019 |
| ^Streptococcaceae | NS ＞ S | p ＞ 0.05 | 0.232*(DAA) | 36 vs 14 | FNIH | Ponziani, 2021 |
| ^Carnobacteriaceae | NS ＞ S | p ＞ 0.05 | 0.052*(DAA) | 17 vs 18 | FNIH | Picca, 2019 |
| ^Carnobacteriaceae | NS ＜ S | p ＞ 0.05 | -0.052*(DAA) | 36 vs 14 | FNIH | Ponziani, 2021 |
| **^Gemellaceae** | **NS ＞ S** | **p ＜ 0.05** | **0.042(FDR)** | **45 vs 18** | **EWGSOP 2** | **Margiotta, 2021** |
| Erysipelotrichaceae | NS ＞ S | p ＞ 0.05 | 0.056 vs 0.023(M) | 16 vs 29 | AWGS 2019 | Lee, 2023 |
| **^Erysipelotrichaceae** | **N-SMI ＞ L-SMI** | **p ＜ 0.05** | **3.752*(DAA)** | **44 vs 25** | **Other** | **Yamamoto, 2022** |
| ^Erysipelotrichaceae | NS ＞ S | p ＞ 0.05 | 0.682*(DAA) | 17 vs 18 | FNIH | Picca, 2019 |
| **^Erysipelotrichaceae** | **NS ＞ S** | **p ＜ 0.05** | **2.46*(DAA)** | **36 vs 14** | **FNIH** | **Ponziani, 2021** |
| ^Turicibacteraceae | NS ＞ S | p ＞ 0.05 | 0.307*(DAA) | 36 vs 14 | FNIH | Ponziani, 2021 |
| **Bacteroidaceae** | **NS ＜ S** | **p ＞ 0.05** | **0.099 vs 0.151(M)** | **16 vs 29** | **AWGS 2019** | **Lee, 2023** |
| **^Bacteroidaceae** | **N-SMI ＜ L-SMI** | **p ＜ 0.05** | **-4.736*(DAA)** | **44 vs 25** | **Other** | **Yamamoto, 2022** |
| **^Bacteroidaceae** | **NS ＞ S** | **p ＞ 0.05** | **0.175*(DAA)** | **17 vs 18** | **FNIH** | **Picca, 2019** |
| ^Bacteroidaceae | NS ＞ S | p ＞ 0.05 | 0.392*(DAA) | 36 vs 14 | FNIH | Ponziani,2021 |
| Barnesiellaceae | NS ＞ S | p ＞ 0.05 | 0.005 vs 0.003(M) | 16 vs 29 | AWGS 2019 | Lee, 2023 |
| ^Barnesiellaceae | NS ＜ S | p ＞ 0.05 | -0.278*(DAA) | 17 vs 18 | FNIH | Picca, 2019 |
| **^Barnesiellaceae** | **NS ＜ S** | **p ＜ 0.05** | **-2.496*(DAA)** | **36 vs 14** | **FNIH** | **Ponziani, 2021** |
| Prevotellaceae | NS ＞ S | p ＞ 0.05 | 0.086 vs 0.022(M) | 16 vs 29 | AWGS 2019 | Lee, 2023 |
| ^Prevotellaceae | NS ＞ S | p ＞ 0.05 | 1.649*(DAA) | 17 vs 18 | FNIH | Picca, 2019 |
| ^Prevotellaceae | NS ＞ S | p ＞ 0.05 | 1.413*(DAA) | 36 vs 14 | FNIH | Ponziani, 2021 |
| ^Paraprevotellaceae | NS ＞ S | p ＞ 0.05 | 1.573*(DAA) | 17 vs 18 | FNIH | Picca, 2019 |
| ^Paraprevotellaceae | NS ＞ S | p ＞ 0.05 | 1.925*(DAA) | 36 vs 14 | FNIH | Ponziani, 2021 |
| Rikenellaceae | NS ＞ S | p ＞ 0.05 | 0.013 vs 0.003(M) | 16 vs 29 | AWGS 2019 | Lee, 2023 |
| ^Rikenellaceae | NS ＜ S | p ＞ 0.05 | -1.225*(DAA) | 17 vs 18 | FNIH | Picca, 2019 |
| **^Rikenellaceae** | **NS ＜ S** | **p ＜ 0.05** | **-2.227*(DAA)** | **36 vs 14** | **FNIH** | **Ponziani, 2021** |
| ^Porphyromonadaceae | NS ＜ S | p ＞ 0.05 | -0.179*(DAA) | 17 vs 18 | FNIH | Picca, 2019 |
| ^Porphyromonadaceae | NS ＞ S | p ＞ 0.05 | 0.514*(DAA) | 36 vs 14 | FNIH | Ponziani, 2021 |
| ^Muribaculaceae | NS ＞ S | p ＞ 0.05 | 1.939*(DAA) | 17 vs 18 | FNIH | Picca, 2019 |
| **^Flavobacteriaceae** | **NS＜S** | **p＜0.05** | **0 ± 0 vs 0.0008 ± 0.001(MD)** | **31 vs 31** | **AWGS 2019** | **Zhang, 2024** |
| **^Bifidobacteriaceae** | **NS ＜ S** | **p ＜ 0.05** | **-2.136*(DAA)** | **17 vs 18** | **FNIH** | **Picca, 2019** |
| ^Bifidobacteriaceae | NS ＜ S | p ＞ 0.05 | -0.369*(DAA) | 36 vs 14 | FNIH | Ponziani, 2021 |
| **^Micrococcaceae** | **NS ＜ S** | **p ＜ 0.05** | **0.012(FDR)** | **45 vs 18** | **EWGSOP 2** | **Margiotta, 2021** |
| ^Micrococcaceae | NS ＜ S | p ＞ 0.05 | -0.181*(DAA) | 36 vs 14 | FNIH | Ponziani, 2021 |
| ^Coriobacteriaceae | NS ＞ S | p ＞ 0.05 | 0.051 vs 0.018(M) | 16 vs 29 | AWGS 2019 | Lee, 2023 |
| **^Coriobacteriaceae** | **NS ＞ S** | **p ＜ 0.05** | **3.6522*(DAA)** | **21 vs 14** | **AWGS 2019** | **Zhang, 2023** |
| ^Coriobacteriaceae | NS ＞ S | p ＞ 0.05 | 0.347*(DAA) | 17 vs 18 | FNIH | Picca, 2019 |
| ^Coriobacteriaceae | NS ＞ S | p ＞ 0.05 | 0.376*(DAA) | 36 vs 14 | FNIH | Ponziani, 2021 |
| Eggerthellaceae | NS ＜ S | p ＞ 0.05 | 0.002 vs 0.005(M) | 16 vs 29 | AWGS 2019 | Lee, 2023 |
| ^Alcaligenaceae | NS ＞ S | p ＞ 0.05 | 0.047*(DAA) | 17 vs 18 | FNIH | Picca, 2019 |
| **^Enterobacteriaceae** | **NS ＜ S** | **p ＜ 0.05** | **0.021 ± 0.013 vs 0.183 ± 0.099(MD)** | **21 vs 14** | **AWGS 2019** | **Zhang, 2023** |
| Enterobacteriaceae | NS ＜ S | p ＞ 0.05 | 0.024 vs 0.164(M) | 16 vs 29 | AWGS 2019 | Lee, 2023 |
| **^Enterobacteriaceae** | **NS ＜ S** | **p ＜ 0.05** | **-4.9507*(DAA)** | **21 vs 14** | **AWGS 2019** | **Zhang, 2023** |
| ^Enterobacteriaceae | NS ＜ S | p ＞ 0.05 | -1.101*(DAA) | 17 vs 18 | FNIH | Picca, 2019 |
| ^Enterobacteriaceae | NS ＜ S | p ＞ 0.05 | -1.295*(DAA) | 36 vs 14 | FNIH | Ponziani, 2021 |
| ^Pasteurellaceae | NS ＜ S | p ＞ 0.05 | -1.382*(DAA) | 17 vs 18 | FNIH | Picca, 2019 |
| ^Pasteurellaceae | NS ＞ S | p ＞ 0.05 | 0.278*(DAA) | 36 vs 14 | FNIH | Ponziani, 2021 |
| Marinifilaceae | NS ＜ S | p ＞ 0.05 | 0 vs 0.003(M) | 16 vs 29 | AWGS 2019 | Lee, 2023 |
| ^Dehalobacteriaceae | NS ＜ S | p ＞ 0.05 | **-**0.246*(DAA) | 17 vs 18 | FNIH | Picca, 2019 |
| ^Dehalobacteriaceae | NS ＞ S | p ＞ 0.05 | 1.102*(DAA) | 36 vs 14 | FNIH | Ponziani, 2021 |
| Akkermansiaceae | NS ＜ S | p ＞ 0.05 | 0.029 vs 0.041(M) | 16 vs 29 | AWGS 2019 | Lee, 2023 |
| **^Verrucomicrobiaceae** | **NS ＜ S** | **p ＜ 0.05** | **0.012(FDR)** | **45 vs 18** | **EWGSOP 2** | **Margiotta, 2021** |
| ^Verrucomicrobiaceae | NS ＞ S | p ＞ 0.05 | 0.738*(DAA) | 17 vs 18 | FNIH | Picca, 2019 |
| **^Verrucomicrobiaceae** | **NS ＞ S** | **p ＜ 0.05** | **3.707*(DAA)** | **36 vs 14** | **FNIH** | **Ponziani, 2021** |
| Fusobacteriaceae | NS ＜ S | p ＞ 0.05 | 0 vs 0.015(M) | 16 vs 29 | AWGS 2019 | Lee, 2023 |
| Synergistaceae | NS ＞ S | p ＞ 0.05 | 0.002 vs 0(M) | 16 vs 29 | AWGS 2019 | Lee, 2023 |
| ^Dethiosulfovibrionaceae | NS ＜ S | p ＞ 0.05 | -3.804*(DAA) | 17 vs 18 | FNIH | Picca, 2019 |
| ^Methanobacteriaceae | NS ＞ S | p ＞ 0.05 | 1.752*(DAA) | 36 vs 14 | FNIH | Ponziani, 2021 |
| ^Methanobacteriaceae | NS ＜ S | p ＞ 0.05 | -0.640*(DAA) | 17 vs 18 | FNIH | Picca, 2019 |
| Desulfovibrionaceae | NS ＞ S | p ＞ 0.05 | 0.006 vs 0(M) | 16 vs 29 | AWGS 2019 | Lee, 2023 |
| ^Desulfovibrionaceae | NS ＜ S | p ＞ 0.05 | -1.178*(DAA) | 17 vs 18 | FNIH | Picca, 2019 |
| ^Desulfovibrionaceae | NS ＜ S | p ＞ 0.05 | -1.713*(DAA) | 36 vs 14 | FNIH | Ponziani, 2021 |
| ^EtOH8 | NS ＞ S | p ＞ 0.05 | 0.077*(DAA) | 17 vs 18 | FNIH | Picca, 2019 |
| **Genus of bacteria** | **Sarcopenia effect** | **P-value** | **Value NS vs S** | **Sample size NS vs S** | **Sarcopenia criteria** | **Ref** |
| **^Mitsuokella** | **NS ＞ S** | **p ＜ 0.05** | **non-extractable** | **31 vs 31** | **AWGS 2019** | **Zhang, 2024** |
| Megamonas | NS ＜ S | p ＞ 0.05 | 0.016 vs 0.018(M) | 104 vs 88 | EWGSOP 2 | Wu, 2022 |
| Megamonas | NS ＞ S | p ＞ 0.05 | 0.030 vs 0.011(M) | 60 vs 11 | AWGS 2019 | Kang, 2021 |
| **^Allisonella** | **NS ＞ S** | **p ＜ 0.05** | **0.0058 ± 0.0067 vs 0.0015 ± 0.002** | **31 vs 31** | **AWGS 2019** | **Zhang, 2024** |
| ^Dialister | NS ＞ S | p ＞ 0.05 | 0.011 ± 0.021 vs 0.003 ± 0.007(MD) | 29 vs 17 | AWGS 2019 | Yan, 2023 |
| ^Dialister | NS ＞ S | p ＞ 0.05 | -3.586*(DAA) | 33 vs 27 | AWGS 2019 | Lee, 2022 |
| **^Dialister** | **NS ＜ S** | **p ＜ 0.05** | **-3.489*(DAA)** | **17 vs 18** | **FNIH** | **Picca, 2019** |
| ^Dialister | NS ＜ S | p ＞ 0.05 | 2.802*(DAA) | 36 vs 14 | FNIH | Ponziani, 2021 |
| **^Megasphaera** | **NS ＜ S** | **p ＜ 0.05** | **< 0.01 (FDR)** | **45 vs 18** | **EWGSOP 2** | **Margiotta, 2021** |
| **^Veillonella** | **NS ＜ S** | **p ＜ 0.05** | **0.012 (FDR)** | **45 vs 18** | **EWGSOP 2** | **Margiotta, 2021** |
| ^Veillonella | NS ＜ S | p ＞ 0.05 | -2.392*(DAA) | 17 vs 18 | FNIH | Picca, 2019 |
| ^Veillonella | NS ＜ S | p ＞ 0.05 | -3.04*(DAA) | 36 vs 14 | FNIH | Ponziani, 2021 |
| ^Phascolarctobacterium | NS ＜ S | p ＞ 0.05 | 3.161*(DAA) | 33 vs 27 | AWGS 2019 | Lee, 2022 |
| ^Phascolarctobacterium | NS ＞ S | p ＞ 0.05 | 0.004 ± 0.01 vs 0.003 ± 0.006(MD) | 29 vs 17 | AWGS 2019 | Yan, 2023 |
| ^Phascolarctobacterium | NS ＜ S | p ＞ 0.05 | -0.960*(DAA) | 17 vs 18 | FNIH | Picca, 2019 |
| ^Phascolarctobacterium | NS ＜ S | p ＞ 0.05 | -3.288*(DAA) | 36 vs 14 | FNIH | Ponziani, 2021 |
| Phascolarctobacterium | NS ＞ S | p ＞ 0.05 | 0.033 vs 0.023(M) | 104 vs 88 | EWGSOP 2 | Wu, 2022 |
| Phascolarctobacterium | NS ＞ S | p ＞ 0.05 | 0.014 vs 0.013(M) | 60 vs 11 | AWGS 2019 | Kang, 2021 |
| **^Acidaminococcus** | **NS ＞ S** | **p ＜ 0.05** | **< 0.0001 (FDR)** | **45 vs 18** | **EWGSOP 2** | **Margiotta, 2021** |
| **^Marvinbryantia** | **NM ＞ LM** | **p ＜ 0.05** | **0.035 ± 0.007 vs 0.006 ± 0.002%(MD)** | **52 vs 36** | **IWGS** | **Han, 2022** |
| **^Marvinbryantia** | **NM ＞ LM** | **p ＜ 0.05** | **0.0075(FDR)** | **52 vs 36** | **IWGS** | **Han, 2022** |
| **^Sellimonas** | **NM ＜ LM** | **p ＜ 0.05** | **0.015 ± 0.007% vs 0.047±0.021%(MD)** | **52 vs 36** | **IWGS** | **Han, 2022** |
| **^Sellimonas** | **NM ＜ LM** | **p ＜ 0.05** | **0.1724(FDR)** | **52 vs 36** | **IWGS** | **Han, 2022** |
| **Lachnospira** | **NS ＞ S** | **p ＜ 0.05** | **0.028 vs 0.007(M)** | **60 vs 11** | **AWGS 2019** | **Kang, 2021** |
| ^Lachnospira | NS ＞ S | p ＞ 0.05 | 0.561*(DAA) | 17 vs 18 | FNIH | Picca, 2019 |
| ^Lachnospira | NS ＞ S | p ＞ 0.05 | 0.735*(DAA) | 36 vs 14 | FNIH | Ponziani, 2021 |
| Lachnospira | NS ＞ S | p ＞ 0.05 | 0.016 vs 0.011(M) | 104 vs 88 | EWGSOP 2 | Wu, 2022 |
| ^Blautia | NS ＜ S | p ＞ 0.05 | 0.115 ± 0.078 vs 0.13 ± 0.095(MD) | 29 vs 17 | AWGS 2019 | Yan, 2023 |
| ^Blautia | NS ＜ S | p ＞ 0.05 | 1.998 vs 3.041(M) | 21 vs 14 | AWGS 2019 | Zhang, 2023 |
| Blautia | NS ＜ S | p ＞ 0.05 | 0.015 vs 0.022(M) | 104 vs 88 | EWGSOP 2 | Wu, 2022 |
| Blautia | NS ＜ S | p ＞ 0.05 | 0.028 vs 0.032(M) | 142 vs 141 | AWGS 2014 | Liu, 2023 |
| Blautia | NS ＞ S | p ＞ 0.05 | 0.062 vs 0.031(M) | 60 vs 11 | AWGS 2019 | Kang, 2021 |
| ^Blautia | NS ＞ S | p ＞ 0.05 | -0.246*(DAA) | 17 vs 18 | FNIH | Picca, 2019 |
| ^Blautia | NS ＞ S | p ＞ 0.05 | -1.023*(DAA) | 36 vs 14 | FNIH | Ponziani, 2021 |
| **^Agathobacter** | **NS ＞ S** | **p ＜ 0.05** | **0.038 ± 0.049 vs 0.022 ± 0.057(MD)** | **29 vs 17** | **AWGS 2019** | **Yan, 2023** |
| **^Fusicatenibacter** | **NS ＞ S** | **p ＜ 0.05** | **0.031 vs 0.004(M)** | **60 vs 11** | **AWGS 2019** | **Kang, 2021** |
| ^Fusicatenibacter | NS ＜ S | p ＞ 0.05 | 0.023 ± 0.026 vs 0.033 ± 0.035(MD) | 29 vs 17 | AWGS 2019 | Yan, 2023 |
| ^Anaerostipes | NS ＞ S | p ＞ 0.05 | 0.028 ± 0.038 vs 0.021 ± 0.033(MD) | 29 vs 17 | AWGS 2019 | Yan, 2023 |
| ^Anaerostipes | NS ＞ S | p ＞ 0.05 | 0.046*(DAA) | 17 vs 18 | FNIH | Picca, 2019 |
| ^Anaerostipes | NS ＞ S | p ＞ 0.05 | 1.106*(DAA) | 36 vs 14 | FNIH | Ponziani, 2021 |
| ^Coprococcus | NS ＞ S | p ＞ 0.05 | 0.014 ± 0.015 vs 0.007 ± 0.008(MD) | 29 vs 17 | AWGS 2019 | Yan, 2023 |
| ^Coprococcus | NS ＜ S | p ＞ 0.05 | -0.162*(DAA) | 17 vs 18 | FNIH | Picca, 2019 |
| ^Coprococcus | NS ＞ S | p ＞ 0.05 | 0.37*(DAA) | 36 vs 14 | FNIH | Ponziani, 2021 |
| **^Coprococcus** | **NS ＜ S** | **p ＜ 0.05** | **0.017 vs 0.025(M)** | **104 vs 88** | **EWGSOP 2** | **Wu, 2022** |
| ^Roseburia | NS ＜ S | p ＞ 0.05 | 0.006 ± 0.008 vs 0.008 ± 0.012(MD) | 29 vs 17 | AWGS 2019 | Yan, 2023 |
| ^Roseburia | NS ＞ S | p ＞ 0.05 | 0.024*(DAA) | 17 vs 18 | FNIH | Picca, 2019 |
| ^Roseburia | NS ＞ S | p ＞ 0.05 | 0.076*(DAA) | 36 vs 14 | FNIH | Ponziani, 2021 |
| **^Roseburia** | **NS ＜ S** | **p ＜ 0.05** | **-3.631*(DAA)** | **142 vs 141** | **AWGS 2014** | **Liu, 2023** |
| Roseburia | NS ＞ S | p ＞ 0.05 | 0.014 vs 0.012(M) | 104 vs 88 | EWGSOP 2 | Wu, 2022 |
| **Roseburia** | **NS ＜ S** | **p ＜ 0.05** | **0.052 vs 0.054(M)** | **142 vs 141** | **AWGS 2014** | **Liu, 2023** |
| **^Roseburia** | **NS ＞ S** | **p ＜ 0.05** | **0.035 vs 0.009(M)** | **60 vs 11** | **AWGS 2019** | **Kang, 2021** |
| **^Dorea** | **NM ＞ LM** | **p ＜ 0.05** | **0.620 ± 0.090% vs 0.363 ± 0.076%(MD)** | **52 vs 36** | **IWGS** | **Han, 2022** |
| **^Dorea** | **NM ＞ LM** | **p ＜ 0.05** | **0.0982(FDR)** | **52 vs 36** | **IWGS** | **Han, 2022** |
| **^Dorea** | **NS ＞ S** | **p ＜ 0.05** | **0.032 ± 0.024 vs 0.021 ± 0.017(MD)** | **29 vs 17** | **AWGS 2019** | **Yan, 2023** |
| ^Dorea | NS ＞ S | p ＞ 0.05 | 0.717*(DAA) | 17 vs 18 | FNIH | Picca, 2019 |
| ^Dorea | NS ＜ S | p ＞ 0.05 | 0.238*(DAA) | 36 vs 14 | FNIH | Ponziani, 2021 |
| ^Lachnobacterium | NS ＞ S | p ＞ 0.05 | 1.306*(DAA) | 17 vs 18 | FNIH | Picca, 2019 |
| ^Lachnobacterium | NS ＞ S | p ＞ 0.05 | 2.167*(DAA) | 36 vs 14 | FNIH | Ponziani, 2021 |
| Lachnospiraceae__unclassified | NS ＞ S | p ＞ 0.05 | 0.053 vs 0.052(M) | 142 vs 141 | AWGS 2014 | Liu, 2023 |
| **Lachnospiraceae__unclassified** | **NS ＞ S** | **p ＜ 0.05** | **0.011 vs 0.010(M)** | **104 vs 88** | **EWGSOP 2** | **Wu, 2022** |
| ^Lachnospiraceae_NK4A136_group | NS ＞ S | p ＞ 0.05 | 0.002 ± 0.004 vs 0.001 ± 0.001(MD) | 29 vs 17 | AWGS 2019 | Yan, 2023 |
| **^Lachnospiraceae_NK4A136_group** | **NS ＞ S** | **p ＜ 0.05** | **0.870 ± 0.176% vs 0.449 ± 0.112%(MD)** | **52 vs 36** | **IWGS** | **Han, 2022** |
| **^Lachnospiraceae_NK4A136_group** | **NS ＞ S** | **p ＜ 0.05** | **0.3104(FDR)** | **52 vs 36** | **IWGS** | **Han, 2022** |
| **^Lachnospiraceae_UCG-010** | **NM ＞ LM** | **p ＜ 0.05** | **0.209 ± 0.035% vs 0.152 ± 0.049%(MD)** | **52 vs 36** | **IWGS** | **Han, 2022** |
| **^Lachnospiraceae_UCG-010** | **NM ＞ LM** | **p ＜ 0.05** | **0.2153(FDR)** | **52 vs 36** | **IWGS** | **Han, 2022** |
| **^Lachnospiraceae_FCS020_group** | **NS＜S** | **p ＜ 0.05** | **0.004 ± 0.0042 vs 0.0119 ± 0.012(MD)** | **31 vs 31** | **AWGS 2019** | **Zhang, 2024** |
| **^Lachnoclostridium** | **NS ＞ S** | **p ＜ 0.05** | **0.025 vs 0.011(M)** | **60 vs 11** | **AWGS 2019** | **Kang, 2021** |
| ^Lachnoclostridium | NS ＜ S | p ＞ 0.05 | 0.001 ± 0.001 vs 0.004 ± 0.006(MD) | 29 vs 17 | AWGS 2019 | Yan, 2023 |
| **^Lachnoclostridium_5** | **NM ＜ LM** | **p ＜ 0.05** | **0.00 ± 0.00% vs 0.007 ± 0.005%(MD)** | **52 vs 36** | **IWGS** | **Han, 2022** |
| **^Lachnoclostridium_5** | **NM ＜ LM** | **p ＜ 0.05** | **0.295(FDR)** | **52 vs 36** | **IWGS** | **Han, 2022** |
| ^Ruminococcus_torques_group | NS ＜ S | p ＞ 0.05 | 0.02 ± 0.033 vs 0.026 ± 0.029(MD) | 29 vs 17 | AWGS 2019 | Yan, 2023 |
| ^Ruminococcus_gauvreauii_group | NS ＞ S | p ＞ 0.05 | 0.01 ± 0.014 vs 0.005 ± 0.006(MD) | 29 vs 17 | AWGS 2019 | Yan, 2023 |
| ^Ruminococcus_gnavus_group | NS ＜ S | p ＞ 0.05 | 0.001 ± 0.001 vs 0.004 ± 0.014(MD) | 29 vs 17 | AWGS 2019 | Yan, 2023 |
| ^Hungatella | NS ＜ S | p ＞ 0.05 | 0 ± 0 vs 0.001 ± 0.003(MD) | 29 vs 17 | AWGS 2019 | Yan, 2023 |
| ^Tyzzerella | NS ＞ S | p ＞ 0.05 | 0.004 ± 0.013 vs 0.003 ± 0.006(MD) | 29 vs 17 | AWGS 2019 | Yan, 2023 |
| ^Ruminococcus | NS ＞ S | p ＞ 0.05 | 0.036 ± 0.031 vs 0.028 ± 0.035(MD) | 29 vs 17 | AWGS 2019 | Yan, 2023 |
| ^Ruminococcus | NS ＜ S | p ＞ 0.05 | -0.623*(DAA) | 17 vs 18 | FNIH | Picca, 2019 |
| ^Ruminococcus | NS ＜ S | p ＞ 0.05 | -0.201*(DAA) | 17 vs 18 | FNIH | Picca, 2019 |
| ^Ruminococcus | NS ＜ S | p ＞ 0.05 | -1.183*(DAA) | 36 vs 14 | FNIH | Ponziani, 2021 |
| ^Ruminococcus | NS ＜ S | p ＞ 0.05 | -0.5*(DAA) | 36 vs 14 | FNIH | Ponziani, 2021 |
| **^Ruminococcus** | **NS ＞ S** | **p ＜ 0.05** | **0.015 vs 0.014(M)** | **104 vs 88** | **EWGSOP 2** | **Wu, 2022** |
| **^Ruminiclostridium_9** | **NM ＞ LM** | **p ＜ 0.05** | **0.119 ± 0.023% vs 0.092 ± 0.024%(MD)** | **52 vs 36** | **IWGS** | **Han, 2022** |
| **^Ruminiclostridium_9** | **NM ＞ LM** | **p ＜ 0.05** | **0.3489 (FDR)** | **52 vs 36** | **IWGS** | **Han, 2022** |
| **^Subdoligranulum** | **NM ＞ LM** | **p ＜ 0.05** | **2.339 ± 0.323% vs 1.412 ± 0.395%(MD)** | **52 vs 36** | **IWGS** | **Han, 2022** |
| **^Subdoligranulum** | **NM ＞ LM** | **p ＜ 0.05** | **0.0618(FDR)** | **52 vs 36** | **IWGS** | **Han, 2022** |
| Subdoligranulum | NS ＞ S | p ＞ 0.05 | 0.025 vs 0.024(M) | 60 vs 11 | AWGS 2019 | Kang, 2021 |
| ^Anaerotruncus | NS ＜ S | p ＞ 0.05 | 2.391*(DAA) | 33 vs 27 | AWGS 2019 | Lee, 2022 |
| ^Anaerotruncus | NS ＞ S | p ＞ 0.05 | 1.043*(DAA) | 17 vs 18 | FNIH | Picca, 2019 |
| ^Faecalibacterium | NS ＜ S | p ＞ 0.05 | 0.046 ± 0.054 vs 0.047 ± 0.046(MD) | 29 vs 17 | AWGS 2019 | Yan, 2023 |
| ^Faecalibacterium | NS ＞ S | p ＞ 0.05 | 0.907*(DAA) | 17 vs 18 | FNIH | Picca, 2019 |
| ^Faecalibacterium | NS ＜ S | p ＞ 0.05 | 1.775*(DAA) | 36 vs 14 | FNIH | Ponziani, 2021 |
| Faecalibacterium | NS ＜ S | p ＞ 0.05 | 0.045 vs 0.056(M) | 104 vs 88 | EWGSOP 2 | Wu, 2022 |
| Faecalibacterium | NS ＜ S | p ＞ 0.05 | 0.04 vs 0.047(M) | 142 vs 141 | AWGS 2014 | Liu, 2023 |
| Faecalibacterium | NS ＞ S | p ＞ 0.05 | 0.105 vs 0.050(M) | 60 vs 11 | AWGS 2019 | Kang, 2021 |
| **Faecalibacterium** | **NS ＞ S** | **p ＜ 0.05** | **3.910 vs 2.346(M)** | **21 vs 14** | **AWGS 2019** | **Zhang, 2023** |
| ^Oscillospira | NS ＜ S | p ＞ 0.05 | -0.536* (DAA) | 17 vs 18 | FNIH | Picca, 2019 |
| ^Oscillospira | NS ＜ S | p ＞ 0.05 | -0.659*(DAA) | 36 vs 14 | FNIH | Ponziani, 2021 |
| **^Flavonifractor** | **NM ＜ LM** | **p ＜ 0.05** | **0.164 ± 0.041% vs 0.419 ± 0.083%(MD)** | **52 vs 36** | **IWGS** | **Han, 2022** |
| **^Oscillospiraceae_UCG-003** | **NM ＞ LM** | **p ＜ 0.05** | **0.280 ± 0.036% vs 0.149 ± 0.040%(MD)** | **52 vs 36** | **IWGS** | **Han, 2022** |
| **^Oscillospiraceae_UCG-003** | **NM ＞ LM** | **p ＜ 0.05** | **0.0618(FDR)** | **52 vs 36** | **IWGS** | **Han, 2022** |
| **^Oscillospiraceae_UCG-010** | **NM ＞ LM** | **p ＜ 0.05** | **0.057 ± 0.021% vs 0.003 ± 0.002%(MD)** | **52 vs 36** | **IWGS** | **Han, 2022** |
| **^Oscillospiraceae_UCG-010** | **NM ＞ LM** | **p ＜ 0.05** | **0.0618(FDR)** | **52 vs 36** | **IWGS** | **Han, 2022** |
| **^Oscillospiraceae_UCG-005** | **NM ＞ LM** | **p ＜ 0.05** | **0.242 ± 0.054% vs 0.058 ± 0.018%(MD)** | **52 vs 36** | **IWGS** | **Han, 2022** |
| **^Oscillospiraceae_UCG-005** | **NM ＞ LM** | **p ＜ 0.05** | **0.1350(FDR)** | **52 vs 36** | **IWGS** | **Han, 2022** |
| **^Oscillospiraceae_UCG-002** | **NM ＞ LM** | **p ＜ 0.05** | **1.169 ± 0.168% vs 0.788 ± 0.232%(MD)** | **52 vs 36** | **IWGS** | **Han, 2022** |
| **^Oscillospiraceae_UCG-002** | **NM ＞ LM** | **p ＜ 0.05** | **0.2153(FDR)** | **52 vs 36** | **IWGS** | **Han, 2022** |
| **^Oscillospiraceae_UCG-014** | **NM ＞ LM** | **p ＜ 0.05** | **1.042 ± 0.301% vs 0.495 ± 0.196%(MD)** | **52 vs 36** | **IWGS** | **Han, 2022** |
| **^Oscillospiraceae_UCG-014** | **NM ＞ LM** | **p ＜ 0.05** | **0.3104(FDR)** | **52 vs 36** | **IWGS** | **Han, 2022** |
| **^Oscillospiraceae_NK4A214_group** | **NM ＞ LM** | **p ＜ 0.05** | **0.195 ± 0.047% vs 0.062 ± 0.031%(MD)** | **52 vs 36** | **IWGS** | **Han, 2022** |
| **^Oscillospiraceae_NK4A214_group** | **NM ＞ LM** | **p ＜ 0.05** | **0.0979(FDR)** | **52 vs 36** | **IWGS** | **Han, 2022** |
| ^Oscillospiraceae_unclassified | NS ＞ S | p ＞ 0.05 | 0.087 vs 0.082(M) | 104 vs 88 | EWGSOP 2 | Wu, 2022 |
| ^Oscillospiraceae_unclassified | NS ＜ S | p ＞ 0.05 | 3.793*(DAA) | 33 vs 27 | AWGS 2019 | Lee, 2022 |
| **^Peptococcus** | **NM ＞ LM** | **p ＜ 0.05** | **0.032 ± 0.011% vs 0.006 ± 0.004%(MD)** | **52 vs 36** | **IWGS** | **Han, 2022** |
| **^Peptococcus** | **NM＞LM** | **p ＜ 0.05** | **0.1900(FDR)** | **52 vs 36** | **IWGS** | **Han, 2022** |
| **^Clostridium** | **N-SMI ＞ L-SMI** | **p ＜ 0.05** | **3.601*(DAA)** | **44 vs 25** | **Other** | **Yamamoto, 2022** |
| ^Clostridium_sensu_stricto_1 | NS ＜ S | p ＞ 0.05 | 0.025 ± 0.046 vs 0.026 ± 0.039(MD) | 29 vs 17 | AWGS 2019 | Yan, 2023 |
| **^Clostridium_innocuum_group** | **NS ＜ S** | **p ＜ 0.05** | **-3.168*(DAA)** | **21 vs 14** | **AWGS 2019** | **Zhang, 2023** |
| **^Eubacterium** | **NS ＞ S** | **p ＜ 0.05** | **3.183*(DAA)** | **17 vs 18** | **FNIH** | **Picca, 2019** |
| ^Eubacterium | NS ＞ S | p ＞ 0.05 | 0.044 vs 0.038(M) | 142 vs 141 | AWGS 2014 | Liu, 2023 |
| ^Eubacterium | NS ＞ S | p ＞ 0.05 | 2.726*(DAA) | 36 vs 14 | FNIH | Ponziani, 2021 |
| **^Eubacterium_coprostanoligenes**  **_group** | **NM ＞ LM** | **p ＜ 0.05** | **1.421 ± 0.295% vs 0.566 ± 0.145%(MD)** | **52 vs 36** | **IWGS** | **Han, 2022** |
| **^Eubacterium_coprostanoligenes**  **_group** | **NM ＞ LM** | **p ＜ 0.05** | **0.3195(FDR)** | **52 vs 36** | **IWGS** | **Han, 2022** |
| ^Eubacterium_coprostanoligenes_group | NS ＞ S | p ＞ 0.05 | 0.009 ± 0.009 vs 0.006 ± 0.008(MD) | 29 vs 17 | AWGS 2019 | Yan, 2023 |
| ^Eubacterium_hallii_group | NS ＞ S | p ＞ 0.05 | 0.042 ± 0.029 vs 0.033 ± 0.025(MD) | 29 vs 17 | AWGS 2019 | Yan, 2023 |
| ^Eubacterium_ruminantium_group | NS ＞ S | p ＞ 0.05 | 0.002 ± 0.006 vs 0 ± 0.001(MD) | 29 vs 17 | AWGS 2019 | Yan, 2023 |
| ^Eubacterium_siraeum_group | NS ＞ S | p ＞ 0.05 | 0.003 ± 0.006 vs 0.001 ± 0.002(MD) | 29 vs 17 | AWGS 2019 | Yan, 2023 |
| **^Eubacterium_rectale_group** | **NS ＞ S** | **p ＜ 0.05** | **0.058 vs 0.018(M)** | **60 vs 11** | **AWGS 2019** | **Kang, 2021** |
| ^Dehalobacterium | NS ＜ S | p ＞ 0.05 | -0.00004*(DAA) | 17 vs 18 | FNIH | Picca, 2019 |
| ^Dehalobacterium | NS ＞ S | p ＞ 0.05 | 1.644*(DAA) | 36 vs 14 | FNIH | Ponziani, 2021 |
| Clostridiales_unclassified | NS ＜ S | p ＞ 0.05 | 0.025 vs 0.029(M) | 104 vs 88 | EWGSOP 2 | Wu, 2022 |
| ^Monoglobus | NS ＜ S | p ＞ 0.05 | 0.004 ± 0.004 vs 0.007 ± 0.008(MD) | 29 vs 17 | AWGS 2019 | Yan, 2023 |
| ^Christensenella | NS ＞ S | p ＞ 0.05 | 0.422*(DAA) | 17 vs 18 | FNIH | Picca, 2019 |
| ^Christensenella | NS ＞ S | p ＞ 0.05 | 0.438*(DAA) | 36 vs 14 | FNIH | Ponziani, 2021 |
| **^Christensenellaceae_R-7_group** | **NM ＞ LM** | **p ＜ 0.05** | **0.483 ± 0.159% vs 0.131 ± 0.038%(MD)** | **52 vs 36** | **IWGS** | **Han, 2022** |
| **^Christensenellaceae_R-7_group** | **NM ＞ LM** | **p ＜ 0.05** | **0.0982(FDR)** | **52 vs 36** | **IWGS** | **Han, 2022** |
| Christensenellaceae R-7 group | NS ＜ S | p ＞ 0.05 | 0.012 vs 0.019(M) | 60 vs 11 | AWGS 2019 | Kang, 2021 |
| **^Leuconostoc** | **NM ＞ LM** | **p ＜ 0.05** | **0.004 ± 0.001% vs 0.00 ± 0.00%(MD)** | **52 vs 36** | **IWGS** | **Han, 2022** |
| **^Leuconostoc** | **NM ＞ LM** | **p ＜ 0.05** | **0.0982(FDR)** | **52 vs 36** | **IWGS** | **Han, 2022** |
| ^Streptococcus | NS ＞ S | p ＞ 0.05 | 0.041 ± 0.121 vs 0.021 ± 0.029(MD) | 29 vs 17 | AWGS 2019 | Yan, 2023 |
| ^Streptococcus | NS ＞ S | p ＞ 0.05 | 0.526*(DAA) | 17 vs 18 | FNIH | Picca, 2019 |
| ^Streptococcus | NS ＞ S | p ＞ 0.05 | -0.327*(DAA) | 36 vs 14 | FNIH | Ponziani, 2021 |
| ^Lactococcus | NS ＞ S | p ＞ 0.05 | 0.001 ± 0.004 vs 0 ± 0(MD) | 29 vs 17 | AWGS 2019 | Yan, 2023 |
| ^Lactobacillus | NS ＜ S | p ＞ 0.05 | 0.002 ± 0.005 vs 0.011 ± 0.026(MD) | 29 vs 17 | AWGS 2019 | Yan, 2023 |
| ^Lactobacillus | NS ＜ S | p ＞ 0.05 | -2.619*(DAA) | 17 vs 18 | FNIH | Picca, 2019 |
| **^Lactobacillus** | **NS ＜ S** | **p ＜ 0.05** | **-4.785*(DAA)** | **36 vs 14** | **FNIH** | **Ponziani, 2021** |
| **^Lactobacillus** | **NS ＜ S** | **p ＜ 0.05** | **0.008 vs 0.046(M)** | **60 vs 11** | **AWGS 2019** | **Kang, 2021** |
| Lactobacillus | NS ＞ S | p ＞ 0.05 | 0.013 vs 0.011(M) | 104 vs 88 | EWGSOP 2 | Wu, 2022 |
| ^Lactobacillus | NS ＜ S | p ＞ 0.05 | 4.344 vs 4.778(M) | 21 vs 14 | AWGS 2019 | Zhang, 2023 |
| ^Enterococcus | NS ＜ S | p ＞ 0.05 | 0.007 ± 0.017 vs 0.008 ± 0.031(MD) | 29 vs 17 | AWGS 2019 | Yan, 2023 |
| **^Enterococcus** | **NS ＜ S** | **p ＜ 0.05** | **-3.7565*(DAA)** | **21 vs 14** | **AWGS 2019** | **Zhang, 2023** |
| ^Enterococcus | NS ＜ S | p ＞ 0.05 | -1.294*(DAA) | 17 vs 18 | FNIH | Picca, 2019 |
| ^Enterococcus | NS ＜ S | p ＞ 0.05 | -1.93*(DAA) | 36 vs 14 | FNIH | Ponziani, 2021 |
| ^Granulicatella | NS ＜ S | p ＞ 0.05 | -0.110*(DAA) | 17 vs 18 | FNIH | Picca, 2019 |
| ^Granulicatella | NS ＞ S | p ＞ 0.05 | 0.832*(DAA) | 36 vs 14 | FNIH | Ponziani, 2021 |
| **^Gemella** | **NS ＞ S** | **p ＜ 0.05** | **0.03 (FDR)** | **45 vs 18** | **EWGSOP 2** | **Margiotta, 2021** |
| ^Intestinibacter | NS ＞ S | p ＞ 0.05 | 0.011 ± 0.019 vs 0.005 ± 0.007(MD) | 29 vs 17 | AWGS 2019 | Yan, 2023 |
| **^Terrisporobacter** | **NM ＞ LM** | **p ＜ 0.05** | **0.011 ± 0.003% vs 0.002 ± 0.002%(MD)** | **52 vs 36** | **IWGS** | **Han, 2022** |
| **^Terrisporobacter** | **NM ＞ LM** | **p ＜ 0.05** | **0.2661(FDR)** | **52 vs 36** | **IWGS** | **Han, 2022** |
| ^Romboutsia | NS ＞ S | p ＞ 0.05 | 0.045 ± 0.065 vs 0.027 ± 0.043(MD) | 29 vs 17 | AWGS 2019 | Yan, 2023 |
| ^Romboutsia | NS ＜ S | p ＜ 0.05 | 0 ± 0 vs 0 ± 0 | 31 vs 31 | AWGS 2019 | Zhang, 2024 |
| ^Erysipelotrichaceae_UCG-003 | NS ＞ S | p ＞ 0.05 | 0.017 ± 0.024 vs 0.016 ± 0.018(MD) | 29 vs 17 | AWGS 2019 | Yan, 2023 |
| **^Erysipelotrichaceae_UCG-003** | **NS＜S** | **p ＜ 0.05** | **0.0028 ± 0.0035 vs 0.017 ± 0.0197** | **31 vs 31** | **AWGS 2019** | **Zhang, 2024** |
| ^Holdemanella | NS ＜ S | p ＞ 0.05 | 0.004 ± 0.008 vs 0.005 ± 0.014(MD) | 29 vs 17 | AWGS 2019 | Yan, 2023 |
| **^Coprobacillus** | **N-SMI ＞ L-SMI** | **p ＜ 0.05** | **3.6454*(DAA)** | **44 vs 25** | **Other** | **Yamamoto, 2022** |
| **^Coprobacillus** | **NS ＜ S** | **p ＜ 0.05** | **0.01(FDR)** | **45 vs 18** | **EWGSOP 2** | **Margiotta, 2021** |
| **^Catenibacterium** | **N-SMI ＞ L-SMI** | **p ＜ 0.05** | **3.4291*(DAA)** | **44 vs 25** | **Other** | **Yamamoto, 2022** |
| ^Catenibacterium | NS ＞ S | p ＞ 0.05 | -4.176*(DAA) | 36 vs 14 | FNIH | Ponziani, 2021 |
| **^Catenibacterium** | **NS＜S** | **p ＜ 0.05** | **0.0464 ± 0.0787 vs 0.0263 ± 0.0393** | **31 vs 31** | **AWGS 2019** | **Zhang, 2024** |
| ^Turicibacter | NS ＞ S | p ＞ 0.05 | 0.006 ± 0.012 vs 0.003 ± 0.004(MD) | 29 vs 17 | AWGS 2019 | Yan, 2023 |
| ^Turicibacter | NS ＞ S | p ＞ 0.05 | 0.306*(DAA) | 36 vs 14 | FNIH | Ponziani, 2021 |
| **^Prevotella** | **NS ＞ S** | **p ＜ 0.05** | **0.156 ± 0.178 vs 0.054 ± 0.062(MD)** | **33 vs 27** | **AWGS 2019** | **Lee, 2022** |
| **^Prevotella** | **NS ＞ S** | **p ＜ 0.05** | **0.0003 ± 0.0006 vs 0 ± 0(MD)** | **31 vs 31** | **AWGS 2019** | **Zhang, 2024** |
| ^Prevotella | NS ＜ S | p ＞ 0.05 | 0.004 ± 0.008 vs 0.009 ± 0.023(MD) | 29 vs 17 | AWGS 2019 | Yan, 2023 |
| ^Prevotella | NS ＞ S | p ＞ 0.05 | 1.604*(DAA) | 17 vs 18 | FNIH | Picca, 2019 |
| ^Prevotella | NS ＞ S | p ＞ 0.05 | 1.204*(DAA) | 36 vs 14 | FNIH | Ponziani, 2021 |
| ^Prevotella | NS ＜ S | p ＞ 0.05 | 0.215 vs 0.269(M) | 104 vs 88 | EWGSOP 2 | Wu, 2022 |
| ^Prevotella | NS ＞ S | p ＞ 0.05 | 0.264 vs 0.171(M) | 142 vs 141 | AWGS 2014 | Liu, 2023 |
| **^Prevotella_9** | **NS ＞ S** | **p ＜ 0.05** | **2.346 vs 0.174(M)** | **21 vs 14** | **AWGS 2019** | **Zhang, 2023** |
| **Prevotella_9** | **NS ＞ S** | **p ＞ 0.05** | **0.103 vs 0.083(M)** | **60 vs 11** | **AWGS 2019** | **Kang, 2021** |
| **^Prevotella_7** | **NS ＞ S** | **p ＜ 0.05** | **3.9246*(DAA)** | **21 vs 14** | **AWGS 2019** | **Zhang, 2023** |
| ^Alloprevotella | NS ＜ S | p ＞ 0.05 | 0 ± 0 vs 0.001 ± 0.003(MD) | 29 vs 17 | AWGS 2019 | Yan, 2023 |
| ^Alloprevotella | NS ＞ S | p ＜ 0.05 | 2.5409 ± 2.932 vs 1.5789 ± 2.1984 | 31 vs 31 | AWGS 2019 | Zhang, 2024 |
| **^Paraprevotella** | **NM ＞ LM** | **p ＜ 0.05** | **0.757 ± 0.136% vs 0.364 ± 0.164%(MD)** | **52 vs 36** | **IWGS** | **Han, 2022** |
| **^Paraprevotella** | **NM ＞ LM** | **p ＜ 0.05** | **0.2051(FDR)** | **52 vs 36** | **IWGS** | **Han, 2022** |
| ^Paraprevotella | NS ＞ S | p ＞ 0.05 | 1.201*(DAA) | 17 vs 18 | FNIH | Picca, 2019 |
| ^Paraprevotella | NS ＞ S | p ＞ 0.05 | -0.661*(DAA) | 36 vs 14 | FNIH | Ponziani, 2021 |
| ^Prevotellaceae_NK3B31_group | NS＞S | p ＜ 0.05 | 0±0 vs 0±0 | 31 vs 31 | AWGS 2019 | Zhang, 2024 |
| **^Odoribacter** | **NM ＞ LM** | **p ＜ 0.05** | **0.394 ± 0.088% vs 0.226 ± 0.065%(MD)** | **52 vs 36** | **IWGS** | **Han, 2022** |
| **^Odoribacter** | **NM ＞ LM** | **p ＜ 0.05** | **0.2801(FDR)** | **52 vs 36** | **IWGS** | **Han, 2022** |
| ^Butyricimonas | NS ＜ S | p ＞ 0.05 | 2.954*(DAA) | 33 vs 27 | AWGS 2019 | Lee, 2022 |
| **^Bacteroides** | **NM ＜ LM** | **p ＜ 0.05** | **30.79 ± 1.99% vs 37.82 ± 2.36%(MD)** | **52 vs 36** | **IWGS** | **Han, 2022** |
| **^Bacteroides** | **NM ＜ LM** | **p ＜ 0.05** | **0.3211(FDR)** | **52 vs 36** | **IWGS** | **Han, 2022** |
| **^Bacteroides** | **NS ＜ S** | **p ＜ 0.05** | **0.017 ± 0.045 vs 0.019 ± 0.021(MD)** | **29 vs 17** | **AWGS 2019** | **Yan, 2023** |
| **^Bacteroides** | **N-SMI ＜ L-SMI** | **p ＜ 0.05** | **-4.736*(DAA)** | **44 vs 25** | **Other** | **Yamamoto, 2022** |
| ^Bacteroides | NS ＞ S | p ＞ 0.05 | 0.513*(DAA) | 17 vs 18 | FNIH | Picca, 2019 |
| ^Bacteroides | NS ＜ S | p ＞ 0.05 | 0.216*(DAA) | 36 vs 14 | FNIH | Ponziani, 2021 |
| ^Bacteroides | NS ＞ S | p ＞ 0.05 | 0.180 vs 0.146(M) | 104 vs 88 | EWGSOP 2 | Wu, 2022 |
| **^Bacteroides** | **NS ＞ S** | **p ＜ 0.05** | **36.403 vs 30.930(M)** | **21 vs 14** | **AWGS 2019** | **Zhang, 2023** |
| Bacteroides | NS ＜ S | p ＞ 0.05 | 0.187 vs 0.232(M) | 142 vs 141 | AWGS 2014 | Liu, 2023 |
| Bacteroides | NS ＜ S | p ＞ 0.05 | 0.345 vs 0.389 (M) | 60 vs 11 | AWGS 2019 | Kang, 2021 |
| ^Parabacteroides | NS ＜ S | p ＞ 0.05 | 0.001 ± 0.003 vs 0.002 ± 0.005(MD) | 29 vs 17 | AWGS 2019 | Yan, 2023 |
| Parabacteroides | NS ＜ S | p ＞ 0.05 | 0.023 vs 0.063(M) | 60 vs 11 | AWGS 2019 | Kang, 2021 |
| Parabacteroides | NS ＜ S | p ＞ 0.05 | 4.431 vs 4.605(M) | 21 vs 14 | AWGS 2019 | Zhang, 2023 |
| ^Parabacteroides | NS ＜ S | p ＞ 0.05 | 3.908*(DAA) | 33 vs 27 | AWGS 2019 | Lee, 2022 |
| ^Parabacteroides | NS ＞ S | p ＞ 0.05 | 0.210*(DAA) | 17 vs 18 | FNIH | Picca, 2019 |
| ^Parabacteroides | NS ＞ S | p ＞ 0.05 | -0.024*(DAA) | 36 vs 14 | FNIH | Ponziani, 2021 |
| **^Barnesiella** | **NM ＞ LM** | **p ＜ 0.05** | **0.764 ± 0.170% vs 0.459 ± 0.238% (MD)** | **52 vs 36** | **IWGS** | **Han, 2022** |
| **^Barnesiella** | **NM ＞ LM** | **p ＜ 0.05** | **0.3954 (FDR)** | **52 vs 36** | **IWGS** | **Han, 2022** |
| ^Alistipes | NS ＜ S | p ＞ 0.05 | 0.002 ± 0.003 vs 0.006 ± 0.017(MD) | 29 vs 17 | AWGS 2019 | Yan, 2023 |
| **^Alistipes** | **NS ＞ S** | **p ＜ 0.05** | **2.501 ± 0.327% vs 1.700 ± 0.390%(MD)** | **52 vs 36** | **IWGS** | **Han, 2022** |
| **^Alistipes** | **NS ＞ S** | **p ＜ 0.05** | **0.3104(FDR)** | **52 vs 36** | **IWGS** | **Han, 2022** |
| Alistipes | NS ＜ S | p ＞ 0.05 | 0.031 vs 0.035(M) | 142 vs 141 | AWGS 2014 | Liu, 2023 |
| Alistipes | NS ＜ S | p ＞ 0.05 | 0.008 vs 0.034(M) | 60 vs 11 | AWGS 2019 | Kang, 2021 |
| Rikenellaceae_unclassified | NS ＞ S | p ＞ 0.05 | 0.010 vs 0.007(M) | 104 vs 88 | EWGSOP 2 | Wu, 2022 |
| **^Flavobacteriaceae_unclassfied** | **NS＜S** | **p ＜ 0.05** | **0 ± 0 vs 0.0008 ± 0.001** | **31 vs 31** | **AWGS 2019** | **Zhang, 2024** |
| **^Oxalobacter** | **NM ＞ LM** | **p ＜ 0.05** | **0.026 ± 0.007% vs 0.012 ± 0.006%(MD)** | **52 vs 36** | **IWGS** | **Han, 2022** |
| **^Oxalobacter** | **NM ＞ LM** | **p ＜ 0.05** | **0.0618(FDR)** | **52 vs 36** | **IWGS** | **Han, 2022** |
| ^Parasutterella | NS ＜ S | p ＞ 0.05 | 0 ± 0 vs 0.001 ± 0.003(MD) | 29 vs 17 | AWGS 2019 | Yan, 2023 |
| ^Sutterella | NS ＞ S | p ＞ 0.05 | 0.405*(DAA) | 17 vs 18 | FNIH | Picca, 2019 |
| **^Escherichia-Shigella** | **NS ＜ S** | **p ＜ 0.05** | **0.069 ± 0.151 vs 0.17 ± 0.228(MD)** | **29 vs 17** | **AWGS 2019** | **Yan, 2023** |
| **^Escherichia-Shigella** | **NS ＜ S** | **p ＜ 0.05** | **0.013 ± 0.007 vs 0.1643 ± 0.0892(MD)** | **21 vs 14** | **AWGS 2019** | **Zhang, 2023** |
| **^Escherichia-Shigella** | **NS ＜ S** | **p ＜ 0.05** | **1.477 vs 16.594(M)** | **21 vs 14** | **AWGS 2019** | **Zhang, 2023** |
| **^Escherichia-Shigella** | **NS ＜ S** | **p ＜ 0.05** | **-4.8696*(DAA)** | **21 vs 14** | **AWGS 2019** | **Zhang, 2023** |
| Escherichia-Shigella | NS ＜ S | p ＞ 0.05 | 0.034 vs 0.074(M) | 60 vs 11 | AWGS 2019 | Kang, 2021 |
| ^Enterobacter | NS ＞ S | p ＞ 0.05 | 0.054 ± 0.147 vs 0.035 ± 0.112(MD) | 29 vs 17 | AWGS 2019 | Yan, 2023 |
| Enterobacteriaceae_unclassified | NS ＞ S | p ＞ 0.05 | 0.058 vs 0.035(M) | 104 vs 88 | EWGSOP 2 | Wu, 2022 |
| **^Klebsiella** | **NS ＜ S** | **p ＜ 0.05** | **-4.1855*(DAA)** | **21 vs 14** | **AWGS 2019** | **Zhang, 2023** |
| ^Klebsiella | NS ＞ S | p ＞ 0.05 | 0.941*(DAA) | 36 vs 14 | FNIH | Ponziani, 2021 |
| ^Klebsieilla | NS ＜ S | p ＞ 0.05 | 0.087 vs 4.170(M) | 21 vs 14 | AWGS 2019 | Zhang, 2023 |
| ^Haemophilus | NS ＞ S | p ＞ 0.05 | 0.001 ± 0.003 vs 0 ± 0.001(MD) | 29 vs 17 | AWGS 2019 | Yan, 2023 |
| ^Haemophilus | NS ＜ S | p ＞ 0.05 | -1.431*(DAA) | 17 vs 18 | FNIH | Picca, 2019 |
| ^Haemophilus | NS ＜ S | p ＞ 0.05 | -0.445*(DAA) | 36 vs 14 | FNIH | Ponziani, 2021 |
| ^Bilophila | NS ＜ S | p ＞ 0.05 | -0.691*(DAA) | 17 vs 18 | FNIH | Picca, 2019 |
| ^Bilophila | NS ＜ S | p ＞ 0.05 | -2.313*(DAA) | 36 vs 14 | FNIH | Ponziani, 2021 |
| **^Eggerthella** | **NM ＜ LM** | **p ＜ 0.05** | **0.018 ± 0.004% vs 0.035 ± 0.008%(MD)** | **52 vs 36** | **IWGS** | **Han, 2022** |
| **^Eggerthella** | **NM ＜ LM** | **p ＜ 0.05** | **0.4230(FDR)** | **52 vs 36** | **IWGS** | **Han, 2022** |
| **^Eggerthella** | **NS ＜ S** | **p ＜ 0.05** | **-2.029*(DAA)** | **17 vs 18** | **FNIH** | **Picca, 2019** |
| ^Eggerthella | NS ＞ S | p ＞ 0.05 | -0.203*(DAA) | 36 vs 14 | FNIH | Ponziani, 2021 |
| ^Adlercreutzia | NS ＜ S | p ＞ 0.05 | 0.002 ± 0.003 vs 0.003 ± 0.004(MD) | 29 vs 17 | AWGS 2019 | Yan, 2023 |
| ^Adlercreutzia | NS ＜ S | p ＞ 0.05 | -0.176*(DAA) | 17 vs 18 | FNIH | Picca, 2019 |
| ^Adlercreutzia | NS ＞ S | p ＞ 0.05 | 0.358*(DAA) | 36 vs 14 | FNIH | Ponziani, 2021 |
| **^Slackia** | **NS ＞ S** | **p ＜ 0.05** | **7.20*(DAA)** | **17 vs 18** | **FNIH** | **Picca, 2019** |
| **^Slackia** | **NS ＞ S** | **p ＜ 0.05** | **9.088*(DAA)** | **36 vs 14** | **FNIH** | **Ponziani, 2021** |
| ^Collinsella | NS ＜ S | p ＞ 0.05 | 0.028 ± 0.033 vs 0.045 ± 0.067(MD) | 29 vs 17 | AWGS 2019 | Yan, 2023 |
| **^Collinsella** | **NS ＞ S** | **p ＜ 0.05** | **3.658*(DAA)** | **21 vs 14** | **AWGS 2019** | **Zhang, 2023** |
| ^Collinsella | NS ＜ S | p ＞ 0.05 | -0.252*(DAA) | 17 vs 18 | FNIH | Picca, 2019 |
| ^Collinsella | NS ＜ S | p ＞ 0.05 | 0.115*(DAA) | 36 vs 14 | FNIH | Ponziani, 2021 |
| ^Bifidobacterium | NS ＞ S | p ＞ 0.05 | 0.087 ± 0.121 vs 0.045 ± 0.032(MD) | 29 vs 17 | AWGS 2019 | Yan, 2023 |
| Bifidobacterium | NS ＞ S | p ＞ 0.05 | 2.520 vs 2.085(M) | 21 vs 14 | AWGS 2019 | Zhang, 2023 |
| Bifidobacterium | NS ＜ S | p ＞ 0.05 | 0.011 vs 0.018(M) | 104 vs 88 | EWGSOP 2 | Wu, 2022 |
| Bifidobacterium | NS ＜ S | p ＞ 0.05 | 0.037 vs 0.074(M) | 60 vs 11 | AWGS 2019 | Kang, 2021 |
| **^Bifidobacterium** | **NS ＜ S** | **p ＜ 0.05** | **0.042 vs 0.058(M)** | **142 vs 141** | **AWGS 2014** | **Liu, 2023** |
| **^Bifidobacterium** | **NS ＜ S** | **p ＜ 0.05** | **1.685*(DAA)** | **17 vs 18** | **FNIH** | **Picca, 2019** |
| ^Bifidobacterium | NS ＜ S | p ＞ 0.05 | 0.274*(DAA) | 36 vs 14 | FNIH | Ponziani, 2021 |
| **^Bifidobacterium** | **NS ＜ S** | **p ＜ 0.05** | **-4.014*(DAA)** | **142 vs 141** | **AWGS 2014** | **Liu, 2023** |
| **^Rothia** | **NS ＜ S** | **p ＜ 0.05** | **0.004(FDR)** | **45 vs 18** | **EWGSOP 2** | **Margiotta, 2021** |
| ^Rothia | NS ＞ S | p ＞ 0.05 | 0.693*(DAA) | 36 vs 14 | FNIH | Ponziani, 2021 |
| ^Atopobium | NS ＜ S | p ＞ 0.05 | -1.326*(DAA) | 17 vs 18 | FNIH | Picca, 2019 |
| ^Atopobium | NS ＜ S | p ＞ 0.05 | -0.948*(DAA) | 36 vs 14 | FNIH | Ponziani, 2021 |
| **^Akkermansia** | **NM ＞ LM** | **p ＜ 0.05** | **0.986 ± 0.311% vs 0.402 ± 0.257%(MD)** | **52 vs 36** | **IWGS** | **Han, 2022** |
| **^Akkermansia** | **NM ＞ LM** | **p ＜ 0.05** | **0.295(FDR)** | **52 vs 36** | **IWGS** | **Han, 2022** |
| ^Akkermansia | NS ＜ S | p ＞ 0.05 | 0.001 ± 0.002 vs 0.002 ± 0.004(MD) | 29 vs 17 | AWGS 2019 | Yan, 2023 |
| **^Akkermansia** | **NS ＜ S** | **p ＜ 0.05** | **0.008 (FDR)** | **45 vs 18** | **EWGSOP 2** | **Margiotta, 2021** |
| ^Akkermansia | NS ＞ S | p ＞ 0.05 | 1.318*(DAA) | 17 vs 18 | FNIH | Picca, 2019 |
| **^Akkermansia** | **NS ＞ S** | **p ＜ 0.05** | **4.376*(DAA)** | **36 vs 14** | **FNIH** | **Ponziani, 2021** |
| **^Fusobacterium** | **NS ＞ S** | **p ＜ 0.05** | **2.520 vs 1.043(M)** | **21 vs 14** | **AWGS 2019** | **Zhang, 2023** |
| **^Pyramidobacter** | **NS ＜ S** | **p ＜ 0.05** | **-4.467*(DAA)** | **17 vs 18** | **FNIH** | **Picca, 2019** |
| ^Methanobrevibacter | NS ＜ S | p ＞ 0.05 | -1.208*(DAA) | 17 vs 18 | FNIH | Picca, 2019 |
| ^Methanobrevibacter | NS ＞ S | p ＞ 0.05 | 1.838*(DAA) | 36 vs 14 | FNIH | Ponziani, 2021 |
| **^candidate_division_TM7_single**  **-cell_isolate_TM7b** | **NS ＜ S** | **p ＜ 0.05** | **-2.8323*(DAA)** | **50 vs 50** | **EWGSOP 2018 AWGS 2019** | **Wang2023** |
| **^Family_XIII_UCG-001** | **NM ＞ LM** | **p ＜ 0.05** | **0.036 ± 0.005% vs 0.011 ± 0.004%(MD)** | **52 vs 36** | **IWGS** | **Han, 2022** |
| ^Family_XIII_UCG-001 | NM ＞ LM | p ＜ 0.05 | 0.0618(FDR) | 52 vs 36 | IWGS | Han, 2022 |
| **^Family_XIII_AD3011_group** | **NM ＞ LM** | **p ＜ 0.05** | **0.067 ± 0.012% vs 0.025 ± 0.006%(MD)** | **52 vs 36** | **IWGS** | **Han, 2022** |
| **^Family_XIII_AD3011_group** | **NM ＞ LM** | **p ＜ 0.05** | **0.0979(FDR)** | **52 vs 36** | **IWGS** | **Han, 2022** |
| ^UCG-001 | NS ＞ S | p ＞ 0.05 | 0.001 ± 0.002 vs 0 ± 0.001(MD) | 29 vs 17 | AWGS 2019 | Yan, 2023 |
| ^UCG-005 | NS ＞ S | p ＞ 0.05 | 0.003 ± 0.003 vs 0.002 ± 0.002(MD) | 29 vs 17 | AWGS 2019 | Yan, 2023 |
| ^UCG-002 | NS ＜ S | p ＞ 0.05 | 0.006 ± 0.01 vs 0.009 ± 0.02(MD) | 29 vs 17 | AWGS 2019 | Yan, 2023 |
| ^UBA1819 | NS ＜ S | p ＞ 0.05 | 0 ± 0 vs 0.002 ± 0.006(MD) | 29 vs 17 | AWGS 2019 | Yan, 2023 |
| ^CAG-352 | NS ＞ S | p ＞ 0.05 | 0.001 ± 0.003 vs 0 ± 0.001(MD) | 29 vs 17 | AWGS 2019 | Yan, 2023 |
| ^RF39 | NS ＞ S | p ＞ 0.05 | 0.003 ± 0.005 vs 0.002 ± 0.003(MD) | 29 vs 17 | AWGS 2019 | Yan, 2023 |
| **^GCA-900066575** | **NS ＜ S** | **p ＜ 0.05** | **0.003 ± 0.0036 vs 0.0138 ± 0.0173** | **31 vs 31** | **AWGS 2019** | **Zhang, 2024** |
| **Species of bacteria** | **Sarcopenia effect** | **P-value** | **Value NS vs S** | **Sample size NS vs S** | **Sarcopenia criteria** | **Ref** |
| **^Bacteroides_eggerthii_DSM_20697** | **NM ＞ LM** | **p ＜ 0.05** | **0.065 ± 0.018 vs 0.011 ± 0.007(MD)** | **52 vs 36** | **IWGS** | **Han, 2022** |
| **^Bacteroides_eggerthii_DSM_20698** | **NM ＞ LM** | **p ＜ 0.05** | **0.7795(FDR)** | **52 vs 36** | **IWGS** | **Han, 2022** |
| **^Bacteroides_fluxus** | **NS ＜ S** | **p ＜ 0.05** | **-3.6171 ± 0.6341 vs -3.5193 ± 0.6237 #(MD)** | **1276 vs 141** | **AWGS 2019** | **Wang, 2022** |
| **^Bacteroides_fluxus** | **NS ＞ S** | **p ＜ 0.05** | **3.2534*(DAA)** | **50 vs 50** | **EWGSOP 2018 AWGS 2019** | **Wang, 2023** |
| **^Bacteroides_massiliensis** | **NS ＞ S** | **p ＜ 0.05** | **3.9668*(DAA)** | **50 vs 50** | **EWGSOP 2018 AWGS 2019** | **Wang, 2023** |
| **^Bacteroides_coprocola** | **NS ＞ S** | **p ＜ 0.05** | **3.5178*(DAA)** | **50 vs 50** | **EWGSOP 2018 AWGS 2019** | **Wang, 2023** |
| **^Bacteroides_coprophilus** | **NS ＞ S** | **p ＜ 0.05** | **3.2718*(DAA)** | **50 vs 50** | **EWGSOP 2018 AWGS 2019** | **Wang, 2023** |
| **^Bacteroides_ovatus** | **S ＜ NS** | **p ＜ 0.05** | **-4.194#(DAA)** | **38 vs 78** | **AWGS 2019** | **Aliwa 2023** |
| ^Bacteroides_caccae | NS ＜ S | p ＞ 0.05 | 0.012 ± 0.020 vs 0.022 ± 0.055(MD) | 12 vs 5 | EWGSOP 1 | Ticinesi, 2020 |
| ^Bacteroides_dorei | NS ＞ S | p ＞ 0.05 | 0.009 ± 0.015 vs 0.003 ± 0.007(MD) | 12 vs 5 | EWGSOP 1 | Ticinesi, 2020 |
| ^Bacteroides_fragilis | NS ＜ S | p ＞ 0.05 | 0.007 ± 0.013 vs 0.046 ± 0.114(MD) | 12 vs 5 | EWGSOP 1 | Ticinesi, 2020 |
| Bacteroides_fragilis | S ＞ NS | p ＞ 0.05 | 3.809#(DAA) | 38 vs 78 | AWGS 2019 | Aliwa 2023 |
| ^Bacteroides_uniformis | NS ＜ S | p ＞ 0.05 | 0.079 ± 0.105 vs 0.137 ± 0.139(MD) | 12 vs 5 | EWGSOP 1 | Ticinesi, 2020 |
| ^Bacteroides_vulgatus | NS ＞ S | p ＞ 0.05 | 0.049 ± 0.064 vs 0.014 ± 0.022(MD) | 12 vs 5 | EWGSOP 1 | Ticinesi, 2020 |
| **^Bacteroides_vulgatus** | **NS ＜ S** | **p ＜ 0.05** | **-4.042*(DAA)** | **142 vs 141** | **AWGS 2014** | **Liu, 2023** |
| **^Bacteroidales_bacterium_ph8** | **NS ＞ S** | **p ＜ 0.05** | **3.1427*(DAA)** | **50 vs 50** | **EWGSOP 2018 AWGS 2019** | **Wang2023** |
| ^Parabacteroides_distasonis | NS ＜ S | p ＞ 0.05 | 0.019 ± 0.028 vs 0.085 ± 0.169(MD) | 12 vs 5 | EWGSOP 1 | Ticinesi, 2020 |
| ^Parabacteroides_merdae | NS ＜ S | p ＞ 0.05 | 0.012 ± 0.017 vs 0.016 ± 0.031(MD) | 12 vs 5 | EWGSOP 1 | Ticinesi, 2020 |
| **^Parabacteroides_goldsteinii_CL02T12C30** | **NM ＞ LM** | **p ＜ 0.05** | **0.009 ± 0.003% vs 0.008 ± 0.004%(MD)** | **52 vs 36** | **IWGS** | **Han, 2022** |
| **^Parabacteroides_goldsteinii_CL02T12C30** | **NM ＞ LM** | **p ＜ 0.05** | **>0.9999(FDR)** | **52 vs 36** | **IWGS** | **Han, 2022** |
| **^Parabacteroides_johnsonii_CL02T12C29** | **NM ＞ LM** | **p ＜ 0.05** | **0.017 ± 0.013% vs 0.004 ± 0.004%(MD)** | **52 vs 36** | **IWGS** | **Han, 2022** |
| **^Parabacteroides_johnsonii_CL02T12C29** | **NM ＞ LM** | **p ＜ 0.05** | **>0.9999(FDR)** | **52 vs 36** | **IWGS** | **Han, 2022** |
| **^Parabacteroides_sp.** | **NS ＜ S** | **p ＜ 0.05** | **0.008 ± 0.006 vs 0.017 ± 0.014(MD)** | **33 vs 27** | **AWGS 2019** | **Lee, 2022** |
| ^Parabacteroides_sp. | NS ＜ S | p ＞ 0.05 | -3.981*(DAA) | 33 vs 27 | AWGS 2019 | Lee, 2022 |
| **^Prevotella_copri** | **NS ＞ S** | **p ＜ 0.05** | **0.134 ± 0.167 vs 0.029 ± 0.040(MD)** | **33 vs 27** | **AWGS 2019** | **Lee, 2022** |
| **^Prevotella_copri** | **NS ＞ S** | **p ＜ 0.05** | **4.3727*(DAA)** | **50 vs 50** | **EWGSOP 2018 AWGS 2019** | **Wang2023** |
| **^Prevotella_copri** | **NS ＞ S** | **p ＜ 0.05** | **4.42*(DAA)** | **142 vs 141** | **AWGS 2014** | **Liu, 2023** |
| **^Prevotella_copri** | **NS ＞ S** | **p ＜ 0.05** | **0.2077 vs 0.1503(M)** | **142 vs 141** | **AWGS 2014** | **Liu, 2023** |
| **^Prevotella_sp.** | **NS ＞ S** | **p ＜ 0.05** | **2.24*(DAA)** | **31 vs 31** | **AWGS 2019** | **Zhang, 2024** |
| **^Prevotellaceae_NK3B31_groupsp.** | **NS ＞ S** | **p ＜ 0.05** | **3.02*(DAA)** | **31 vs 31** | **AWGS 2019** | **Zhang, 2024** |
| **^Prevotellaceae_sp.** | **NS ＞ S** | **p ＜ 0.05** | **3.26*(DAA)** | **31 vs 31** | **AWGS 2019** | **Zhang, 2024** |
| **^Alloprevotella_sp.** | **NS ＞ S** | **p ＜ 0.05** | **3.75*(DAA)** | **31 vs 31** | **AWGS 2019** | **Zhang, 2024** |
| **^Coprobacter_secundus** | **NS ＜ S** | **p ＜ 0.05** | **-4.4058 ± 0.5194 vs -4.2434 ± 0.5471 #(MD)** | **1276 vs 141** | **AWGS 2019** | **Wang, 2022** |
| **^Barnesiella_intestinihominis** | **NS ＞ S** | **p ＜ 0.05** | **3.4379*(DAA)** | **50 vs 50** | **EWGSOP 2018 AWGS 2019** | **Wang, 2023** |
| ^Barnesiella_intestinihominis | NS ＞ S | p ＞ 0.05 | 0.018 ± 0.024 vs 0.014 ± 0.036(MD) | 12 vs 5 | EWGSOP 1 | Ticinesi, 2020 |
| ^Alistipes_onderdonkii | NS ＜ S | p ＞ 0.05 | 0.007 ± 0.011 vs 0.049 ± 0.124(MD) | 12 vs 5 | EWGSOP 1 | Ticinesi, 2020 |
| **^Alistipes_shahii** | **NS ＞ S** | **p ＜ 0.05** | **0.009 ± 0.013 vs 0.001 ± 0.002(MD)** | **12 vs 5** | **EWGSOP 1** | **Ticinesi, 2020** |
| ^Butyricimonas_sp. | NS ＜ S | p ＞ 0.05 | -2.926*(DAA) | 33 vs 27 | AWGS 2019 | Lee, 2022 |
| **^Gabonia_massiliensis** | **NM ＞ LM** | **p ＜ 0.05** | **0.001 ± 0.001% vs 0.00 ± 0.00%(MD)** | **52 vs 36** | **IWGS** | **Han, 2022** |
| **^Gabonia_massiliensis** | **NM ＞ LM** | **p ＜ 0.05** | **0.9185(FDR)** | **52 vs 36** | **IWGS** | **Han, 2022** |
| **^Flavobacteriaceaesp.** | **NS＜S** | **p ＜ 0.05** | **-2.49*(DAA)** | **31 vs 31** | **AWGS 2019** | **Zhang, 2024** |
| **^Faecalibacterium_prausnitzii** | **NM ＞ LM** | **p ＜ 0.05** | **0.006 ± 0.001% vs 0.002 ± 0.001%(MD)** | **52 vs 36** | **IWGS** | **Han, 2022** |
| **^Faecalibacterium_prausnitzii** | **NM ＞ LM** | **p ＜ 0.05** | **0.9185(FDR)** | **52 vs 36** | **IWGS** | **Han, 2022** |
| **^Faecalibacterium_prausnitzii** | **NS ＞ S** | **p ＜ 0.05** | **0.058 ± 0.068 vs 0.016 ± 0.039(MD)** | **12 vs 5** | **EWGSOP 1** | **Ticinesi, 2020** |
| **^Faecalibacterium_prausnitzii** | **NS ＜ S** | **p ＜ 0.05** | **-3.363*(DAA)** | **142 vs 141** | **AWGS 2014** | **Liu, 2023** |
| ^Anaerotruncus_sp. | NS ＜ S | p ＞ 0.05 | -2.309*(DAA) | 33 vs 27 | AWGS 2019 | Lee, 2022 |
| **^Subdoligranulum_variabile** | **NS ＜ S** | **p ＜ 0.05** | **-3.6285*(DAA)** | **50 vs 50** | **EWGSOP 2018 AWGS 2019** | **Wang2023** |
| ^Subdoligranulum_sp. | NS ＞ S | p ＞ 0.05 | 0.002 ± 0.002 vs 0.002 ± 0.004(MD) | 12 vs 5 | EWGSOP 1 | Ticinesi, 2020 |
| ^Flavonifractor_plautii | NS ＜ S | p ＞ 0.05 | 0.006 ± 0.005 vs 0.010 ± 0.008(MD) | 12 vs 5 | EWGSOP 1 | Ticinesi, 2020 |
| ^Ruminococcus_bromii | NS ＜ S | p ＞ 0.05 | 0.006 ± 0.012 vs 0.008 ± 0.015(MD) | 12 vs 5 | EWGSOP 1 | Ticinesi, 2020 |
| ^Ruminococcus_gnavus | NS ＜ S | p ＞ 0.05 | 0.001 ± 0.002 vs 0.014 ± 0.032(MD) | 12 vs 5 | EWGSOP 1 | Ticinesi, 2020 |
| Ruminococcus_2 | NS ＜ S | p ＞ 0.05 | 0.013 vs 0.016(M) | 60 vs 11 | AWGS 2019 | Kang, 2021 |
| Ruminococcaceae_UCG-002 | NS ＜ S | p ＞ 0.05 | 0.007 vs 0.024(M) | 60 vs 11 | AWGS 2019 | Kang, 2021 |
| **^Romboutsia_sp.** | **NS ＜ S** | **p ＜ 0.05** | **-2.79*(DAA)** | **31 vs 31** | **AWGS 2019** | **Zhang, 2024** |
| **^Clostridium_symbiosum** | **NS ＜ S** | **p ＜ 0.05** | **-4.2297 ± 0.5832 vs -3.9722 ± 0.644 #(MD)** | **1276 vs 141** | **AWGS 2019** | **Wang, 2022** |
| **^Clostridium_citroniae** | **NS ＜ S** | **p ＜ 0.05** | **-3.9024 ± 0.422 vs -3.757 ± 0.3927 #(MD)** | **1276 vs 141** | **AWGS 2019** | **Wang, 2022** |
| **^Clostridium_sp_L2_50** | **NS ＞ S** | **p ＜ 0.05** | **3.3518*(DAA)** | **50 vs 50** | **EWGSOP 2018 AWGS 2019** | **Wang, 2023** |
| **^Lachnoclostridium_phocaeense** | **NM ＜ LM** | **p ＜ 0.05** | **0.00 ± 0.00% vs 0.0003 ± 0.0001%(MD)** | **52 vs 36** | **IWGS** | **Han, 2022** |
| **^Lachnoclostridium_phocaeense** | **NM ＜ LM** | **p ＜ 0.05** | **0.7795(FDR)** | **52 vs 36** | **IWGS** | **Han, 2022** |
| ^Roseburia_intestinalis | NS ＞ S | p ＞ 0.05 | 0.007 ± 0.014 vs 0.002 ± 0.004(MD) | 12 vs 5 | EWGSOP 1 | Ticinesi, 2020 |
| **^Roseburia_inulinivorans** | **NS ＞ S** | **p ＜ 0.05** | **0.005 ± 0.007 vs 0 ± 0(MD)** | **12 vs 5** | **EWGSOP 1** | **Ticinesi, 2020** |
| **^Eubacterium_rectale** | **NS ＞ S** | **p ＜ 0.05** | **3.71*(DAA)** | **142 vs 141** | **AWGS 2014** | **Liu, 2023** |
| **^Lachnospiraceae_bacterium_2_1_58FAA** | **NS ＜ S** | **p ＜ 0.05** | **-3.1919*(DAA)** | **50 vs 50** | **EWGSOP 2018 AWGS 2019** | **Wang, 2023** |
| **^Hungatella_effluvii** | **NS ＜ S** | **p ＜ 0.05** | **-4.1692 ± 0.651 vs -3.9844 ± 0.6456 #(MD)** | **1276 vs 141** | **AWGS 2019** | **Wang, 2022** |
| ^Phascolarctobacterium_sp. | NS ＜ S | p ＞ 0.05 | -3.148*(DAA) | 33 vs 27 | AWGS 2019 | Lee, 2022 |
| **^Phascolarctobacterium_succinatutens** | **NS ＞ S** | **p ＜ 0.05** | **3.4625*(DAA)** | **50 vs 50** | **EWGSOP 2018 AWGS 2019** | **Wang2023** |
| ^Dialister_sp. | NS ＞ S | p ＞ 0.05 | 3.551*(DAA) | 33 vs 27 | AWGS 2019 | Lee, 2022 |
| **^Dialister_succinatiphilus** | **NS ＜ S** | **p ＜ 0.05** | **-3.1119*(DAA)** | **50 vs 50** | **EWGSOP 2018 AWGS 2019** | **Wang, 2023** |
| **^Mitsuokella_multacida** | **NS ＞ S** | **p ＜ 0.05** | **2.3739*(DAA)** | **50 vs 50** | **EWGSOP 2018 AWGS 2019** | **Wang, 2023** |
| **^Mitsuokella_sp.** | **NS ＞ S** | **p ＜ 0.05** | **2.81*(DAA)** | **31 vs 31** | **AWGS 2019** | **Zhang, 2024** |
| **^Selenomonas_sp.** | **NS ＜ S** | **p ＜ 0.05** | **-2.69*(DAA)** | **31 vs 31** | **AWGS 2019** | **Zhang, 2024** |
| **^Anacrovibrio_sp.** | **NS ＞ S** | **p ＜ 0.05** | **3.49*(DAA)** | **31 vs 31** | **AWGS 2019** | **Zhang, 2024** |
| **^Absiella_innocuum** | **NS ＜ S** | **p ＜ 0.05** | **-4.2699 ± 0.5387 vs -4.0639 ± 0.4867 #(MD)** | **1276 vs 141** | **AWGS 2019** | **Wang, 2022** |
| **^Erysipelotrichaceae_UCG_003_sp.** | **NS ＜ S** | **p ＜ 0.05** | **-2.03*(DAA)** | **31 vs 31** | **AWGS 2019** | **Zhang, 2024** |
| **^Catenibacterium_mitsuokai** | **NS ＜ S** | **p ＜ 0.05** | **-2.858*(DAA)** | **50 vs 50** | **EWGSOP 2018 AWGS 2019** | **Wang, 2023** |
| **^Catenibacterium_sp.** | **NS ＜ S** | **p ＜ 0.05** | **-3.16*(DAA)** | **31 vs 31** | **AWGS 2019** | **Zhang, 2024** |
| **^Lactobacillus_fermentum** | **NS ＜ S** | **p ＜ 0.05** | **-2.4047*(DAA)** | **50 vs 50** | **EWGSOP 2018 AWGS 2019** | **Wang, 2023** |
| **^Enterococcus_hirae** | **NS ＜ S** | **p ＜ 0.05** | **-3.7043*(DAA)** | **21 vs 17** | **AWGS 2019** | **Zhang, 2023** |
| **^Desulfovibrio_piger** | **NS ＜ S** | **p ＜ 0.05** | **-4.6982 ± 1.6194 vs -4.424 ± 1.3854 #(MD)** | **1276 vs 141** | **AWGS 2019** | **Wang, 2022** |
| **^Parasutterella_excrementihominis** | **NS ＞ S** | **p ＜ 0.05** | **2.3739*(DAA)** | **50 vs 50** | **EWGSOP 2018 AWGS 2019** | **Wang, 2023** |
| ^Sutterella_sp. | S ＞ NS | p ＞ 0.05 | 3.879#(DAA) | 38 vs 78 | AWGS 2019 | Aliwa 2023 |
| **^Burkholderiales_bacterium_1_1_47** | **NS ＞ S** | **p ＜ 0.05** | **2.2694*(DAA)** | **50 vs 50** | **EWGSOP 2018 AWGS 2019** | **Wang, 2023** |
| **^Escherichia_coli_TOP293-4** | **NS ＜ S** | **p ＜ 0.05** | **0.0001 ± 0.0001 vs 0.0002 ± 0.0001(MD)** | **21 vs 14** | **AWGS 2019** | **Zhang, 2023** |
| **^Escherichia_coli** | **NS ＜ S** | **p ＜ 0.05** | **0.0009 ± 0.0004 vs 0.0076 ± 0.0031(MD)** | **21 vs 14** | **AWGS 2019** | **Zhang, 2023** |
| **^Escherichia_coli** | **NS ＜ S** | **p ＜ 0.05** | **-3.7275*(DAA)** | **21 vs 18** | **AWGS 2019** | **Zhang, 2023** |
| ^Escherichia_coli | NS ＜ S | p ＞ 0.05 | 0.001 ± 0.001 vs 0.016 ± 0.038(MD) | 12 vs 5 | EWGSOP 1 | Ticinesi, 2020 |
| **^Eggerthella_lenta** | **NS ＜ S** | **p ＜ 0.05** | **-2.6999*(DAA)** | **50 vs 50** | **EWGSOP 2018 AWGS 2019** | **Wang, 2023** |
| **^Collinsella_aerofaciens** | **NS ＜ S** | **p ＜ 0.05** | **-3.3702*(DAA)** | **50 vs 50** | **EWGSOP 2018 AWGS 2019** | **Wang, 2023** |
| **^Collinsellasp.** | **NS ＞ S** | **p ＜ 0.05** | **3.641*(DAA)** | **21 vs 15** | **AWGS 2019** | **Zhang, 2023** |
| **^Bifidobacterium_longum** | **NS ＞ S** | **p ＜ 0.05** | **4.0529*(DAA)** | **50 vs 50** | **EWGSOP 2018 AWGS 2019** | **Wang, 2023** |
| ^Bifidobacterium_longum | NS ＜ S | p ＞ 0.05 | 0.001 ± 0.003 vs 0.006 ± 0.009(MD) | 12 vs 5 | EWGSOP 1 | Ticinesi, 2020 |
| ^Akkermansia_muciniphila | NS ＜ S | p ＞ 0.05 | 0.000 ± 0.001 vs 0.032 ± 0.083(MD) | 12 vs 5 | EWGSOP 1 | Ticinesi, 2020 |
| ^Veillonella_parvula | S ＞ NS | p ＞ 0.05 | 3.852#(DAA) | 38 vs 78 | AWGS 2019 | Aliwa 2023 |
| ^Blautia_marseille | S ＞ NS | p ＞ 0.05 | 3.739#(DAA) | 38 vs 78 | AWGS 2019 | Aliwa 2023 |
| **^Fusobacterium_periodonticum** | **NS ＜ S** | **p ＜ 0.05** | **-3.0197*(DAA)** | **50 vs 50** | **EWGSOP 2018 AWGS 2019** | **Wang, 2023** |

Legend: * : Log2FC; #: Log10FC; ^ : a specific change in relative abundance explicitly given in the article; DAA: differential abundance analysis; M: mean; MD: mean±standard deviation; FDR: false discovery rate; S: sarcopenia; NS: non-sarcopenia; LM: low muscle mass; NM: normal muscle mass; EWGSOP: European Working Group on Sarcopenia in Older People; IWGS: International Working Group on Sarcopenia; AWGS: Asian Working Group for Sarcopenia Guidelines; FNIH: Foundation for the National Institutes of Health sarcopenia project. Bold lines have a level of significance p < 0.05.
