# Supplementary material for: Comparison of the gut microbiota in older people with and without sarcopenia: a systematic review and meta-analysis
Source: Front Cell Infect Microbiol. 2025 Apr 28;15:1480293. doi: 10.3389/fcimb.2025.1480293 (PMC12066693; doi:10.3389/fcimb.2025.1480293)
Supplement: Supplementary file 1 [file DataSheet1.zip › Supplementary materials/Supplemental Figure 2. Sensitivity analysis..pdf]

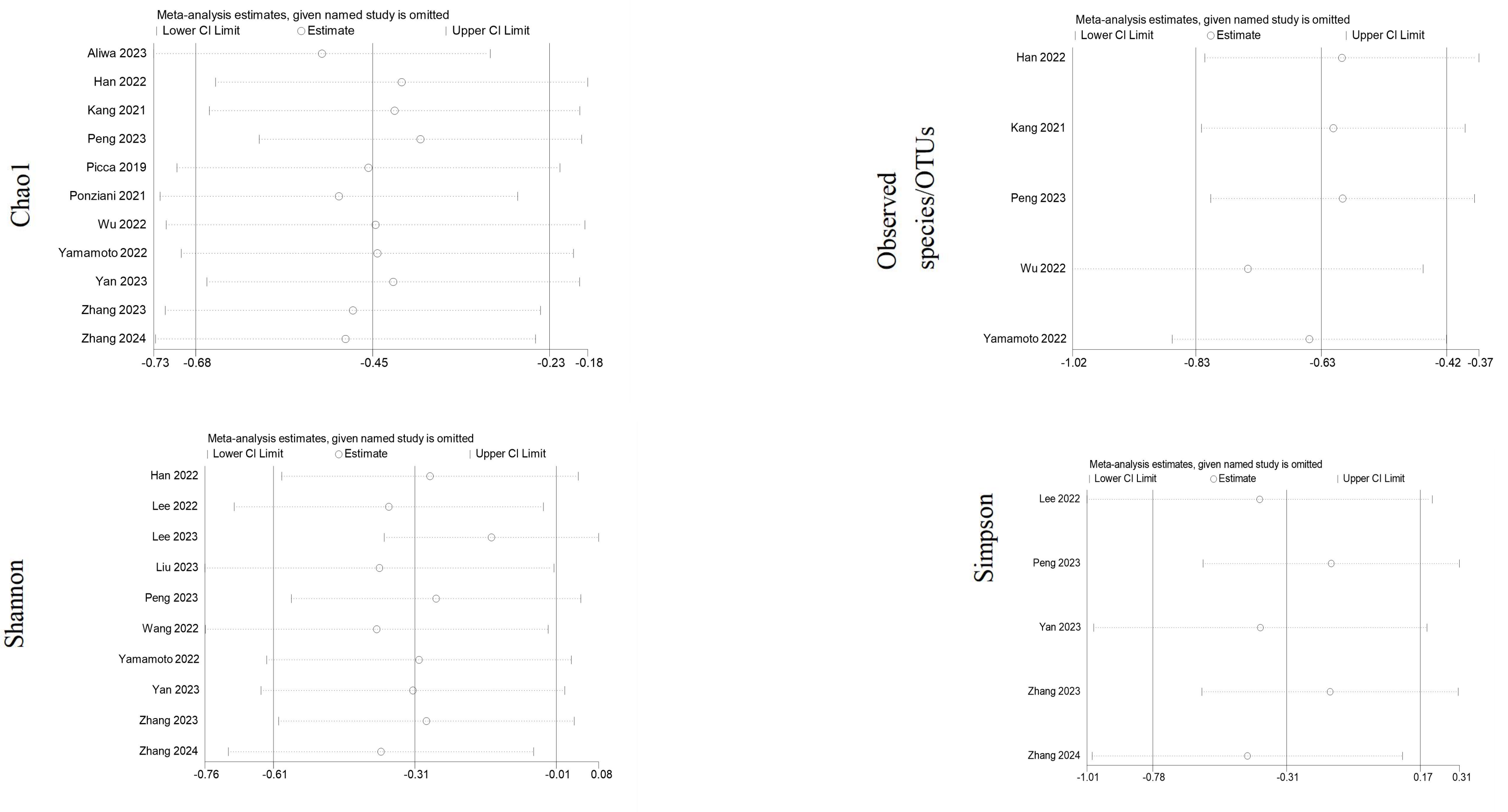

Supplemental Figure 2. Sensitivity analysis: to test the robustness of the results of Chao1, Shannon, Simpson, and Observed species/OTUs.
