# Supplementary material for: Comparison of the gut microbiota in older people with and without sarcopenia: a systematic review and meta-analysis
Source: Front Cell Infect Microbiol. 2025 Apr 28;15:1480293. doi: 10.3389/fcimb.2025.1480293 (PMC12066693; doi:10.3389/fcimb.2025.1480293)
Supplement: Supplementary file 1 [file DataSheet1.zip › Supplementary materials/Supplemental Table 1. Stool sample processing method..docx]

**Supplemental Table 1.** Stool sample processing methods in the included studies.

| **Study** | **Collection & handling by participant** | **Long-term storage** | **DNA extraction method** |
| --- | --- | --- | --- |
| Aliwa2023 | Study participants were provided with sterile dry screw-top containers for collecting stool samples. | Stool samples were received and frozen at -80 °C, waiting for further analysis. | DNA was isolated using a MagNA Pure LC DNA isolation kit (Roche, Mannheim, Germany) as per the manufacturer’s protocol. |
| Han 2022 | not mentioned | Fecal samples were transported to our lab in cold storage (4–7 °C) within 24 h of collection. The feces werethen aliquoted and stored at − 80 °C. | The stool DNA was extracted with Mobio PowerFecal DNA Isolation Kit. |
| Kang 2021 | Fecal samples (several grams) were collected into sterile 2 mL vials containing DET (dimethyl sulfoxide) buffer in accordance with the field sampling protocols of Allwegene Technology, Beijing, China. | Samples were stored at − 80 °C until further analysis. | Total bacterial genomic DNA was extracted from fecal samples using the PowerSoil DNA Isolation Kit(MoBio Laboratories, CA, USA) following manufacturer’s instructions. |
| Lee 2023 | not mentioned | not mentioned | Microbial genomic DNAs were extracted by using the QIAamp DNA Stool Mini Kit (Qiagen, Hilden, Germany). |
| Lee 2022 | Fresh fecal samples were self-collected at home using sterile swabs, immediately transferred to sterile cryogenic tubes, and stored in sealed ice packs until the next clinical visit. | The samples were then stored at −80 ◦C. | total bacterial genomic DNA extraction using a Maxwell RSC PureFood GMO and Authentication Kit (Promega, Madison, WI, USA) according to the manufacturer's instructions. |
| Liu 2023 | Special collection con- tainer provided by the Beijing Quantitative Health Co., Ltd. company was used to collect stool. This contained a solution for preventing the degradation of DNA. Among the DNA solution, 0.15– 0.35 mol/L disodium ethylenediaminetetraacetic acid, sodium chloride and 12–28% dimethyl sulfoxide are dissolved, and the pH is adjusted to 6.0–9.0. | not mentioned | DNA from different samples was extracted using The E.Z.N.A.® Stool DNA Kit (D4015-02, Omega, Inc., USA) according to manufacturer’s instructions. |
| Margiotta 2021 | Each volunteer had to collect feces at home on the day preceding the visit, using 20 mL plastic sterile stool collection containers, and to place it in their own freezer at −18/−20 ◦C overnight. | Feces was stored at −80 ◦C in our laboratories until analysis. | Bacterial DNAs were extracted from 50 mg of fecal sample using the FastDNA™ SPIN Kit for Soil (MP Biomedicals, Lucerna, Switzerland) according to the manufacturer’s instructions. |
| Peng 2023 | Fecal samples of the patients were collected within one hour of excretion in the morning during hospitalization. | Fecal samples were stored at -80°C in preservation tubes before being delivered to the detected center. | Microbial genomic DNA of fecal samples was extracted by the  Quant-iT PicoGreen dsDNA Assay Kit (Invitrogen) according to  the manufacturer’s instructions. |
| Picca 2020 | Stool samples were collected at home in a commercial sterile, dry screw-top container. | Upon collection, stool samples were delivered to the Human Microbiome Unit at the Bambino Gesù Children’s Hospital (Rome, Italy) and immediately frozen at −80 ◦C until further processing. | Total genome DNA was extracted from fecal samples using the QIAmp Fast DNA Stool mini kit (Qiagen, Germany), according to the manufacturer’s instructions. |
| Ponziani 2021 | Fecal samples were collected at home in a commercial sterile, dry screw-top container. | Fecal samples were immediately frozen at −80 °C until further processing. | DNA was extracted from stool samples using a QIAmp Fast DNA Stool mini kit (Qiagen, Germany), following the manufacturer’s instructions. |
| Ticinesi 2020 | The samples were self-collected in the morning by spontaneous evacuation and stored at room temperature in stool nucleic acid collection and preservation tubes (Norgen Biotek, Thorold, Niagara, Ontario, Canada) containing 2 mL of preservative and inactivating solution. | Samples were then delivered by participants to the research center within 24 h and then refrigerated at −22 ◦C. | Bacterial DNA was extracted from fecal samples using the QIAamp Fast DNA Stool Mini kit following the manufacturer’s instructions (Qiagen Ltd., Strasse, Germany). |
| Wang 2022 | Stool samples from the participants were collected at the recruitment site. | Collected stool samples were frozen immediately, transported on dry ice within 20 min, and stored at 80°C until DNA extraction. | In accordance with the manufac- turer’s protocol, 200 mg of stool was used for DNA exaction using the Magen HiPure Soil DNA Kit (Magen, Guangzhou,China). |
| Wang 2023 | Soybean-sized fecal samples for gut microbiota detection were collected in test tubes with a stabilizing solution. | Samples were transported to the laboratory within 48 h, and stored at −80 ◦C. | Fecal bacterial DNA was extracted using the DNeasy PowerSoil Pro Kit (Qiagen, Germantown, MD, USA) according to the manufacturer’s instructions. |
| Wu 2022 | A total of 10 g of a fresh fecal sample from each person was collected and dissolved in 90mL of sterile PBS (0.1 mol/L, pH 4.7). The samples were then stirred evenly and filtered three times using auto- claved double gauze to collect the filtrate (bacterial suspension) for inoculation. | not mentioned | The MicroElute Genomic DNA Kit (D3096-01, Omega, Inc., USA) weas used to extract DNA from different samples. |
| Yamamoto 2022 | Samples were collected at home (n = 18) or the hospital (n = 51). | not mentioned | DNA from stool samples was extracted using the DNeasy PowerSoil Kit (Qiagen, Hilden, Germany). |
| Yan 2023 | Stool samples from a total of 46 participants(17 from sarcopenia patients and 29 from non-sarcopenia patients) were collected in sterile disposable samplers. | All the above samples were stored a −80 ◦C in the refrigerator for further gut microbiota and SCFA analysis. | Genomic DNA was extracted from stool samples according to the kit instructions (Omega Bio-tek, Norcross, GA, U.S.). |
| Zhang 2023 | Fecal samples were collected from the research participants in sterile stool containers in the early morning, were kept cold, and transported to the laboratory within 2 h of collection.The samples were divided into three sterilized lyophilization tubes, with approximately 3 g in each tube, and immediately stored at  −80 ◦C for subsequent analysis. | The homogenized samples were stored at −80 ◦C. | The fecal DNA was isolated with a QIAamp DNA Stool Mini Kit (Qiagen, Germany). |
| Zhang 2024 | Approximately 1 g of fresh feces was collectedfrom the subjects and put into a storage tube (EG-0150, Xiamen Treatgut Biotechnology Co., Ltd., Xiamen, China) containing 2.5 ml of storage solution at room temperature. | The lid was tightened,shaken up and down, and put into a refrigerator. | A QlAamp Fast DNA Stool Mini Kit (QIAGEN) was used to extract fecal DNA, and a Multiskan™ GO Enzyme Labeller (Thermo Fisher Scientific) was used to quantifythe DNA concentrationand assess DNA purity. DNA integrity was determined by 1 % agarose gel elec trophoresis. |
