# Supplementary material for: Comparison of the gut microbiota in older people with and without sarcopenia: a systematic review and meta-analysis
Source: Front Cell Infect Microbiol. 2025 Apr 28;15:1480293. doi: 10.3389/fcimb.2025.1480293 (PMC12066693; doi:10.3389/fcimb.2025.1480293)
Supplement: Supplementary file 1 [file DataSheet1.zip › Supplementary materials/Supplemental Table 3. Egger's regression test..docx]

**Supplemental Table 3.** Publish bias assessment by Egger’s regression test in α-diversity indexes.

| **α-diversity indexes** | **t** | **df** | **p-value** |
| --- | --- | --- | --- |
| Chao 1 | -0.61 | 10 | 0.559 |
| Observed species/OTUs | -2.82 | 4 | 0.067 |
| Shannon index | -2.98 | 9 | 0.018 |
| Simpson index | -3.52 | 4 | 0.039 |
